# Survival of patients with colorectal or pancreatic cancer who received *UGT1A1* genotype-guided dosing of irinotecan in the Netherlands (2017–2024): a retrospective, multicentre cohort study

**DOI:** 10.1016/j.lanepe.2026.101629

**Published:** 2026-02-23

**Authors:** Sofía L.J. Peeters, Niels Heersche, Doortje Böhm, Stefan Böhringer, Roselien Guiljam, Emma C. Hulshof, Femke M. de Man, Mirjam de With, Marije Joosse, Gysella Oomens, Qiong-Yi Wu, Aisha Osman, Sander Bins, Irene E.G. van Hellemond, Brigitte C.M. Haberkorn, Arjan J. Verschoor, Miriam L. Wumkes, Ron H.N. van Schaik, Anna M. Thijs, Hans Gelderblom, Henk-Jan Guchelaar, Ron H.J. Mathijssen, Maarten J. Deenen

**Affiliations:** a Department of Clinical Pharmacy, Catharina Hospital, Eindhoven, the Netherlands; b Department of Clinical Pharmacy and Toxicology, Leiden University Medical Centre Leiden, the Netherlands; c Department of Medical Oncology, Erasmus Medical Centre Cancer Institute, Rotterdam, the Netherlands; d Department of Clinical Chemistry, Erasmus University Medical Centre, Rotterdam, the Netherlands; e Department of Biomedical Data Sciences, Leiden University Medical Centre, Leiden, Netherlands; f Department of Medical Oncology, Catharina Hospital, Eindhoven, the Netherlands; g Department of Medical Oncology, Maasstad Hospital, Rotterdam, the Netherlands; h Department of Medical Oncology, Reinier de Graaf Gasthuis, Delft, the Netherlands; i Department of Medical Oncology, Jeroen Bosch Hospital, ‘s Hertogenbosch, the Netherlands; j Department of Medical Oncology, Leiden University Medical Centre, Leiden, the Netherlands

**Keywords:** UGT1A1, Irinotecan, Pharmacogenetics, Precision dosing, Colorectal cancer, Pancreatic cancer

## Abstract

**Background:**

*UGT1A1* genotype-guided dosing reduces severe toxicity in UGT1A1 poor metaboliser (PM) patients treated with irinotecan. However, the impact of a dose reduction on survival remains unknown. This study evaluated whether upfront 30% dose reductions of irinotecan in UGT1A1 PMs affect survival by comparing progression-free survival (PFS) and overall survival (OS) between 30% dose-reduced PMs and fully-dosed UGT1A1 intermediate and normal metaboliser (IM/NM) patients.

**Methods:**

We conducted a retrospective, multicentre cohort study in patients with pancreatic cancer or colorectal cancer treated with *UGT1A1* genotype-guided irinotecan dosing at six Dutch hospitals between August 2017 and April 2024. Patients were included in the primary analysis if irinotecan was dosed according to *UGT1A1* genotype (i.e. an initial 100% ± 10% dose intensity for IM/NMs and an initial 70% ± 10% dose intensity for PMs) in at least cycle 1. Survival analyses for PFS and OS were performed using Kaplan–Meier estimates and univariable and multivariable Cox regressions, stratified by tumour type. Safety was also assessed.

**Findings:**

The primary analysis included 779 patients, 76 (9.8%) of whom were PMs. The median follow-up was 27.8 months (95% CI 15.2–31.6). No significant differences in PFS and OS rates were found between 30% dose-reduced PMs and fully-dosed IM/NMs (stratified log-rank test: PFS: *P* = 0.54; OS: *P* = 0.42). In stratified Cox regression analyses, the adjusted hazard ratio of PMs *vs* IM/NMs was 1.02 (95% CI 0.78–1.32; *P* = 0.90) for PFS and 1.10 (95% CI 0.82–1.48; *P* = 0.51) for OS, indicating no significant differences exist in PFS or OS between 30% dose-reduced PMs and fully-dosed IM/NMs. Severe toxicity rates were comparable between 30% dose-reduced PMs and fully-dosed IM/NMs (*P* = 0.59).

**Interpretation:**

An upfront 30% dose reduction of irinotecan in UGT1A1 PMs does not lead to statistically significant differences in survival outcomes compared to fully-dosed IM/NMs. Therefore, *UGT1A1* genotype-guided dosing of irinotecan can be confidently performed to improve patient safety.

**Funding:**

No funding.

**Research in context**

**Evidence before this study**

We identified ongoing and published studies on efficacy outcomes of dose-reduced irinotecan in UGT1A1 poor metabolisers before initiation of this study and during the development of this manuscript. To this end, we searched ClinicalTrials.gov and PubMed on 6 February 2026 with the terms “irinotecan” AND “UGT1A1”, “UGT1A1” AND “response OR efficacy OR survival”, with no limitations on language or publication date. Only studies were selected in which the irinotecan dose was prospectively reduced in UGT1A1 poor metabolisers and in which survival outcomes were reported. We identified one study in Asia that reported survival outcomes of 47 UGT1A1 poor metabolisers treated with mCAPIRI or FOLFIRI who received an upfront 17–25% dose reduction of irinotecan. It concluded that treatment effectiveness was independent of *UGT1A1* genotype. Additionally, these authors suggested that a higher irinotecan dose reduction of 35–40% may be necessary in poor metabolisers due to higher rates of severe toxicities. We identified no ongoing clinical trials on UGT1A1 and treatment effectiveness.

**Added value of this study**

To the best of our knowledge, this study represents the first large-scale study on survival outcomes after *UGT1A1* genotype-guided dose individualisation of irinotecan in a real-world setting. We showed that no significant differences exist in the progression-free and overall survival between UGT1A1 poor metaboliser patients receiving a 30% upfront irinotecan dose reduction and fully-dosed intermediate and normal metabolisers.

**Implications of all the available evidence**

This real-world survival analysis in patients who received *UGT1A1* genotype-guided irinotecan dosing, supported by existing evidence, confirms the value of upfront *UGT1A1*-genotyping, followed by an upfront 30% dose-reduction in poor metabolisers. So far, the lack of previous reports on the impact on survival outcomes has hindered universal implementation of *UGT1A1* genotype-guided dosing of irinotecan in routine clinical practice. The present study now provides evidence that this strategy can be confidently applied to improve patient safety while preserving treatment effectiveness in poor metabolisers.

## Introduction

*UGT1A1* gene variations are associated with severe irinotecan-related toxicities, including diarrhoea and febrile neutropenia, which can result in treatment discontinuation, hospitalisation, reduced quality-of-life, and in some cases, death.^1^^,^^2^ SN-38, the active metabolite of irinotecan, is primarily inactivated by the enzyme uridine diphosphate-glucuronosyltransferase 1A1 (UGT1A1).^3^^,^^4^ Genetic variants in the *UGT1A1* gene, encoding UGT1A1, can cause reduced UGT1A1 activity and thereby SN-38 overexposure at standard irinotecan dosages.^5^ Homozygous or compound heterozygous carriers of relevant *UGT1A1* genetic variants (i.e. *UGT1A1*∗6, *UGT1A1*∗28, *UGT1A1*∗37, *UGT1A1*∗80, and *UGT1A1*∗93) are characterised as poor metabolisers (PM) and are at a two- to six-fold increased risk of severe irinotecan-related toxicity compared to heterozygous carriers or wild-type patients, who are characterised as intermediate metabolisers (IM) or normal metabolisers (NM), respectively.^6^, ^7^, ^8^, ^9^ UGT1A1 PM status is prevalent in approximately 10% of individuals in European populations, with higher frequencies observed in Black and East-Asian populations (up to 20%).^10^^,^^11^

Previously, several phase I dose-finding studies in UGT1A1 PMs suggested a 17–58% dose reduction of irinotecan would be necessary to normalise SN-38 levels and maintain an acceptable safety profile, but no specific optimal dose was established.^12^, ^13^, ^14^, ^15^, ^16^, ^17^, ^18^ Based on the pharmacokinetic data of these studies, a prospective trial assessed whether an upfront 30% dose reduction in UGT1A1 PMs would be sufficient to reduce severe toxicity risk in PMs. It demonstrated that an initial 30% irinotecan dose reduction in UGT1A1 PMs significantly reduced the incidence of febrile neutropenia and toxicity-related hospitalisation compared to historical fully-dosed PMs, while resulting in comparable severe toxicity rates between dose-reduced PMs and fully-dosed IM/NMs. Additionally, SN-38 exposure in dose-reduced PMs was still 32% higher compared to fully-dosed IM/NMs, suggesting treatment effectiveness was preserved in 30% dose-reduced PMs.^19^ Consequently, *UGT1A1* genotype-guided dosing of irinotecan, including a 30% dose reduction for PMs, has been recommended by the Dutch Pharmacogenetics Working Group (DPWG) since 2022 and has subsequently been implemented in routine clinical practice in multiple hospitals in the Netherlands to improve individual patient safety.^18^

Nonetheless, upfront screening for *UGT1A1* variants combined with dose reductions in UGT1A1 PMs is not yet universally adopted. A key concern is the potential compromise in anti-neoplastic efficacy when reducing irinotecan dose in PMs, as the impact of a reduced starting dose on progression-free survival (PFS) and overall survival (OS) remains unknown.^20^^,^^21^ Ideally, these outcomes would be studied in a randomised controlled trial (RCT) comparing reduced-dose and full-dose irinotecan in PMs. Yet, given the unacceptable risk of severe irinotecan-related toxicity in fully-dosed PMs,^6^^,^^7^ and the recommendation from irinotecan drug labels to consider lowering starting doses in PMs, a RCT is no longer considered ethically justifiable.^20^^,^^22^, ^23^, ^24^, ^25^ A more appropriate alternative is a longitudinal real-world survival analysis comparing survival of dose-reduced PMs with fully-dosed IM/NMs. Six meta-analyses, including four previously summarised in a systematic literature review by the DPWG, have reported no differences in response and survival outcomes between fully-dosed PMs and IM/NMs.^18^^,^^26^^,^^27^ As such, fully-dosed IM/NMs are a valid real-world comparator for assessing survival in dose-reduced PMs. To date, only small cohort studies have shown similar systemic SN-38 exposures and tumour response rates in dose-reduced PMs and in fully-dosed IM/NMs.^14^^,^^19^^,^^28^^,^^29^

Therefore, the aim of this study was to evaluate whether an upfront 30% initial dose reduction of irinotecan in UGT1A1 PMs affects survival by comparing PFS and OS between dose-reduced PMs and fully-dosed IM/NMs in a large real-world cohort.

## Methods

### Study design and patients

This retrospective, multicentre cohort study was conducted in six hospitals in the Netherlands (Catharina Hospital Eindhoven, Erasmus University Medical Centre Rotterdam, Leiden University Medical Centre, Jeroen Bosch Hospital ‘s Hertogenbosch, Maasstad Hospital Rotterdam, and Reinier de Graaf Gasthuis Hospital Delft). The study population consisted of a consecutive cohort of adult patients (≥18 years) who received *UGT1A1* genotype-guided irinotecan dosing for either colorectal cancer or pancreatic cancer between August 2017 and April 2024. All currently registered irinotecan-containing regimens for the treatment of colorectal or pancreatic cancer were included, except for treatment with intraperitoneal or liposomal irinotecan. *UGT1A1* genotype-guided dosing was either performed as part of the study by Hulshof et al. (August 2017–December 2020),^16^ or as part of routine clinical care (December 2020–April 2024).

### Procedures

All patients were genotyped for *UGT1A1*∗28 at local ISO15189 certified laboratories. Testing for additional *UGT1A1* variants (*UGT1A1*∗6, *UGT1A1*∗36, *UGT1A1*∗37, *UGT1A1*∗80, and *UGT1A1*∗93) varied across hospitals and time periods, depending on updated guidelines (Supplementary Methods). Genotypes were classified into UGT1A1 PM, IM, or NM groups and irinotecan dose was administered according to the DPWG guideline.^18^ In short, homozygous carriers or compound heterozygous carriers of relevant *UGT1A1* variants were defined as UGT1A1 PM, heterozygous carriers were defined as UGT1A1 IM, and wildtype individuals were defined as UGT1A1 NM (Supplementary Methods). PMs received an initial dose reduction of 30%, while IMs and NMs received the standard full irinotecan dose. Further dose individualisation in later treatment cycles based on neutrophilic count and clinical tolerability for both PMs and IM/NMs was carried out at the discretion of the treating physician. Patients receiving combination treatment with fluoropyrimidines were additionally genotyped for *DPYD* variants and dosed according to the respective DPWG guidelines.

Clinical assessments and radiological imaging were periodically performed and reported in electronic health records (EHR) as part of routine clinical care in all hospitals according to centre-specific standard treatment protocols. Patients receiving (neo)adjuvant treatment were assessed by their physician prior to each cycle. Upon treatment completion they were typically seen every 3 months at the outpatient clinic, with radiological imaging generally performed every three to four months. Those undergoing palliative chemotherapy were evaluated by their physician before each cycle, with radiological imaging generally performed every third to fourth cycle. Patients were followed up until death or until data cut-off.

Data collection took place between September 2024 and April 2025 and all data were collected retrospectively from EHRs, which contain well-structured, routinely recorded information by health specialists on the demographics, administered chemotherapy regimens and dosages, laboratory values, in- and out-patient visits, and radiological reports of each patient. More specifically, both the administered irinotecan dosage and *UGT1A1* genotyping results were thoroughly documented in the EHR, with verification steps by laboratory technicians, oncologists, pharmacists, and nurses as part of routine care, ensuring data accuracy. Data for each patient were manually collected by one data collector, and verified by a second data collector. In case of disagreement, a third data collector reviewed the records and resolved discrepancies. All data were entered into an electronic case report form (eCRF) in Research Manager, with quality checks and validation processes implemented to ensure data accuracy. Standard operating procedures were followed for consistent and reliable data collection by all data collectors.

### Outcomes

The primary objective of this study was to assess the impact of an initial 30% dose reduction of irinotecan in UGT1A1 PMs on survival compared to fully-dosed UGT1A1 IM/NMs. The primary endpoint was PFS. PFS was defined as the time between initiation of irinotecan treatment and the first signs of disease progression by either clinical signs or radiological imaging (in accordance with RECIST 1.1), or death from any cause, whichever came first. Disease recurrence was considered a progression event in patients who received (neo)adjuvant irinotecan. The secondary endpoint was OS, which was defined as the time between initiation of irinotecan treatment and death from any cause. Patients who did not experience disease progression or death before the end of follow-up were censored at the last date known to be alive.

Baseline characteristics that were considered relevant for treatment outcomes were collected as well. These included sex assigned at birth, age, ethnicity, world health organization (WHO) performance status, smoking status, primary tumour type, stage of cancer, treatment regimen, and previous anticancer treatment. Irinotecan dosages and dose modifications were collected for the first ten cycles. Relative dose intensity (RDI) was calculated by dividing the administered dose (mg/m^2^) in each cycle by the standard dose for UGT1A1 IM/NM patients (mg/m^2^) × 100%, considering the indication and treatment regimen that was applicable for each patient. Dose modifications were defined as a reduction or escalation of >10% of the RDI in comparison to the previous treatment cycle, thereby excluding dose changes attributable solely to variations in body surface area or dose rounding during infusion preparation. Additionally, data on overall irinotecan-related severe toxicity, defined as grade ≥3 neutropenia, febrile neutropenia, diarrhoea, and/or toxicity-related hospitalisation, were collected for the first three cycles and graded according to the National Cancer Institute's Common Terminology Criteria for Adverse Events (NCI-CTCAE), version 5.0.

### Statistical analysis

To assess the impact of an actual 30% irinotecan dose reduction in PMs on survival, patients were included in the primary analysis if irinotecan was dosed according to *UGT1A1* genotype in at least cycle 1 (i.e. an initial 100% dose intensity for IM/NMs and an initial 70% dose intensity for PMs; ±10% deviation allowed). Additionally, a secondary analysis was conducted in all consecutively genotyped patients treated with irinotecan regardless of their irinotecan dose in cycle 1.

PFS and OS survival curves were generated using the Kaplan–Meier method. Median PFS and OS were compared between PMs and IM/NMs using Kaplan–Meier estimates and a stratified log-rank test for equality of survival curves (stratified by tumour type). The reverse Kaplan–Meier method was used to calculate median follow-up and to compare censoring patterns between PM and IM/NM groups. Univariable Cox regression analyses were conducted to evaluate the association between UGT1A1 groups (PM *vs* IM/NM) and endpoints PFS and OS. Associations between relevant covariates and PFS and OS were also assessed using univariable Cox regressions. Multivariable Cox regression analyses were performed for PM *vs* IM/NM groups, adjusted for covariates with *P* < 0.10 in univariable Cox regression analyses. Hazard ratios (HRs) and their corresponding 95% confidence intervals (CI) were calculated. All Cox regression analyses were conducted as stratified Cox regressions by tumour type (i.e. colorectal cancer and pancreatic cancer). This stratified approach allows for estimation of the overall effect of PM *vs* IM/NM on survival while also controlling for inherent differences in the risk of event (i.e. disease progression or death) specific to each tumour type, by allowing each tumour type subgroup to have its own baseline hazard. A complete-case analysis was performed for the multivariable Cox regression analyses under the missing at random assumption (see also Statistical Analysis Plan in Supplementary Material).

Ideally, PFS and OS would be analysed separately by tumour type, treatment setting and treatment regimen. However, this approach was not pursued in the primary analysis, as we expected to include a heterogenic patient population with small subgroup sizes, thereby impairing adequate assessment of the PMs *vs* IM/NMs effect. Therefore, in the primary analysis, we stratified by tumour type and adjusted for additional covariates, including stage and treatment regimen, using multivariable Cox regression analysis. Consequently, the reported Kaplan–Meier PFS and OS estimates should be interpreted in terms of the relative difference between PMs and IM/NMs, rather than their absolute values, since they reflect outcomes of a diverse group of patients. As a secondary analysis, we conducted a survival analysis using an alternative stratification method including treatment setting (curative/palliative) and tumour type, and assessed whether the PMs *vs* IM/NMs effect was consistent across strata (curative colorectal cancer, curative pancreatic cancer, palliative colorectal cancer, and palliative pancreatic cancer). Additionally, subgroup survival analyses were conducted comparing PMs and IM/NMs with pancreatic cancer, treated with standard FOLFIRINOX (containing 180 mg/m^2^ irinotecan) and modified FOLFIRINOX (containing 150 mg/m^2^ irinotecan).

Furthermore, a formal non-inferiority analysis was initially considered in the design of the study, but pre-analysis power calculations—based on the expected maximum available sample size and event rate—indicated that a HR non-inferiority margin of 1.40 for PFS and 1.50 for OS were the lowest margins achievable with 80% power (see Statistical Analysis Plan in Supplementary Material). Since these non-inferiority margins were considered too large to justify clinical non-inferiority, we instead opted to compare PFS and OS outcomes between PMs and IM/NMs descriptively.

Patient and treatment characteristics were compared between UGT1A1 groups (PM *vs* IM/NM) using descriptive statistics. For the toxicity analysis, Chi-square Test or Fisher's Exact Test was used to compare frequencies of overall severe (CTCAE grade ≥3) toxicity between UGT1A1 groups (PM *vs* IM/NM).

For all analyses, *P* < 0.05 was considered statistically significant. All statistical analyses were performed using SPSS for Windows (IBM SPSS statistics, Armonk, New York, USA; version 29.0.0.0). The production of plots was performed using GraphPad Prism for Windows (GraphPad Software, San Diego, California, USA; version 10.2.3).

Additional details of the study methods, including power calculation, secondary analyses, and subgroup analyses are provided in the Statistical Analysis Plan and Supplementary Methods in the Supplementary Material.

### Ethical approval

This study was conducted in accordance with the Declaration of Helsinki and the ICH-GCP guidelines. The Medical research Ethics Committee United (MEC-U, Nieuwegein, the Netherlands) approved the study protocol and declared the study not to be subject to the Dutch Medical Research Involving Human Subjects Act (MEC-U study registration number W24.109). Local approval was obtained from the institutional review board (IRB) or ethics committee of each participating hospital and informed consent was waived for patients from routine clinical care owing to the retrospective nature of the study. Patients who had registered an objection to the use of their data for research in the EHR were excluded.

### Role of the funding source

This research received no specific grant from any funding agency in the public, commercial, or not-for-profit sectors.

## Results

**Fig. 1:**
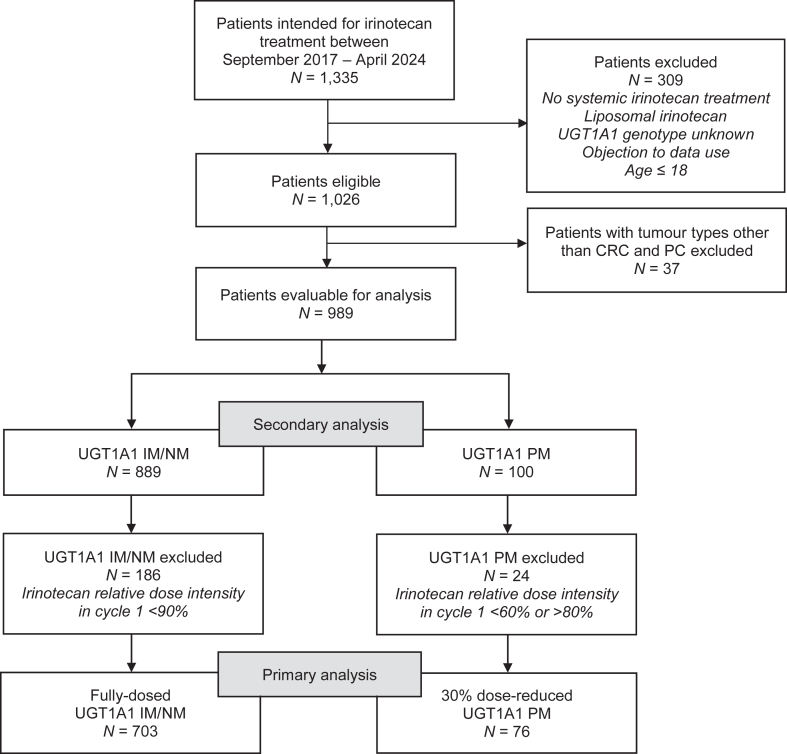
**Patient inclusion flow diagram**. CRC, colorectal cancer; IM, intermediate metaboliser; NM, normal metaboliser; N, number of patients; PC, pancreatic cancer; PM, poor metaboliser; UGT1A1, Uridine Diphosphate Glucuronosyltransferase 1A1.

In total, 1335 patients intended for treatment with irinotecan were screened for eligibility. Of these, 346 patients were excluded due to non-eligibility (i.e. not having actually received systemic irinotecan treatment, age ≤18, a registered objection to data use, tumour types other than colorectal or pancreatic cancer, or not having been genotyped for *UGT1A1*). Additionally, 210 patients were excluded from the primary analysis because of having received a cycle 1 irinotecan dosage that was not concordant with their *UGT1A1* genotype. Overall, these patients were of older age and had poorer WHO performance status (Supplementary Table S1). The remaining 779 patients were included in the primary analysis (Fig. 1). Of these, 396 patients had pancreatic cancer, including 41 PMs, and 383 patients had colorectal cancer, including 35 PMs.

Genotypes were in Hardy–Weinberg equilibrium (Supplementary Table S2 and Supplementary Fig. S1). Patient demographics and baseline characteristics were well-balanced across PM and IM/NM groups (Table 1, Supplementary Table S3).

**Table 1:** Baseline characteristics of patients in the primary analysis.

	UGT1A1 PM *N* = 76	UGT1A1 IM/NM *N* = 703	All patients *N* = 779
Age in years, median (IQR)	62 (55–69)	62 (54–68)	62 (55–68)
Sex, *N* (%)			
Male	41 (53.9)	391 (55.6)	432 (55.5)
Female	35 (46.1)	312 (44.4)	347 (44.5)
Ethnic origin, *N* (%)			
European	66 (86.8)	649 (92.3)	715 (91.8)
Middle-Eastern	3 (3.9)	16 (2.3)	19 (2.4)
North-African	0 (0.0)	10 (1.4)	10 (1.3)
Sub-Saharan African	0 (0.0)	5 (0.7)	5 (0.6)
Asian	1 (1.3)	5 (0.7)	6 (0.8)
Hispanic	4 (5.3)	13 (1.8)	17 (2.2)
Other	1 (1.3)	1 (0.1)	2 (0.3)
Unknown	1 (1.3)	4 (0.6)	5 (0.6)
BSA in m^2^, median (IQR)	1.85 (1.72–2.03)	1.92 (1.77–2.06)	1.91 (1.76–2.06)
WHO performance, *N* (%)			
0–1	72 (94.7)	672 (95.6)	744 (95.5)
2–3	4 (5.3)	31 (4.4)	35 (4.5)
Smoking status, *N* (%)			
Never	30 (39.5)	275 (39.1)	305 (39.2)
Smoker	12 (15.8)	106 (15.1)	118 (15.1)
Ex-smoker	32 (42.1)	295 (42.0)	327 (42.0)
Unknown	2 (2.6)	27 (3.8)	29 (3.7)
Tumour type, *N* (%)			
Colorectal cancer	35 (46.1)	348 (49.5)	383 (49.2)
Pancreatic cancer	41 (53.9)	355 (50.5)	396 (50.8)
Tumour stage, *N* (%)			
Stage I	1 (1.3)	40 (5.7)	41 (5.3)
Stage II	12 (15.8)	84 (11.9)	96 (12.3)
Stage III	17 (22.4)	173 (24.6)	190 (24.4)
Stage IV	46 (60.5)	406 (57.8)	452 (58.0)
Treatment regimen + standard irinotecan dosage, *N* (%)			
FOLFIRINOX (180 mg/m^2^ q2w)	32 (42.1)	244 (34.7)	276 (35.4)
mFOLFIRINOX (150 mg/m^2^ q2w)	9 (11.8)	112 (15.9)	121 (15.5)
Irinotecan monotherapy—low dose^a^	3 (3.9)	36 (5.1)	39 (5.0)
Irinotecan monotherapy—high dose^b^	6 (7.9)	39 (5.5)	45 (5.8)
FOLFIRI (180 mg/m^2^ q2w)	10 (13.2)	97 (13.8)	107 (13.7)
FOLFIRI + bevacizumab/cetuximab (180 mg/m^2^ q2w)	4 (5.3)	52 (7.4)	56 (7.2)
FOLFOXIRI (165 mg/m^2^ q2w)	1 (1.3)	43 (6.1)	44 (5.6)
FOLFOXIRI + bevacizumab (165 mg/m^2^ q2w)	7 (9.2)	53 (7.5)	60 (7.7)
Other^c^	4 (5.3)	27 (3.8)	31 (4.0)
Previous surgery, *N* (%)			
Yes	29 (38.2)	289 (41.1)	318 (40.8)
No	47 (61.8)	414 (58.9)	461 (59.2)
Previous radiotherapy, *N* (%)			
Yes	18 (23.7)	128 (18.2)	146 (18.7)
No	58 (76.3)	575 (81.8)	633 (81.3)
Previous chemotherapy, *N* (%)			
Yes	31 (40.8)	297 (42.2)	328 (42.1)
No	45 (59.2)	406 (57.8)	451 (57.9)
Previous number of lines, *N* (%)			
0	55 (72.4)	521 (74.1)	576 (73.9)
1	19 (25.0)	156 (22.2)	175 (22.5)
2	2 (2.6)	21 (3.0)	23 (3.0)
3	0 (0.0)	5 (0.7)	5 (0.6)
Previous treatment with irinotecan, *N* (%)			
Yes	3 (3.9)	12 (1.7)	15 (1.9)
No	73 (96.1)	691 (98.3)	764 (98.1)

*BSA*, Body Surface Area; *IM*, intermediate metaboliser; *IQR*, interquartile range; *NM*, normal metaboliser; *N*, number of patients; *PM*, poor metaboliser; *UGT1A1*, Uridine Diphosphate Glucuronosyltransferase 1A1; *WHO*, World Health Organisation.

a Irinotecan monotherapy low dose: 180 mg/m^2^ or 450 mg flat dose either q2w or q3w.

b Irinotecan monotherapy high dose: 350 mg/m^2^ or 600 mg flat dose either q2w or q3w.

c Other treatment regimens and respective standard irinotecan dosages included: irinotecan monotherapy 210 mg/m^2^ (*N* = 10), irinotecan + panitumumab 180 mg/m^2^ q2w (*N* = 8), FOLFIRI + panitumumab 180 mg/m^2^ q2w (*N* = 7), FOLFIRI 180 mg/m^2^ q3w (*N* = 1), FOLFIRI + ramucirumab 180 mg/m^2^ q2w (*N* = 1), mFOLFOXIRI + panitumumab 150 mg/m^2^ q2w (*N* = 1), irinotecan + cetuximab 150 mg/m^2^ q2w (*N* = 1), irinotecan + bevacizumab 180 mg/m^2^ (*N* = 1), irinotecan monotherapy 240 mg flat q2w (*N* = 1).

In the primary analysis, 649 (83.3%) PFS events and 527 (67.7%) OS events had occurred by study end, indicating mature PFS and OS data. Median follow-up was 23.4 months (95% CI 15.2–31.6) in the PM group and 28.8 months (95% CI 25.8–31.8) in the IM/NM group. Censoring patterns were well-balanced across PM and IM/NM groups. No violation of the proportional hazards assumption was found for any of the Cox regressions.

**Fig. 2:**
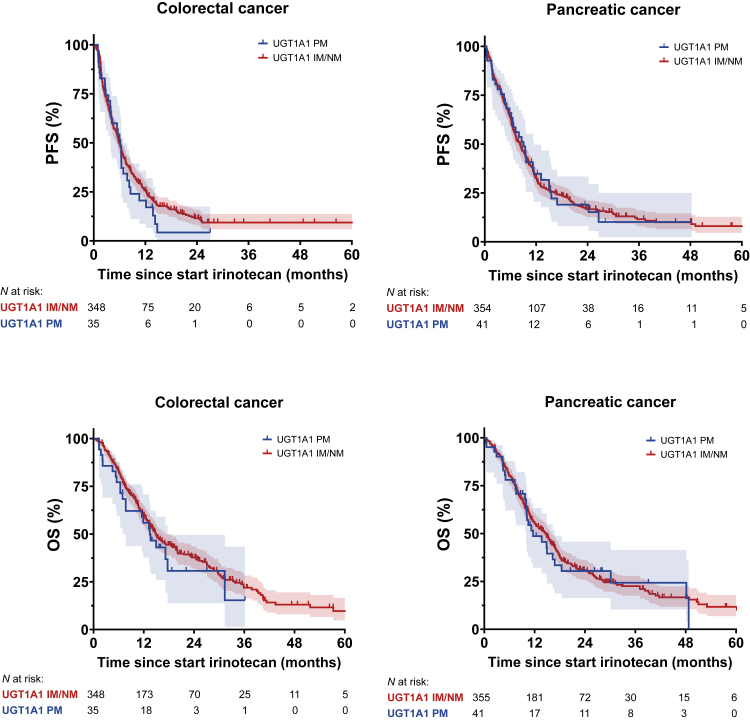
**Kaplan–Meier plots for PFS and OS of UGT1A1 PM patients treated with a reduced irinotecan dose and IM/NM patients treated with a full irinotecan dose for colorectal and pancreatic cancer in the primary analysis**. Censoring is indicated by tick marks. The shaded area represents the 95% CI. A stratified log-rank test (stratified by tumour type) for comparing Kaplan–Meier curves between PM and IM/NM showed a *P* value of *P* = 0.54 for PFS and *P* = 0.42 for OS. CI, confidence interval; IM, intermediate metaboliser; NM, normal metaboliser; N, number of patients; OS, overall survival; PM, poor metaboliser; PFS, progression-free survival; UGT1A1, Uridine Diphosphate Glucuronosyltransferase 1A1.

Kaplan–Meier estimates indicated no significant differences in PFS between dose-reduced PM and fully-dosed IM/NM patients with pancreatic cancer or colorectal cancer (stratified log-rank test PMs *vs* IM/NMs: *P* = 0.54) (Fig. 2, Table 2). UGT1A1 group (PMs *vs* IM/NMs) was not associated with PFS in stratified univariable Cox regression analysis (Table 2). Stratified multivariable Cox regression analysis showed no statistically significant difference in PFS for dose-reduced PMs compared to fully-dosed IM/NMs (adjusted HR = 1.02; 95% CI 0.78–1.32; *P* = 0.90) (Table 2, Supplementary Table S4).

**Table 2:** PFS and OS with corresponding HRs in UGT1A1 PM patients with a reduced irinotecan dose vs IM/NM patients with a full irinotecan dose in the primary analysis.

	*N*^a^	PFS events, *N* (%)	Median PFS, months	95% CI, months	1-Year PFS, %	95% CI, %	*N*	Deceased, *N* (%)	Median OS, months	95% CI, months	1-Year OS, %	95% CI, %
*Colorectal*												
IM/NM	348	291 (83.6)	6.0	5.3–6.7	25.3	20.6–30.0	348	222 (63.8)	14.9	12.9–17.0	59.8	54.5–65.1
PM	35	31 (88.6)	6.2	5.1–7.3	17.1	4.0–30.2	35	22 (62.9)	13.4	9.4–17.5	55.9	39.0–72.8
*Pancreatic*												
IM/NM	354	294 (83.1)	8.3	7.2–9.4	32.5	27.6–37.4	355	254 (71.5)	14.8	12.7–16.8	55.4	50.1–60.7
PM	41	33 (80.5)	9.0	6.2–11.8	34.8	19.7–49.9	41	29 (70.7)	11.6	6.6–16.7	48.7	32.8–64.6

	*N*^a^	PFS events, *N* (%)	Univariable analysis PFS^b^		Multivariable analysis PFS^c^		*N*	Deceased, *N* (%)	Univariable analysis OS^b^		Multivariable analysis OS^d^	
			HR (*95% CI*)	*P* value	HR (*95% CI*)	*P* value			HR (*95% CI*)	*P* value	HR (*95% CI*)	*P* value
*All patients*												
IM/NM (Ref)	702	585 (83.3)	–	–	–	–	703	476 (67.8)	–	–	–	–
PM	76	64 (84.2)	1.08 *(0.84–1.40)*	0.54	1.02 *(0.78–1.32)*	0.90	76	51 (67.1)	1.13 *(0.84–1.51)*	0.42	1.10 *(0.82–1.48)*	0.51

*CI*, confidence interval; *HR*, hazard ratio; *IM*, intermediate metaboliser; *NM*, normal metaboliser; *N*, number of patients; *OS*, overall survival; *PM*, poor metaboliser; *PFS*, progression-free survival; *Ref*, reference group.

a One IM/NM patient was excluded from PFS analysis because of an unknown disease progression date.

b Univariable Cox regression analysis stratified by tumour type.

c Multivariable Cox regression PFS analysis stratified by tumour type and adjusted for covariates age, WHO performance status, ethnicity, tumour stage, treatment regimen, previous radiotherapy, previous chemotherapy, and previous number of chemotherapy lines.

d Multivariable Cox regression OS analysis stratified by tumour type and adjusted for covariates age, WHO performance status, tumour stage, treatment regimen, previous surgery, previous radiotherapy, previous chemotherapy, and previous number of chemotherapy lines.

Similarly, OS was not significantly different between dose-reduced PMs and fully-dosed IM/NMs (stratified-log rank test PMs *vs* IM/NMs: *P* = 0.42) (Fig. 2, Table 2). UGT1A1 group (PMs *vs* IM/NMs) was not associated with OS in stratified univariable Cox regression analysis. Also, in stratified multivariable Cox regression analysis no statistically significant difference in OS was found for dose-reduced PMs compared to fully-dosed IM/NMs (adjusted HR = 1.10; 95% CI 0.82–1.48; *P* = 0.51) (Table 2, Supplementary Table S4).

A secondary analysis was conducted in all genotyped patients treated with systemic irinotecan, irrespective of administered dose in cycle 1, consisting of 889 IM/NMs and 100 PMs (Supplementary Table S5). This included 186 IM/NMs and 15 PMs with a reduced irinotecan starting dose due to frailty or toxicity of previous treatments, and nine PMs who unintendedly received a full irinotecan dose, who were all excluded from the primary analysis. The results of this secondary analysis were consistent with those of the primary analysis, with HRs for PFS and OS (PMs *vs* IM/NMs) close to 1.0 (PFS: adjusted HR = 0.95; 95% CI 0.71–1.27; *P* = 0.74; OS: adjusted HR = 0.84; 95% CI 0.61–1.17; *P* = 0.30) (Supplementary Fig. S2, Supplementary Tables S6–S8). In another secondary analysis, further stratification for treatment setting (curative *vs* palliative) in addition to tumour type yielded similar results as the primary analysis (PMs *vs* IM/NMs: DFS/PFS: adjusted HR = 1.02; 95% CI 0.79–1.33; *P* = 0.91; OS: adjusted HR = 1.13; 95% CI 0.84–1.51; *P* = 0.43) (Supplementary Figs. S3 and S4 and Supplementary Tables S9–S11). The individual HRs of PM *vs* IM/NM were directionally consistent across tumour types and treatment settings, showing no statistically significant differences in DFS, PFS, or OS between dose-reduced PMs and fully-dosed IM/NMs in any of the strata (Supplementary Table S12). Furthermore, there were no statistically significant differences in PFS or OS between dose-reduced PMs and fully-dosed IM/NMs in standard FOLFIRINOX and modified FOLFIRINOX subgroups (Supplementary Fig. S5 and Supplementary Tables S13 and S14). Lastly, no statistically significant differences in PFS and OS were found between IM and NM patients (IM *vs* NM; PFS: adjusted HR = 1.01; 95% CI 0.86–1.19; *P* = 0.89; OS: adjusted HR = 1.04; 95% CI 0.87–1.25; *P* = 0.69; complete data not shown).

Safety data and irinotecan dose modifications for both PM and IM/NM groups are summarised in Table 3, Supplementary Table S8, and Supplementary Table S15. Overall, *UGT1A1* genotype-guided dosing resulted in the occurrence of severe toxicity in 23 of 76 PMs (30.3%) and 234 of 703 IM/NMs (33.3%) during the first three treatment cycles (*P* = 0.59) (Table 3). IM/NMs were more frequently dose-reduced after the first irinotecan cycle compared to PMs (48.2% *vs* 34.3%; *P* = 0.03). Six PMs with an upfront 30% dose reduction underwent dose titration in subsequent cycles, but only two tolerated treatment on the escalated irinotecan dose. Median RDI of irinotecan remained consistent throughout treatment cycles for both PMs and IM/NMs (Table 3).

**Table 3:** Toxicity and irinotecan dose modifications of UGT1A1 PM patients with a reduced irinotecan dose and IM/NM patients with a full irinotecan dose in the primary analysis.

	UGT1A1 PM *N* = 76	UGT1A1 IM/NM *N* = 703	*P* value^f^
**Overall grade ≥ 3 toxicity**^a^**in the first three cycles, *N* (%)**	23 (30.3)	234 (33.3)	0.59
**Dose modification(s)**^b^^,^^c^**of irinotecan after the first cycle, *N* (%)**			0.05
No dose modification	43 (56.6)	351 (49.9)	
≥1 dose modification(s)	27 (35.5)	328 (46.7)	
Not applicable^d^	6 (7.9)	24 (3.4)	
**Dose reduction(s)**^c^**of irinotecan after the first cycle, *N* (%)**^e^	24 (34.3)	327 (48.2)	0.03
**Dose escalation(s)**^c^**of irinotecan after the first cycle, *N* (%)**^e^	6 (8.6)	12 (1.8)	0.004
**Relative dose intensity of irinotecan in %, median (IQR)**			
Relative dose intensity first cycle	70 (68–71)	100 (98–101)	<0.001
Relative dose intensity last cycle	68 (61–71)	94 (75–100)	<0.001
Average relative dose intensity all cycles	69 (64–71)	96 (84–100)	<0.001

*IM*, intermediate metaboliser; *IQR*, interquartile range; *NM*, normal metaboliser; *N*, number of patients; *PM*, poor metaboliser; *UGT1A1*, Uridine Diphosphate Glucuronosyltransferase 1A1.

a Overall severe toxicity included one of more of the following toxicities: Grade ≥3 febrile neutropenia, Grade ≥3 neutropenia, Grade ≥3 diarrhoea, irinotecan-related hospitalisation.

b Dose reduction and/or dose escalation.

c Dose reductions or escalations were defined as a change in ≥10% of irinotecan dose compared to the previous cycle.

d Patients that only received one treatment cycle with irinotecan.

e Numbers and percentages were determined only for patients that received ≥2 irinotecan treatment cycles (PM, *N* = 70; IM/NM, *N* = 679).

f *P* value (two-sided) comparing UGT1A1 PM with UGT1A1 IM/NM. For categorical variables Chi-Square or Fisher's Exact Test (in case cells had a ≥20% expected count less than 5) was used. For relative dose intensity Mann–Whitney U test was used.

## Discussion

This is the largest study to date to report survival data of patients who received *UGT1A1* genotype-guided dosing of irinotecan. Overall, the results from our seven-year longitudinal study show that the survival of PM patients who received an initial 30% dose reduction is not significantly different from the survival of fully-dosed IM/NM patients. The prevalence of UGT1A1 PMs in our study population was 10.1%, highlighting the relevance of this patient group in routine clinical care.

In our primary analysis, HRs for PFS and OS were close to 1, with narrow 95% CIs (upper bound of 1.32 for PFS and 1.48 for OS) and non-significant *P* values, suggesting that no relevant differences exist in either PFS or OS between 30% dose-reduced PM and fully-dosed IM/NMs. When including all genotyped patients with systemic irinotecan, irrespective of dose in cycle 1, upper 95% CI bounds were below 1.30 for PFS and below 1.20 for OS. Moreover, additional stratification by treatment setting revealed no significant differences in DFS, PFS and OS rates between 30% dose-reduced PMs and fully-dosed IM/NMs in all subgroups (curative colorectal cancer, curative pancreatic cancer, palliative colorectal cancer, and palliative pancreatic cancer). Further subgroup analyses reinforced the robustness of these results, consistently showing no clinically meaningful differences between 30% dose-reduced PMs and fully-dosed IM/NMs across various tumour types, treatment settings, and treatment regimens. The findings indicate that *UGT1A1* genotype-guided dosing does not result in statistically significant differences in survival between PMs and IM/NMs, which is in line with prior, smaller studies in which *UGT1A1* genotype-guided dose individualisation did not appear to compromise treatment effectiveness in PMs compared to IM/NMs.^23^^,^^28^^,^^30^ For instance, data from the PREPARE trial showed comparable three-year overall survival between four PMs treated with a 30% reduced initial irinotecan dose and 42 IM/NMs receiving the full dose.^23^ Additionally, our results are supported by pharmacokinetic data that demonstrated that systemic exposure to the bioactive metabolite SN-38 was still 32% higher in 30% dose-reduced PMs than the exposure found in IM/NMs treated with a full dose.^19^ To summarise, this study's findings corroborate existing literature and confirm that *UGT1A1* genotype-guided irinotecan dose adjustments correct for SN-38 overexposure in PMs without compromising treatment efficacy.

This study supports the ongoing shift away from the classical treatment paradigm that higher chemotherapeutic dosages result in better survival outcomes. Full irinotecan dosages in PMs are not associated with improved survival outcomes, but result in unacceptably high toxicity rates.^1^^,^^2^^,^^19^ By reducing the initial irinotecan dose in PMs, severe toxicity can be avoided, preventing unnecessary dose reductions, treatment interruptions, and premature treatment discontinuation in later cycles.^19^^,^^31^ In our study, the incidence of overall severe irinotecan-related toxicity of dose-reduced PMs (30.3%) was comparable with fully-dosed IM/NMs (33.3%). It was also markedly lower than the incidence reported previously for a historical cohort of fully-dosed PMs (52%).^19^ Additionally, we found that the majority of PMs who received a 30% upfront dose reduction required no further dose reductions in subsequent cycles. These results demonstrate an enormous improvement in treatment tolerability for UGT1A1 PMs, reaffirming the conclusion of our previous study^19^ that an initial 30% dose reduction in UGT1A1 PMs is adequate to reduce the risk of severe irinotecan-related toxicity to an acceptable level. The higher observed frequency of toxicity-related dose reductions in IM/NMs compared to PMs suggests that IM/NM patients may benefit from further treatment optimisation. Alternatively, this finding could suggest that clinicians may be more cautious about reducing irinotecan doses with more than 30% in PMs due to concerns about efficacy, despite the occurrence of toxicity. We believe that further individualisation of irinotecan dosage after the first treatment cycle based on clinical tolerability remains important for both PMs and IM/NMs. It is also worth highlighting that dose escalations of irinotecan remain an option for UGT1A1 PMs who have demonstrated good tolerability to the initial cycles with reduced irinotecan doses.^18^ While this was attempted in six PMs in our study, only two tolerated treatment on the escalated dose.

This study has several strengths and limitations. First, we opted to perform an observational study as a RCT is considered unethical. Although our data were retrospectively collected, the endpoints (PFS and OS) are major events with clinical consequences and were therefore well-documented in EHRs. Similarly, *UGT1A1* genotype and irinotecan dosage are routinely recorded and verified in the EHR of each patient as part of standard care, ensuring accurate availability for collection in this study. Second, we were unable to conduct a formal non-inferiority analysis with a clinically acceptable non-inferiority margin. Sample-size calculations imply that at least 336 PM patients and an event rate of 80% are required to formally assess non-inferiority with a more stringent non-inferiority margin of 1.20 and a power of 80%, meaning such an analysis is not feasible for the time-being. Although we were unable to prove non-inferiority of dose-reduced PMs compared to fully-dosed IM/NMs, the findings of the present study offer the best available evidence to date regarding the potential impact of *UGT1A1*-guided dose reductions on treatment effectiveness. Third, we included a very heterogenic patient population, but we were able to statistically adjust in our analyses for known relevant covariates, *e.g.* stage, regimen, tumour type and treatment history. Additionally, a major strength of this study is that we conducted several relevant secondary and subgroup analyses, including an analysis stratified per tumour type and treatment setting. While these analyses were exploratory and consisted of small subgroup sizes, they consistently supported the conclusion that *UGT1A1* genotype-guided dosing does not impair treatment efficacy. Finally, aside from *UGT1A1*∗28, additional genotyping is currently recommended for two variants (∗6, ∗37) and optional for three other variants (∗36, ∗80 and ∗93),^11^^,^^18^ but not all patients in our study were genotyped for these additional *UGT1A1* variants. Additional genotyping of ∗6 and ∗37 might have identified more PM patients, who would then have received an upfront dose reduction, rather than receiving the full irinotecan dose as IM/NM patients did. Potential misclassification of PMs as IM/NMs may therefore cause conservative bias, meaning the impact of a dose reduction in PMs is underestimated. Although it cannot be fully ruled out that a few PM patients may have been misclassified as IM or NM, this is not expected to have affected our findings, given that our study cohort was predominantly of European ancestry (92%), in which the ∗6, and ∗37 variants are known to be rare.^14^^,^^15^ Nonetheless, we believe that *UGT1A1* testing panels should include the ∗6, ∗28, and ∗37 variants to ensure that PMs from diverse ethnic backgrounds are accurately detected. This is especially relevant for individuals from non-European populations, in which the ∗6 or ∗37 variants are generally more prevalent. Moreover, the ∗80 and ∗93 variants are in high linkage disequilibrium with the ∗28 variant, meaning there is substantial overlap in the *UGT1A1* test results for these variants.^10^^,^^11^ In our study population, variants ∗28 or ∗37 were detected either with or without ∗80 or ∗93, whereas ∗80 or ∗93 did not occur independently of the ∗28 or ∗37 variant. This pattern supports evidence that the variants ∗80 and ∗93 do not directly alter UGT1A1 phenotype, but rather act as tag single nucleotide polymorphisms due to their high linkage disequilibrium.^11^ Accordingly, we believe that genotyping of the ∗80 and ∗93 variants is redundant when the ∗28 or ∗37 variants are already included in the genotyping panel. While the ∗36 variant is rare in European populations, it has been associated with increased UGT1A1 enzyme activity,^32^ which could potentially reduce treatment effectiveness, but robust evidence and specified recommendations for this variant are currently lacking.^11^^,^^18^ We were unable to assess the impact of this variant on survival outcomes in the present study, as no ∗36 carriers were identified in our study population. Future studies should therefore address and elucidate the clinical impact of the ∗36 variant.

While we recognise the limitations of the present analysis, our study strongly suggests that a negative impact of an upfront 30% dose reduction in UGT1A1 PMs on survival is unlikely. Based on the available evidence, we consider it in the best interest of patients to use available upfront screening methods to prevent severe toxicity in UGT1A1 PMs by applying genotype-guided irinotecan dosing. Notably, several pharmacogenetic guidelines—DPWG, French National Network on Pharmacogenetics (RNPGx), and the Italian Society of Pharmacology and Italian Association of Medical Oncology (SIF-AIOM)—already recommend pre-treatment *UGT1A1*-genotyping and an upfront 30% dose reduction of irinotecan for UGT1A1 PMs, followed by up-titration if well-tolerated.^18^^,^^21^^,^^33^ Moreover, *UGT1A1*-genotyping before irinotecan administration is mentioned as an option in the drug labels for irinotecan by the European Medicine Agency and the U.S. Food and Drug Administration, and lower starting doses in known PMs are advised.^24^^,^^25^ Specified dosing recommendations by best practice guidelines from the European Society for Medical Oncology (ESMO) and the American Society for Clinical Oncology (ASCO) are currently lacking, but would greatly accelerate implementation in clinical practice.

In conclusion, the results of this real-world survival analysis provide reassuring evidence that an upfront 30% irinotecan dose reduction in UGT1A1 PMs does not lead to clinically or statistically significant differences in survival outcomes compared to fully-dosed IM/NM patients. While we did not perform a formal non-inferiority analysis, the present study indicates that *UGT1A1* genotype-guided dose individualisation of irinotecan can lower severe toxicity risk in PM patients without compromising treatment effectiveness.

## Contributors

Conceptualisation and methodology: SLJP, NH, SBo, RHNS, AMT, HG, HJG, RHJM, and MJD designed the study, with additional input from SBi, IEGH, BCMH, AJV, and MLW. Resources: SBi, IEGH, BCMH, AJV, MLW, RHNS, AMT, HG, HJG, RHJM, and MJD were involved in the patient inclusion and the provision of individual patient data. Investigation: SLJP, NH, DB, RG, ECH, FMM, MW, MJ, GO, QYW, and AO performed the individual patient data collection and had access to the study data. All authors contributed to data interpretation. Data curation: SLJP, NH, DB, RG, ECH, FMM, MW, MJ, GO, QYW, and AO. Formal analysis: SLJP and NH led the statistical analysis with support from SBo. Validation: SLJP, NH, and DB had full access to all the data and verified the data and the executed analyses. Visualisation: SLJP and NH created tables and figures. Project administration: SLJP, NH, and DB. Supervision: RHNS, AMT, HG, HJG, RHJM, and MJD. Writing–original draft: SLJP and NH wrote the first draft of the report with input from RHJM and MJD. Writing–review & editing: All authors were involved in the preparation of the report for publication, approved the final version of the manuscript, and took final responsibility for the decision to submit the manuscript for publication.

## Data sharing statement

The data that support the findings of this study are available from the corresponding author upon reasonable request.

## Declaration of interests

RHJM reports research funding (all paid to the institute) from Astellas, Bayer, Boehringer-Ingelheim, Cristal Therapeutics, Deuter Oncology, Echo Pharmaceuticals, Nordic Pharma, Novartis, Pamgene, Pfizer, Roche, Sanofi, and Servier, all not related to the current topic. All remaining authors have declared no conflicts of interest.
