# Supplementary material for: Survival of patients with colorectal or pancreatic cancer who received UGT1A1 genotype-guided dosing of irinotecan in the Netherlands (2017–2024): a retrospective, multicentre cohort study
Source: Lancet Reg Health Eur. 2026 Feb 23;64:101629. doi: 10.1016/j.lanepe.2026.101629 (PMC12950460; doi:10.1016/j.lanepe.2026.101629)
Supplement: Supplementary Material [file mmc1.pdf]

Supplementary material

Survival of patients with colorectal or pancreatic cancer who received *UGT1A1* genotype-guided dosing of irinotecan in The Netherlands (2017-2024): a retrospective, multicentre cohort study

Sofía L.J. Peeters, PharmD*, Niels Heersche, MD*, Doortje Böhm, PharmD, Stefan Böhringer, MD, PhD, Roselien Guiljam, BSc, Emma C. Hulshof, PharmD, PhD, Femke M. de Man, MD, PhD, Mirjam de With, MD, PhD, Marije Joosse, BSc, Gysella Oomens, BSc, Qiong-Yi Wu, BSc, Aisha Osman, BSc, Sander Bins, MD, PhD, Irene E.G. van Hellemond, MD, PhD, Brigitte C.M. Haberkorn, MD, PhD, Arjan J. Verschoor, MD, PhD, Miriam L. Wumkes, MD, PhD, Ron H.N. van Schaik, PhD, Anna M. Thijs, MD, PhD, Hans Gelderblom, MD, PhD, Henk-Jan Guchelaar, PharmD, PhD, Ron H.J. Mathijssen, MD, PhD†, Maarten J. Deenen, PharmD, PhD†

* These authors share first authorship † These authors share senior authorship

Supplementary Results

Supplementary Table S1. Baseline characteristics of patients included in the primary analysis vs patients excluded from the primary analysis.

Supplementary Table S2. Genotype frequencies and Hardy-Weinberg equilibria.

Supplementary Figure S1. *UGT1A1* gene overview and linkage disequilibria.

Supplementary Table S3. Baseline characteristics per CRC and PC patient groups – primary analysis.

Supplementary Table S4. Univariable Cox regression analyses – primary analysis.

Supplementary Table S5. Baseline characteristics – secondary analysis in all genotyped patients treated with systemic irinotecan.

Supplementary Figure S2. Kaplan-Meier plots for PFS and OS of UGT1A1 PM and IM/NM patients with colorectal and pancreatic cancer – secondary analysis in all genotyped patients treated with systemic irinotecan.

Supplementary Table S6. PFS and OS with corresponding HRs in UGT1A1 PM *vs* IM/NM patients – secondary analysis in all genotyped patients treated with systemic irinotecan.

Supplementary Table S7. Univariable Cox regression analyses – secondary analysis in all genotyped patients treated with systemic irinotecan.

Supplementary Table S8. Toxicity outcomes and irinotecan dose modifications – secondary analysis in all genotyped patients treated with systemic irinotecan.

Supplementary Figure S3. Kaplan-Meier plots for PFS of dose-reduced UGT1A1 PM patients and fully-dosed IM/NM patients – secondary analysis stratified by tumour type and treatment setting.

Supplementary Figure S4. Kaplan-Meier plots for OS of dose-reduced UGT1A1 PM patients and fully-dosed IM/NM patients – secondary analysis stratified by tumour type and treatment setting.

Supplementary Table S9. PFS and OS in UGT1A1 dose-reduced PM patients vs fully-dosed IM/NM patients – secondary analysis stratified by tumour type and treatment setting.

Supplementary Table S10. HRs of dose-reduced UGT1A1 PM patients vs fully-dosed IM/NM patients secondary analysis stratified by tumour type and treatment setting.

Supplementary Table S11. Univariable Cox regression analyses – secondary analysis stratified by tumour type and treatment setting.

Supplementary Table S12. HRs of dose-reduced UGT1A1 PM patients vs fully-dosed IM/NM patients – subgroup analyses per tumour type and treatment setting.

Supplementary Table S5. Kaplan-Meier plots for PFS and OS of dose-reduced UGT1A1 PM patients and fully-dosed IM/NM patients – subgroup analysis in patients with pancreatic cancer with standard or modified FOLFIRINOX.

Supplementary Table S13. PFS and OS in dose-reduced UGT1A1 PM patients vs fully-dosed IM/NM patients – subgroup analysis in patients with pancreatic cancer with standard or modified FOLFIRINOX**.**

Supplementary Table S14. HRs of dose-reduced UGT1A1 PM patients vs fully-dosed IM/NM patients – subgroup analysis in patients with pancreatic cancer with standard or modified FOLFIRINOX.

Supplementary Table S15. Toxicity outcomes – subgroup analysis in patients with pancreatic cancer with standard or modified FOLFIRINOX.

Supplementary Methods

Supplementary methods.

Study protocol.

Statistical analysis plan.

Table S1. Baseline characteristics of patients included in the primary analysis vs patients excluded from the primary analysis.

	**Excluded from primary analysis[a] (Not treated conform protocol) *N* = 210**	**Included in primary analysis[b] (Treated conform protocol) *N* = 779**
**UGT1A1 group, *N* (%)**		
IM/NM	186 (88·6)	703 (90·2)
PM	24 (11·4)	76 (9·8)
**Age group, *N* (%)**		
< 65 years	95 (45·2)	482 (61·9)
≥ 65 years	115 (54·8)	297 (38·1)
**Sex, *N*· (%)**		
Male	118 (56·2)	432 (55·5)
Female	92 (43·8)	347 (44·5)
**Ethnic origin, *N* (%)**		
European	186 (88·6)	715 (91·8)
Other	24 (11·4)	64 (8·2)
**WHO performance, *N* (%)**		
0-1	187 (89·0)	744 (95·5)
2-3	23 (11·0)	35 (4·5)
**Smoking status, *N* (%)**		
Never or Former smoker	184 (87·6)	632 (81·1)
Smoker	20 (9·5)	118 (15·1)
Unknown	6 (2·9)	29 (3·7)
**Tumour type, *N* (%)**		
Colorectal cancer	112 (53·3)	383 (49·2)
Pancreatic cancer	98 (46·7)	396 (50·8)
**Tumour stage, *N* (%)**		
Stage I	10 (4·8)	41 (5·3)
Stage II	20 (9·5)	96 (12·3)
Stage III	39 (18·6)	190 (24·4)
Stage IV	141 (67·1)	452 (58·0)
**Treatment regimen + irinotecan dosage, *N* (%)**		
FOLFIRINOX (180 mg/m^2 q2w)	71 (33·8)	275 (35·3)
mFOLFIRINOX (150 mg/m^2 q2w)	26 (12·4)	121 (15·5)
Irinotecan monotherapy – low dose	13 (6·2)	39 (5·0)
Irinotecan monotherapy – high dose	34 (16·2)	45 (5·8)
FOLFIRI (180 mg/m^2 q2w)	23 (11·0)	107 (13·7)
FOLFIRI + bevacizumab/cetuximab (180 mg/m^2 q2w)	10 (4·8)	57 (7·3)
FOLFOXIRI (165 mg/m^2 q2w)	5 (2·4)	44 (5·6)
FOLFOXIRI + bevacizumab (165 mg/m^2 q2w)	22 (10·5)	60 (7·7)
Other	6 (2·9)	31 (4·0)
**Previous surgery, *N* (%)**		
Yes	121 (57·6)	461 (59·2)
No	89 (42·4)	318 (40·8)
**Previous radiotherapy, *N* (%)**		
Yes	175 (83·3)	633 (81·3)
No	35 (16·7)	146 (18·7)
**Previous chemotherapy, *N* (%)**		
Yes	115 (54·8)	451 (57·9)
No	95 (45·2)	328 (42·1)
**Previous number of lines, *N* (%)**		
0	138 (65·7)	576 (73·9)
1	58 (27·6)	175 (22·5)
2	10 (4·8)	23 (3·0)
3	4 (1·9)	5 (0·6)
**Previous treatment with irinotecan, *N* (%)**		
Yes	204 (97·1)	764 (98·1)
No	6 (2·9)	15 (1·9)
**Irinotecan RDI cycle 1, median (IQR)**	75 (73-80)	100 (97-101)

BSA, Body Surface Area; *IM,* intermediate metaboliser; *IQR*, interquartile range; *NM,* normal metaboliser; *N,* number of patients; *PM,* poor metaboliser; *RDI,* relative dose intensity; *UGT1A1,* Uridine Diphosphate Glucuronosyltransferase 1A1; *WHO,* World Health Organization.

[a] Patients excluded from the primary analysis did not receive an irinotecan dose according to *UGT1A1* genotype in cycle 1. These patients were only included in the secondary analysis of all *UGT1A1* genotyped patients regardless of irinotecan dose in cycle 1.

[b] Patients included in the primary analysis received an irinotecan dose according to *UGT1A1* genotype in at least cycle 1.

Table S2. Genotype frequencies and Hardy-Weinberg equilibria.

***UGT1A1* variant**[a]	**rs number**	***N***	**Homozygous wild-type *N* (%)**	**Heterozygous *N* (%)**	**Homozygous variant *N* (%)**	**MAF (%)**	**HWE *P* value**
***UGT1A1**28 (TA7)**	rs3064744	989	471 (48%)	418 (42%)	100 (10%)	31	0·61
***UGT1A1**36 (TA5)**	rs3064744	220	220 (100%)	0 (0%)	0 (0%)	0	NA
***UGT1A1**37 (TA8)**	rs3064744	471	469 (100%)	2 (0%)	0 (0%)	0	0·96
***UGT1A1**93 (-3156G>A)**	rs10929302	651	344 (53%)	260 (40%)	47 (7%)	27	0·82
***UGT1A1**6 (c.211G>A)**	rs4148323	52	51 (98%)	1 (2%)	0 (0%)	1	0·94
***UGT1A1**80 (-364C>T)**	rs887829	31	16 (52%)	11 (35%)	4 (13%)	31	0·36

HWE, Hardy-Weinberg equilibrium; *MAF,* minor allele frequency; *N*, number of patients; *NA*, not applicable; *rs number*, international reference number, *UGT1A1,* Uridine Diphosphate Glucuronosyltransferase 1A1 gene.

[a] *UGT1A1*28, UGT1A1*37, UGT1A1*93, UGT1A1*6,* and *UGT1A1*80* are associated with a reduced functionality and metabolic capacity of the UGT1A1 enzyme. *UGT1A1**36 is associated with an increased functionality and metabolic capacity of the UGT1A1 enzyme. In our cohort, *UGT1A1*80* and *UGT1A1*93* were only identified in patients who also had *UGT1A1*28* or *UGT1A1*37.*

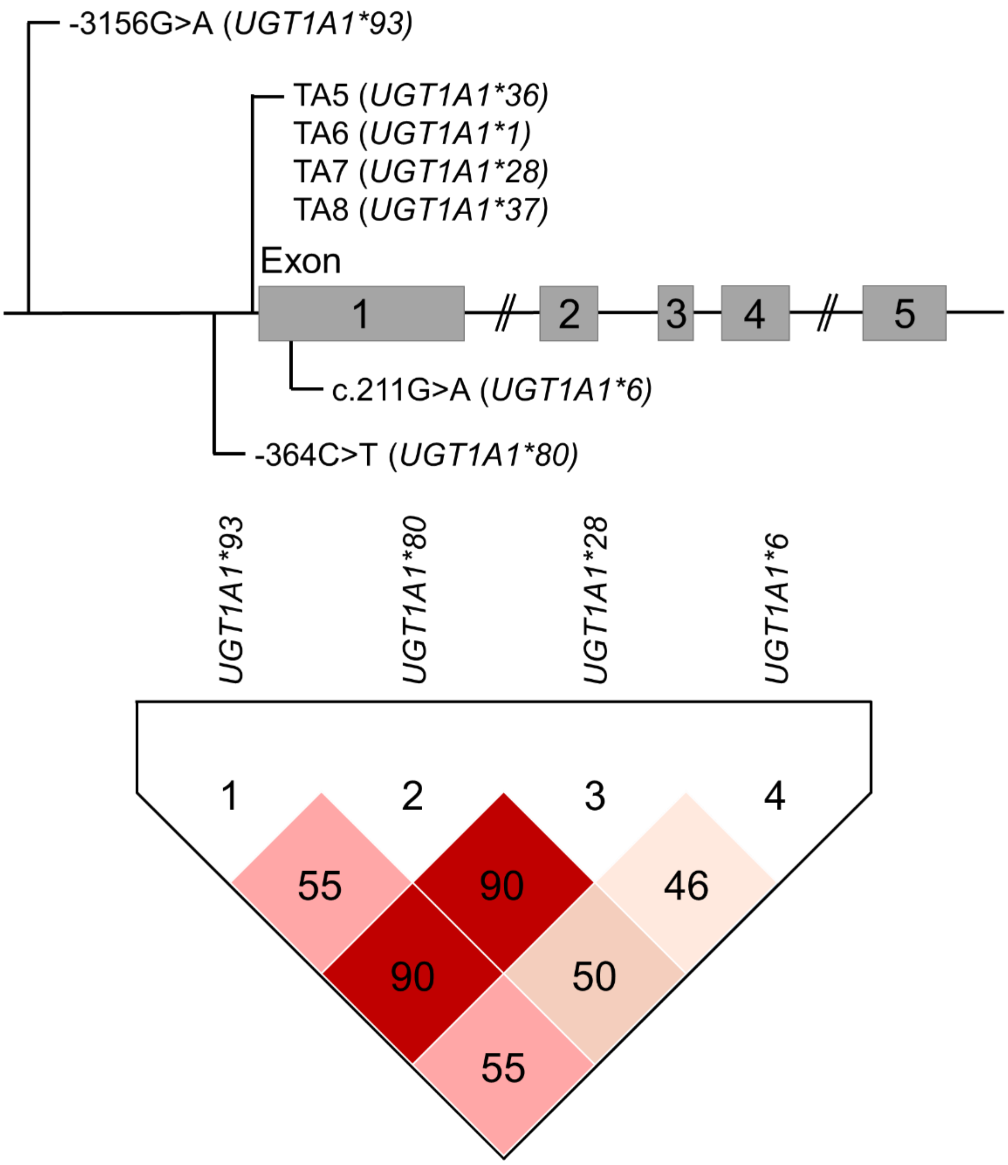

Figure S1. UGT1A1 Gene Overview and Linkage Disequilibria.
UGT1A1, Uridine Diphosphate Glucuronosyltransferase 1A1 gene.

Table S3. Baseline characteristics per CRC and PC patient groups – primary analysis.

	**COLORECTAL CANCER**			**PANCREATIC CANCER**		
	**UGT1A1 PM** *N* = 35	**UGT1A1 IM/NM** *N* = 348	**ALL PATIENTS** *N* = 383	**UGT1A1 PM** *N* = 41	**UGT1A1 IM/NM** *N* = 355	**ALL PATIENTS** *N* = 396
**Age in years, median (IQR)**	62 (54-69)	60 (53-68)	60 (53-68)	62 (57-69)	63 (56-69)	63 (56-69)
**Sex, *N* (%)**						
Male	24 (68·6)	202 (58·0)	226 (59·0)	17 (41·5)	189 (53·2)	206 (52·0)
Female	11 (31·4)	146 (42·0)	157 (41·0)	24 (58·5)	166 (46·8)	190 (48·0)
**Ethnic origin, *N* (%)**						
European	30 (85·7)	314 (90·2)	344 (89·8)	26 (87·8)	335 (94·4)	371 (93·7)
Middle-Eastern	2 (5·7)	9 (2·6)	11 (2·9)	1 (2·4)	7 (2·0)	8 (2·0)
North-African	0 (0)	6 (1·7)	6 (1·6)	0 (0)	4 (1·1)	4 (1·0)
Sub-Saharan African	0 (0)	4 (1·1)	4 (1·0)	0 (0)	1 (0·3)	1 (0·3)
Asian	0 (0)	3 (0·9)	3 (0·8)	1 (2·4)	2 (0·6)	3 (0·8)
Hispanic	2 (5·7)	9 (2·6)	11 (2·9)	2 (4·9)	4 (1·1)	6 (1·5)
Other	0 (0)	1 (0·3)	1 (0·3)	1 (2·4)	0 (0)	1 (0·3)
Unknown	1 (2·9)	2 (0·6)	3 (0·8)	0 (0)	2 (0·6)	2 (0·5)
**BSA in m^2, median (IQR)**	1·97 (1·81-2·04)	1·94 (1·79-2·08)	1·95 (1·80-2·08)	1·78 (1·70-2·01)	1·90 (1·76-2·03)	1·89 (1·75-2·03)
**WHO performance, *N* (%)**						
0-1	34 (97·1)	334 (96·0)	368 (96·1)	38 (92·7)	338 (95·2)	376 (94·9)
2-3	1 (2·9)	14 (4·0)	15 (3·9)	3 (7·3)	17 (4·8)	20 (5·1)
**Smoking status, *N* (%)**						
Never	18 (51·4)	161 (46·3)	179 (46·7)	12 (29·3)	114 (32·1)	126 (31·8)
Smoker	1 (2·9)	39 (11·2)	40 (10·4)	11 (26·8)	67 (18·9)	78 (19·7)
Ex-smoker	16 (45·7)	132 (37·9)	148 (38·6)	16 (39·0)	163 (45·9)	179 (45·2)
Unknown	0 (0)	16 (4·6)	16 (4·2)	2 (4·9)	11 (3·1)	13 (3·3)
**Tumour stage, *N* (%)**						
Stage I	0 (0)	0 (0)	0 (0)	1 (2·4)	40 (11·3)	41 (10·4)
Stage II	0 (0)	9 (2·6)	0 (2·3)	12 (29·3)	12 (21·1)	89 (22·0)
Stage III	2 (5·7)	44 (12·6)	46 (12·0)	15 (36·6)	129 (36·3)	144 (36·4)
Stage IV	33 (94·3)	295 (84·8)	328 (85·6)	13 (31·7)	111 (31·1)	124 (31·3)
**Treatment regimen + standard irinotecan dose, *N* (%)**						
FOLFIRINOX (180 mg/m^2 q2w)	0 (0)	0 (0)	0 (0)	32 (78·0)	243 (68·5)	275 (69·4)
mFOLFIRINOX (150 mg/m^2 q2w)	0 (0)	0 (0)	0 (0)	9 (22·0)	112 (31·5)	121 (30·6)
Irinotecan monotherapy – low dose[a]	3 (8·6)	36 (10·3)	39 (10·2)	0 (0)	0 (0)	0 (0)
Irinotecan monotherapy – high dose[b]	6 (17·1)	39 (11·2)	45 (11·7)	0 (0)	0 (0)	0 (0)
FOLFIRI (180 mg/m^2 q2w)	10 (28·6)	97 (27·9)	107 (27·9)	0 (0)	0 (0)	0 (0)
FOLFIRI + bevacizumab/cetuximab (180 mg/m^2 q2w)	4 (11·4)	53 (15·2)	57 (14·9)	0 (0)	0 (0)	0 (0)
FOLFOXIRI (165 mg/m^2 q2w)	1 (2·9)	43 (12·4)	44 (11·5)	0 (0)	0 (0)	0 (0)
FOLFOXIRI + bevacizumab (165 mg/m^2 q2w)	7 (20·0)	53 (15·2)	60 (15·7)	0 (0)	0 (0)	0 (0)
Other[c]	4 (11·4)	27 (7·8)	31 (8·1)	0 (0)	0 (0)	0 (0)
**Previous surgery, *N* (%)**						
Yes	22 (62·9)	208 (59·8)	230 (60·1)	7 (17·1)	81 (22·8)	88 (22·2)
No	13 (37·1)	140 (40·2)	153 (39·9)	34 (82·9)	274 (77·2)	308 (77·8)
**Previous radiotherapy, *N* (%)**						
Yes	15 (42·9)	106 (30·5)	121 (31·6)	3 (7·3)	22 (6·2)	25 (6·3)
No	20 (57·1)	242 (69·5)	262 (68·4)	38 (92·7)	333 (93·8)	371 (93·7)
**Previous chemotherapy, *N* (%)**						
Yes	7 (20·0)	85 (24·4)	92 (24·0)	3 (7·3)	34 (9·6)	37 (9·3)
No	28 (80·0)	263 (75·6)	291 (76·0)	38 (92·7)	321 (90·4)	359 (90·7)
**Previous number of lines, *N* (%)**						
0	14 (40·0)	167 (48·0)	181 (47·3)	41 (100)	354 (99·7)	395 (99·7)
1	19 (54·3)	155 (44·5)	174 (45·4)	0 (0)	1 (0·3)	1 (0·3)
2	2 (5·7)	21 (6·0)	23 (6·0)	0 (0)	0 (0)	0 (0)
3	0 (0)	5 (1·4)	5 (1·3)	0 (0)	0 (0)	0 (0)
**Previous treatment with irinotecan, *N* (%)**						
Yes	3 (8·6)	9 (2·6)	12 (3·1)	0 (0)	3 (0·8)	3 (0·8)
No	32 (91·4)	339 (97·4)	371 (96·9)	41 (100)	352 (99·2)	393 (99·2)

BSA, Body Surface Area; *IM,* intermediate metaboliser; *IQR*, interquartile range; *NM,* normal metaboliser; *N,* number of patients; *PM,* poor metaboliser; *UGT1A1,* Uridine Diphosphate Glucuronosyltransferase 1A1; *WHO,* World Health Organization.

[a] Irinotecan monotherapy low dose: 180 mg/m2 or 450 mg flat dose either q2w or q3w

[b] Irinotecan monotherapy high dose: 350 mg/m2 or 600 mg flat dose either q2w or q3w

[c] Other treatment regimens included: irinotecan monotherapy 210 mg/m^2 (*N* = 13), irinotecan + panitumumab 180 mg/m^2 q2w (*N* = 9), FOLFIRI + panitumumab 180mg/m^2 q2w (*N* = 8), FOLFIRI 180 mg/m^2 q3w (*N* = 1), FOLFIRI + ramucirumab 180 mg/m^2 q2w (*N* = 2), mFOLFOXIRI + panitumumab 150 mg/m^2 q2w (*N* = 1), irinotecan + cetuximab 150 mg/m^2 q2w (*N* = 1), irinotecan + bevacizumab 180 mg/m^2 (*N* = 1), irinotecan monotherapy flat 240mg q2w (*N* = 1).

Table S4. Univariable Cox regression analyses – primary analysis.

Variables	Univariable analysis[a] PFS		Univariable analysis[a] OS	
	HR (*95%CI*)	*P* value	HR (*95%CI*)	*P* value
**UGT1A1 group**				
IM/NM (reference group)	-	-	-	-
PM	1·08 *(0·84-1·40)*	0·54	1·13 *(0·84-1·51)*	0·42
**Sex**				
Male (reference group)	-	-	-	-
Female	0·95 *(0·81-1·11)*	0·48	0·99 *(0·83-1·17)*	0·87
**Age**				
< 65 years (reference group)	-	-	-	-
≥ 65 years	1·23 *(1·05-1·44)*	0·01	1·19 *(1·00-1·41)*	0·05
**WHO performance status**				
0 – 1 (reference group)	-	-	-	-
2 – 3	1·62 *(1·13-2·31)*	0·01	1·85 *(1·26-2·72)*	0·002
**Ethnicity**				
European (reference group)	-	-	-	-
Other	0·77 *(0·57-1·04)*	0·09	0·96 *(0·69-1·33)*	0·81
**Smoking status**				
Former or Never (reference group)	-	-	-	-
Current	1·04 *(0·83-1·29)*	0·76	1·18 *(0·93-1·50)*	0·17
Unknown	1·12 *(0·74-1·70)*	0·60	1·24 *(0·77-1·99)*	0·37
**Tumour Type**				
Colorectal (reference group)	-	-	-	-
Pancreatic	0·76 *(0·65-0·89)*	<0·001	1·09 *(0·92-1·29)*	0·35
**Tumour Stage**				
I (reference group)	-	-	-	-
II	1·82 *(1·10-3·04)*	0·02	1·90 *(1·04-3·49)*	0·04
III	2·52 *(1·55-4·08)*	<0·001	2·55 *(1·42-4·49)*	0·002
IV	8·67 *(5·34-14·09)*	<0·001	9·13 *(5·14-16·23)*	<0·001
**Treatment Regimen**				
FOLFIRINOX (180 mg/m^2) (reference group)	-	-	-	-
mFOLFIRINOX (150 mg/m^2)	0·43 *(0·33-0·55)*	<0·001	0·42 *(0·32-0·56)*	<0·001
Irinotecan monotherapy – low[b]	1·19 *(0·16-8·72)*	0·86	2·79 *(0·38-20·66)*	0·31
Irinotecan monotherapy – high[c]	1·49 *(0·21-10·82)*	0·69	2·94 *(0·40-21·55)*	0·29
FOLFIRI (180 mg/m^2)	1·17 *(0·16-8·43)*	0·87	2·22 *(0·31-16·03)*	0·43
FOLFIRI + B/C (180 mg/m^2)	0·52 *(0·07-3·78)*	0·52	1·54 *(0·21-11·24)*	0·67
FOLFOXIRI (165 mg/m^2)	0·19 *(0·03-1·46)*	0·11	0·52 *(0·07-4·10)*	0·53
FOLFOXIRI + B (165 mg/m^2)	0·34 *(0·05-2·45)*	0·28	0·65 *(0·09-4·87)*	0·67
Other[d]	2·10 *(0·29-15·38)*	0·47	4·12 *(0·56-30·50)*	0·17
**Previous surgery**				
No (reference group)	-	-	-	-
Yes	1·07 *(0·91-1·26)*	0·43	0·84 *(0·70-1·02)*	0·07
**Previous radiotherapy**				
No (reference group)	-	-	-	-
Yes	1·36 *(1·11-1·66)*	0·003	1·22 *(0·97-1·53)*	0·09
**Previous chemotherapy**				
No (reference group)	-	-	-	-
Yes	2·13 *(1·76-2·58)*	<0·001	1·85 *(1·46-2·33)*	<0·001
**Previous irinotecan treatment**				
No (reference group)	-	-	-	-
Yes	1·50 *(0·86-2·60)*	0·15	1·06 *(0·55-2·05)*	0·87
**Previous number of lines**				
0 (reference group)	-	-	-	-
1	3·78 *(2·97-4·80)*	<0·001	2·75 *(2·10-3·59)*	<0·001
2	3·67 *(2·32-5·80)*	<0·001	3·00 *(1·78-5·08)*	<0·001
3	5·78 *(2·34-14·29)*	<0·001	6·06 *(2·44-15·04)*	<0·001

CI, confidence interval; *HR,* hazard ratio; *IM,* intermediate metaboliser; *NM,* normal metaboliser; *PM,* poor metaboliser; *UGT1A1,* Uridine Diphosphate Glucuronosyltransferase 1A1; *WHO,* World Health Organization.

[a]A stratified Cox model was used for all Cox regression analyses (stratified by tumour type), except for univariable analysis of tumour type.

[b] Irinotecan monotherapy low dose: 180 mg/m^2 or 450 mg flat dose either q2w or q3w

[c] Irinotecan monotherapy high dose: 350 mg/m^2 or 600 mg flat dose either q2w or q3w

[d] Other treatment regimens and respective standard irinotecan dosages included: irinotecan monotherapy 210 mg/m^2 (*N* = 10), irinotecan + panitumumab 180 mg/m^2 q2w (*N* = 8), FOLFIRI + panitumumab 180mg/m^2 q2w (*N* = 7), FOLFIRI 180 mg/m^2 q3w (*N* = 1), FOLFIRI + ramucirumab 180 mg/m^2 q2w (*N* = 1), mFOLFOXIRI + panitumumab 150 mg/m^2 q2w (*N* = 1), irinotecan + cetuximab 150 mg/m^2 q2w (*N* = 1), irinotecan + bevacizumab 180 mg/m^2 (*N* = 1), irinotecan monotherapy 240mg flat q2w (*N* = 1).

Table S5. Baseline characteristics – secondary analysis in all genotyped patients treated with systemic irinotecan.

	**UGT1A1 PM** *N* **= 100**	**UGT1A1 IM/NM** *N* **= 889**	**ALL PATIENTS[d]** *N* **= 989**
**Age in years, median (IQR)**	62 (55-69)	63 (55-69)	63 (55-69)
**Sex,** ***N*** **(%)**			
Male	56 (56·0)	494 (55·6)	550 (55·6)
Female	44 (44·0)	395 (44·4)	439 (44·4)
**Ethnic origin,** ***N*** **(%)**			
European	88 (88·0)	813 (91·5)	901 (91·1)
Middle-Eastern	4 (4·0)	22 (2·5)	11 (1·1)
North-African	0 (0·0)	11 (1·2)	26 (2·6)
Sub-Saharan African	1 (1·0)	6 (0·7)	7 (0·7)
Asian	1 (1·0)	8 (0·9)	9 (0·9)
Hispanic	4 (4·0)	21 (2·4)	25 (2·5)
Other	1 (1·0)	1 (0·1)	2 (0·2)
Unknown	1 (1·0)	7 (0·8)	8 (0·8)
**BSA in m^2, median (IQR)**	1·91 (1·75-2·04)	1·92 (1·77-2·06)	1·92 (1·77-2·06)
**WHO performance,** ***N*** **(%)**			
0-1	95 (95·0)	836 (94·0)	931 (94·1)
2-3	5 (5·0)	53 (6·0)	58 (5·9)
**Smoking status,** ***N*** **(%)**			
Never	41 (41·0)	358 (40·3)	399 (40·3)
Smoker	13 (13·0)	125 (14·1)	138 (14·0)
Ex-smoker	44 (44·0)	373 (42·0)	417 (42·2)
Unknown	2 (2·0)	33 (3·7)	35 (3·5)
**Tumour type,** ***N*** **(%)**			
Colorectal cancer	51 (51·0)	444 (49·9)	495 (50·1)
Pancreatic cancer	49 (49·0)	445 (50·1)	494 (49·9)
**Tumour stage,** ***N*** **(%)**			
Stage I	1 (1·0)	50 (5·6)	51 (5·2)
Stage II	14 (14·0)	102 (11·5)	116 (11·7)
Stage III	22 (22·0)	207 (23·3)	229 (23·2)
Stage IV	63 (63·0)	530 (59·6)	593 (60·0)
**Treatment regimen + standard irinotecan dosage,** ***N*** **(%)**			
FOLFIRINOX (180 mg/m^2 q2w)	38 (38·0)	309 (34·8)	347 (35·1)
mFOLFIRINOX (150 mg/m^2 q2w)	11 (11·0)	136 (15·3)	147 (14·9)
Irinotecan monotherapy – low dose[a]	4 (4·0)	48 (5·4)	52 (5·3)
Irinotecan monotherapy – high dose[b]	11 (11·0)	68 (7·6)	79 (8·0)
FOLFIRI (180 mg/m^2 q2w)	14 (14·0)	116 (13·0)	130 (13·1)
FOLFIRI + bevacizumab/cetuximab (180 mg/m^2 q2w)	4 (4·0)	62 (7·0)	66 (6·7)
FOLFOXIRI (165 mg/m^2 q2w)	3 (3·0)	46 (5·2)	49 (5·0)
FOLFOXIRI + bevacizumab (165 mg/m^2 q2w)	9 (9·0)	73 (8·2)	82 (8·3)
Other[c]	6 (6·0)	31 (3·5)	37 (3·7)
**Previous surgery,** ***N*** **(%)**			
Yes	42 (42·0)	365 (41·1)	407 (41·2)
No	58 (58·0)	524 (58·9)	582 (58·8)
**Previous radiotherapy,** ***N*** **(%)**			
Yes	21 (21·0)	160 (18·0)	181 (18·3)
No	79 (79·0)	729 (82·0)	808 (81·7)
**Previous chemotherapy,** ***N*** **(%)**			
Yes	46 (46·0)	377 (42·4)	423 (42·8)
No	54 (54·0)	512 (57·6)	566 (57·2)
**Previous number of lines,** ***N*** **(%)**			
0	69 (69·0)	645 (72·6)	714 (72·2)
1	28 (28·0)	205 (23·1)	233 (23·6)
2	2 (2·0)	31 (3·5)	33 (3·3)
3	1 (1·0)	8 (0·9)	9 (0·9)
**Previous treatment with irinotecan,** ***N*** **(%)**			
Yes	4 (4·0)	17 (1·9)	21 (2·1)
No	96 (96·0)	872 (98·1)	968 (97·9)

BSA, Body Surface Area; *IM,* intermediate metaboliser; *IQR*, interquartile range; *NM,* normal metaboliser; *N,* number of patients; *PM,* poor metaboliser; *UGT1A1,* Uridine Diphosphate Glucuronosyltransferase 1A1; *WHO,* World Health Organization.

[a] Irinotecan monotherapy low dose: 180 mg/m2 or 450 mg flat dose either q2w or q3w

[b] Irinotecan monotherapy high dose: 350 mg/m2 or 600 mg flat dose either q2w or q3w

[c] Other treatment regimens included: irinotecan monotherapy 210 mg/m^2 (*N* = 13), irinotecan + panitumumab 180 mg/m^2 q2w (*N* = 9), FOLFIRI + panitumumab 180mg/m^2 q2w (*N* = 8), FOLFIRI 180 mg/m^2 q3w (*N* = 1), FOLFIRI + ramucirumab 180 mg/m^2 q2w (*N* = 2), mFOLFOXIRI + panitumumab 150 mg/m^2 q2w (*N* = 1), irinotecan + cetuximab 150 mg/m^2 q2w (*N* = 1), irinotecan + bevacizumab 180 mg/m^2 (*N* = 1), irinotecan monotherapy flat 240mg q2w (*N* = 1).

[d] Of the total 989 patients, 324 patients were included from the study by Hulshof *et al.* and 665 patients were newly identified from routine clinical care.

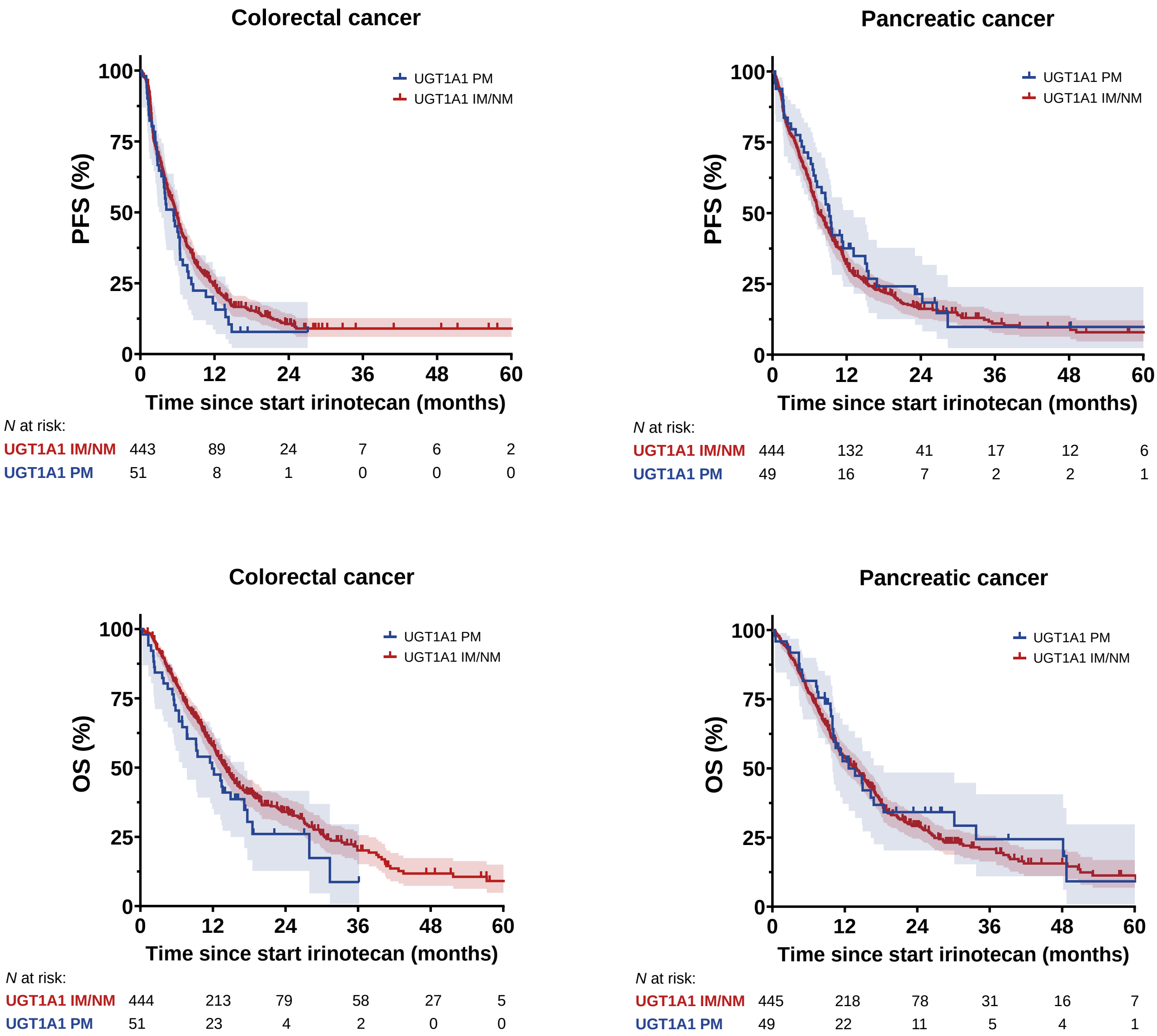

Figure S2. Kaplan-Meier plots for PFS and OS of UGT1A1 PM and IM/NM patients with colorectal and pancreatic cancer – secondary analysis in all genotyped patients treated with systemic irinotecan.

Censoring is indicated by tick marks. The shaded area represents the 95% CI. A stratified log-rank test (stratified by tumour type) for comparing Kaplan-Meier curves between PM and IM/NM showed a *P* value of *P* = 0·72 for PFS and *P* = 0·52 for OS. *IM*, intermediate metaboliser; *NM*, normal metaboliser; *N*, number of patients; *OS*, overall survival; *PFS*, progression-free survival; *PM*, poor metaboliser; *UGT1A1*, Uridine Diphosphate Glucuronosyltransferase 1A1.

Table S6. PFS and OS with corresponding HRs in UGT1A1 PM vs IM/NM patients – secondary analysis in all genotyped patients treated with systemic irinotecan.

	***N*[a]**	**PFS events, *N* (%)**	**Median PFS, months**	**95% CI, months**	**1-Year PFS, %**	**95% CI, %**	***N***	**Deceased, *N* (%)**	**Median OS, months**	**95% CI, months**	**1-Year OS, %**	**95% CI, %**
Colorectal												
IM/NM	443	373 (84·2)	5·7	5·0-6·5	23·9	19·8-28·0	444	291 (65·5)	14·1	12·7-15·5	57.8	53.1-62.5
PM	51	45 (88·2)	5·4	3·3-7·5	15·7	5·3-26·1	51	35 (68·6)	11·9	7·3-16·4	47.5	33.4-61.6
Pancreatic												
IM/NM	444	368 (82·9)	7·6	6·7-8·5	32·8	28·3-37·3	445	318 (71·5)	13·9	11·9-15·9	53.9	49.2-58.6
PM	49	39 (79·6)	9·2	7·9-10·5	37·5	23·6-51·4	49	33 (67·3)	12·6	7·6-17·7	52.6	38.1-67.1

	***N*[a]**	**PFS events, *N* (%)**	**Univariable analysis PFS[b]**		**Multivariable analysis PFS[c]**		***N***	**Deceased, *N* (%)**	**Univariable analysis OS[b]**		**Multivariable analysis OS[d]**	
			**HR (*95%CI*)**	***P* value**	**HR (*95%CI*)**	***P* value**			**HR (*95%CI*)**	***P* value**	**HR (*95%CI*)**	***P* value**
All patients												
IM/NM	887	585 (83·3)	-	-	-	-	889	476 (67·8)	-	-	-	-
PM	100	64 (84·2)	1·04 *(0·83-1·31)*	0·72	0·95 *(0·71-1·27)*	0·74	100	51 (67·1)	1·09 *(0·84-1·40)*	0·52	0·84 *(0·61-1·17)*	0·30

CI, confidence interval; *HR*, hazard ratio; *IM*, intermediate metaboliser; *NM*, normal metaboliser; *N*, number of patients; *OS*, overall survival; *PFS*, progression-free survival; *PM*, poor metaboliser; *UGT1A1*, Uridine Diphosphate Glucuronosyltransferase 1A1.

[a] Two IM/NM patients were excluded from PFS analysis because of an unknown disease progression date.

[b] Univariable Cox regression analysis stratified by tumour type.

[c] Multivariable Cox regression PFS analysis stratified by tumour type and adjusted for covariates age, WHO performance status, tumour stage, treatment regimen, previous radiotherapy, previous chemotherapy, and previous number of chemotherapy lines.

[d] Multivariable Cox regression OS analysis stratified by tumour type and adjusted for covariates age, WHO performance status, tumour stage, treatment regimen, previous chemotherapy and previous number of chemotherapy lines.

Table S7. Univariable Cox regression analyses – secondary analysis in all genotyped patients treated with systemic irinotecan.

Variables	Univariable analysis[a] PFS		Univariable analysis[a] OS	
	HR (*95%CI*)	*P* value	HR (*95%CI*)	*P* value
**UGT1A1 group**				
IM/NM (reference group)	-	-	-	-
PM	1·04 *(0·83-1·31)*	0·72	1·09 *(0·84-1·40)*	0·52
**Sex**				
Male (reference group)	-	-	-	-
Female	0·98 *(0·85-1·12)*	0·75	1·04 *(0·90-1·22)*	0·59
**Age**				
< 65 years (reference group)	-	-	-	-
≥ 65 years	1·20*(1·04-1·38)*	0·01	1·15 *(0·99-1·34)*	0·07
**WHO performance status**				
0 – 1 (reference group)	-	-	-	-
2 – 3	1·77 *(1·34-2·34)*	<0·001	2·10 *(1·56-2·83)*	<0·001
**Ethnicity**				
European (reference group)	-	-	-	-
Other	0·84 *(0·65-1·08)*	0·18	0·95 *(0·73-1·26)*	0·74
**Smoking status**				
Former or Never (reference group)	-	-	-	-
Current	1·04 *(0·85-1·27)*	0·73	1·13 *(0·91-1·41)*	0·28
Unknown	1·16 *(0·80-1·69)*	0·43	1·31 *(0·87-1·97)*	0·20
**Tumour Type**				
Colorectal (reference group)	-	-	-	-
Pancreatic	0·75 *(0·65-0·86)*	<0·001	1·03 *(0·89-1·20)*	0·69
**Tumour Stage**				
I (reference group)	-	-	-	-
II	1·69 *(1·08-2·66)*	0·02	1·75 *(1·04-2·96)*	0·04
III	2·37 *(1·55-3·62)*	<0·001	2·36 *(1·44-3·87)*	0·01
IV	7·43 *(4·86-11·36)*	<0·001	8·11 *(4·96-13·26)*	<0·001
**Treatment Regimen**				
FOLFIRINOX (180 mg/m^2) (reference group)	-	-	-	-
mFOLFIRINOX (150 mg/m^2)	0·45 *(0·36-0·57)*	<0·001	0·47 *(0·36-0·60)*	<0·001
Irinotecan monotherapy – low	0·61 *(0·20-1·83)*	0·38	1·18 *(0·41-3·36)*	0·76
Irinotecan monotherapy – high	0·67 *(0·23-1·97)*	0·44	1·28 *(0·46-3·61)*	0·64
FOLFIRI (180 mg/m^2)	0·53 *(0·18-1·53)*	0·24	0·93 *(0·34-2·56)*	0·89
FOLFIRI + B/C (180 mg/m^2)	0·25 *(0·08-0·75)*	0·01	0·63 *(0·22-1·79)*	0·39
FOLFOXIRI (165 mg/m^2)	0·09 *(0·03-0·28)*	<0·001	0·23 *(0·07-0·73)*	0·01
FOLFOXIRI + B (165 mg/m^2)	0·14 *(0·05-0·43)*	<0·001	0·28 *(0·09-0·81)*	0·02
Other	1·02 *(0·34-3·09)*	0·97	1·80 *(0·62-5·20)*	0·28
**Previous surgery**				
No (reference group)	-	-	-	-
Yes	1·08 *(0·93-1·25)*	0·30	0·87 *(0·74-1·03)*	0·11
**Previous radiotherapy**				
No (reference group)	-	-	-	-
Yes	1·37 *(1·14-1·63)*	<0·001	1·18 *(0·96-1·44)*	0·11
**Previous chemotherapy**				
No (reference group)	-	-	-	-
Yes	2·15 *(1·82-2·54)*	<0·001	1·82 *(1·49-2·21)*	<0·001
**Previous irinotecan treatment**				
No (reference group)	-	-	-	-
Yes	1·15 *(0·72-1·83)*	0·57	0·92 *(0·53-1·60)*	0·78
**No. of previous chemotherapy lines**				
0 (reference group)	-	-	-	-
1	3·72 *(3·01-4·60)*	<0·001	2·82 *(2·22-3·57)*	<0·001
2	3·60 *(2·43-5·33)*	<0·001	3·29 *(2·13-5·07)*	<0·001
3	4·65 *(2·35-9·20)*	<0·001	5·17 *(2·60-10·29)*	<0·001
**Relative dose intensity cycle 1**				
Per 10%	0·95 *(0·90-1·00)*	0·07	0·92 *(0·87-0·97)*	0·003

CI, confidence interval; *HR,* hazard ratio; *IM,* intermediate metaboliser; *NM,* normal metaboliser; *N,* number of patients; *PM,* poor metaboliser; *RDI,* relative dose intensity; *WHO,* World Health Organization; *UGT1A1,* Uridine Diphosphate Glucuronosyltransferase 1A1.

[a] stratified Cox model was used for all Cox regression analyses (stratified by tumour type), except for univariable analysis of tumour type.

Table S8. Toxicity outcomes and irinotecan dose modifications – secondary analysis in all genotyped patients treated with systemic irinotecan.

	UGT1A1 PM *N* = 100	UGT1A1 IM/NM *N* = 889	*P* value[f]
**Overall grade ≥ 3 toxicity[a] in the first three cycles, *N* (%)**	32 (32·0)	291 (32·7)	0·88
**Dose modification(s)[b,c] of irinotecan after the first cycle, *N* (%)**			
No dose modification	55 (55·0)	432 (48·6)	0·10
≥1 dose modification(s)	38 (38·0)	423 (46·7)	
Not applicable[d]	7 (7·0)	34 (3·8)	
**Dose reduction(s)[c] of irinotecan after the first cycle, *N* (%)[e]**	33 (35·5)	402 (47·0)	0·03
**Dose escalation(s)[c] of irinotecan after the first cycle, *N* (%)[e]**	8 (8·6)	64 (7·5)	0·70
**Relative dose intensity of irinotecan in %, median (IQR)**			
Relative dose intensity first cycle	70 (67-72)	99 (95-100)	<0·001
Relative dose intensity last cycle	67 (53-71)	81 (74-99)	<0·001
Average relative dose intensity all cycles	69 (60-71)	90 (79-99)	<0·001

IM, intermediate metaboliser; *IQR*, interquartile range; *NM,* normal metaboliser; *N,* number of patients; *PM,* poor metaboliser; *UGT1A1,* Uridine Diphosphate Glucuronosyltransferase 1A1.

[a] Overall severe toxicity included one of more of the following toxicities: Grade ≥ 3 febrile neutropenia, Grade ≥ 3 neutropenia, Grade ≥ 3 diarrhoea, irinotecan-related hospitalisation.

[b] Dose reduction and/or dose escalation.

[c] Dose reductions or escalations were defined as a change in ≥10% of irinotecan dose compared to the previous cycle.

[d] Patients that only received one treatment cycle with irinotecan.

[e] Numbers and percentages were determined only for patients that received ≥2 irinotecan treatment cycles (PM, *N* = 70; IM/NM, *N* = 679).

[f] *P* value (two-sided) comparing UGT1A1 PM with UGT1A1 IM/NM. For categorical variables Chi-Square or Fisher's Exact Test (in case cells had a ≥20% expected count less than 5) was used. For relative dose intensity Mann-Whitney U test was used.

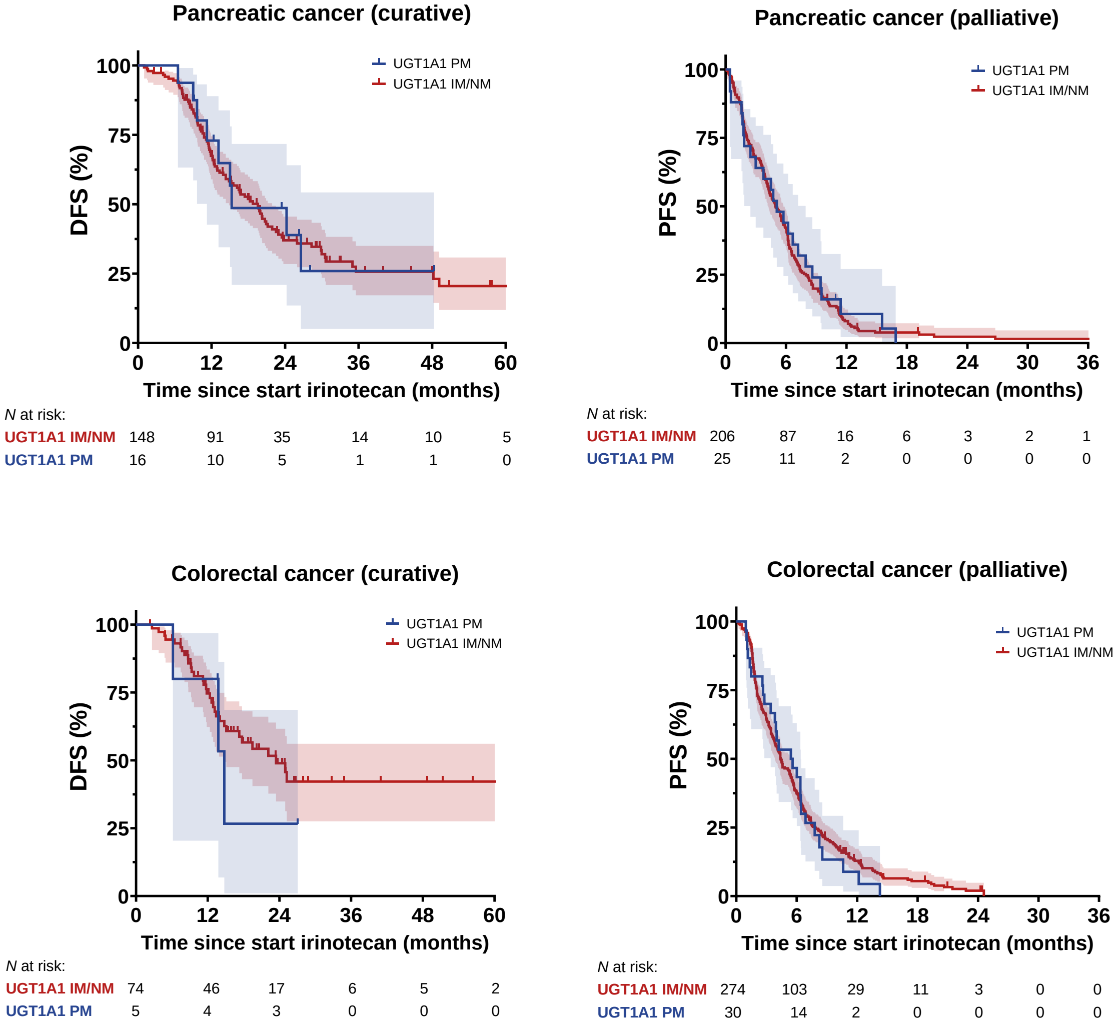

Figure S3. Kaplan-Meier plots for DFS and PFS of dose-reduced UGT1A1 PM patients and fully-dosed IM/NM patients – secondary analysis stratified by tumour type and treatment setting

Censoring is indicated by tick marks. The shaded area represents the 95% CI. A stratified log-rank test (stratified by tumour type) for comparing Kaplan-Meier curves between PM and IM/NM showed a *P* value of *P* = 0·87 for PFS/DFS. *DFS*, disease-free survival; *IM*, intermediate metaboliser; *NM*, normal metaboliser; *N*, number of patients; *PM*, poor metaboliser; *PFS*, progression-free survival; *UGT1A1*, Uridine Diphosphate Glucuronosyltransferase 1A1.

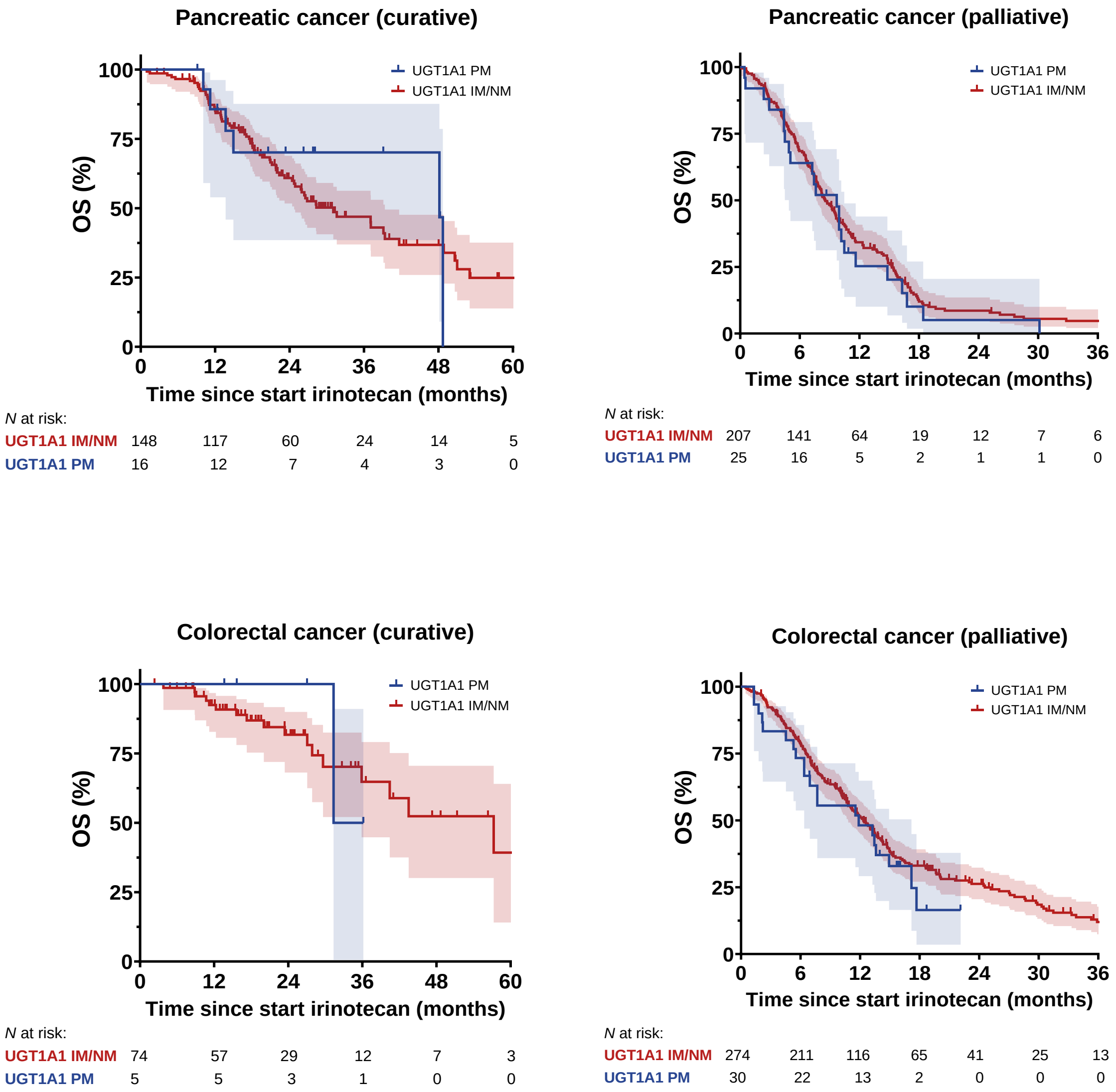

Figure S4. Kaplan-Meier plots for OS of dose-reduced UGT1A1 PM patients and fully-dosed IM/NM patients – secondary analysis stratified by tumour type and treatment setting

Censoring is indicated by tick marks. The shaded area represents the 95% CI. A stratified log-rank test (stratified by tumour type) for comparing Kaplan-Meier curves between PM and IM/NM showed a *P* value of *P* = 0·46 for OS. *IM*, intermediate metaboliser; *NM*, normal metaboliser; *N*, number of patients; *OS*, overall survival; *PM*, poor metaboliser; *UGT1A1*, Uridine Diphosphate Glucuronosyltransferase 1A1.

Table S9. DFS, PFS and OS in dose-reduced UGT1A1 PM patients vs fully-dosed IM/NM patients – secondary analysis stratified by tumour type and treatment setting

**Groups**	***N*[a]**	**DFS events, *N* (%)**	**Median DFS, months**	**95% CI, months**	***N***	**Deceased, *N* (%)**	**Median OS, months**	**95% CI, months**
Colorectal – curative								
IM/NM	74	32 (43·2)	23·4	16·4-30·5	74	17 (23·0)	57·3	34·0-80·6
PM	5	3 (60·0)	14·8	7·6-21·9	5	1 (20·0)	31·3	NA
Pancreatic – curative								
IM/NM	148	92 (62·2)	19·5	16·4-22·7	148	71 (48·0)	31·0	21·2-40·9
PM	16	9 (56·2)	15·3	1·2-29·3	16	6 (37·5)	48·2	28·5-67·8
**Groups**	***N*[a]**	**PFS events, *N* (%)**	**Median PFS, months**	**95% CI, months**	***N***	**Deceased, *N* (%)**	**Median OS, months**	**95% CI, months**
Colorectal – palliative								
IM/NM	274	259 (94·5)	4·4	3·6-5·2	274	205 (74·8)	12·4	10·8-13·9
PM	30	28 (93·3)	5·4	2·7-8·1	30	21 (70·0)	11·9	2·7-21·1
Pancreatic – palliative								
IM/NM	206	202 (98·1)	4·9	4·2-5·7	207	183 (88·4)	8·6	7·4-9·7
PM	25	24 (96·0)	5·1	3·1-7·1	25	23 (92·0)	9·7	6·8-12·7

CI, confidence interval; *DFS,* disease-free survival, *HR,* hazard ratio; *IM,* intermediate metaboliser; *NA,* not applicable; *NM,* normal metaboliser; *N,* number of patients; *OS,* overall survival; *PM,* poor metaboliser; *PFS,* progression-free survival; *UGT1A1,* Uridine Diphosphate Glucuronosyltransferase 1A1.
[a] One palliative IM/NM patient with pancreatic cancer was excluded from PFS analysis because of an unknown disease progression date.

Table S10. HRs of dose-reduced UGT1A1 PM patients vs fully-dosed IM/NM patients – secondary analysis stratified by tumour type and treatment setting

	N[a]	DFS/PFS events, *N* (%)	Univariable analysis DFS/PFS[b]		Multivariable analysis DFS/PFS[c]		*N*	Deceased, *N* (%)	Univariable analysis OS[b]		Multivariable analysis OS[d]	
			**HR (*95%CI*)**	***P* value**	**HR (*95%CI*)**	***P* value**			**HR (*95%CI*)**	***P* value**	**HR (*95%CI*)**	***P* value**
All patients												
IM/NM	702	585 (83·3)	-	-	-	-	703	476 (67·8)	-	-	-	-
PM	76	64 (84·2)	1·02 *(0·79-1·33)*	0·87	1·02 *(0·78-1·32)*	0·91	76	51 (67·1)	1·12 *(0·84-1·50)*	0·42	1·13 *(0·84-1·51)*	0·43

CI, confidence interval; *DFS,* disease-free survival; *HR,* hazard ratio; *IM,* intermediate metaboliser; *NM,* normal metaboliser; *PM,* poor metaboliser; *UGT1A1,* Uridine Diphosphate Glucuronosyltransferase 1A1; *WHO,* World Health Organization.

[a] One IM/NM patient was excluded from DFS/PFS analysis because of an unknown disease progression date.

[b] A stratified Cox model was used for all Cox regression analyses (stratified by tumour type (colorectal/pancreatic) and treatment setting (curative/palliative treatment indication).

[c] Multivariable Cox regression DFS/PFS analysis stratified by tumour type and treatment setting and adjusted for covariates age, WHO performance status, tumour stage, treatment regimen, previous chemotherapy.

[d] Multivariable Cox regression OS analysis stratified by tumour type and treatment setting and adjusted for covariates WHO performance status, tumour stage, treatment regimen, previous chemotherapy.

Table S11. Univariable Cox regression analyses – secondary analysis stratified by tumour type and treatment setting.

**Variables**	**Univariable analysis[a] DFS/PFS**		**Univariable analysis[a] OS**	
	**HR (*95%CI*)**	***P* value**	**HR (*95%CI*)**	***P* value**
**UGT1A1 group**				
IM/NM (reference group)	-	-	-	-
PM	1·02 *(0·79-1·33)*	0·87	1·12 *(0·84-1·50)*	0·42
**Sex**				
Male (reference group)	-	-	-	-
Female	0·98 *(0·84-1·14)*	0·78	1·04 *(0·87-1·24)*	0·66
**Age**				
< 65 years (reference group)	-	-	-	-
≥ 65 years	1·21 *(1·03-1·42)*	0·02	1·14 *(0·96-1·36)*	0·14
**WHO performance status**				
0 – 1 (reference group)	-	-	-	-
2 – 3	1·57 *(1·10-2·24)*	0·01	1·87 *(1·27-2·74)*	0·002
**Ethnicity**				
European (reference group)	-	-	-	-
Other	0·84 *(0·62-1·14)*	0·27	1·05 *(0·76-1·46)*	0·77
**Smoking status**				
Former or Never (reference group)	-	-	-	-
Current	1·05 *(0·84-1·30)*	0·69	1·15 *(0·90-1·46)*	0·26
Unknown	1·11 *(0·73-1·69)*	0·61	1·23 *(0·76-1·97)*	0·40
**Tumour Stage**				
I (reference group)	-	-	-	-
II	1·69 *(1·01-2·83)*	0·04	1·71 *(0·93-3·14)*	0·09
III	1·13 *(0·66-1·94)*	0·66	1·18 *(0·63-2·19)*	0·61
IV	1·81 *(1·02-3·21)*	0·04	2·26 *(1·18-4·31)*	0·01
**Treatment Regimen[d]**				
FOLFIRINOX (180 mg/m^2) (reference group)	-	-	-	-
mFOLFIRINOX (150 mg/m^2)	0·85 *(0·64-1·13)*	0·27	0·81 *(0·60-1·11)*	0·19
Irinotecan monotherapy – low[b]	0·54 *(0·33-0·87)*	0·01	0·68 *(0·40-1·15)*	0·15
Irinotecan monotherapy – high[c]	0·70 *(0·44-1·12)*	0·13	0·71 *(0·43-1·16)*	0·17
FOLFIRI (180 mg/m^2)	0·71 *(0·47-1·06)*	0·10	0·62 *(0·40-0·96)*	0·03
FOLFIRI + B/C (180 mg/m^2)	0·34 *(0·21-0·54)*	<0·001	0·48 *(0·29-0·79)*	0·004
FOLFOXIRI (165 mg/m^2)	0·27 *(0·14-0·51)*	<0·001	0·37 *(0·16-0·81)*	0·01
FOLFOXIRI + B (165 mg/m^2)	0·22 *(0·14-0·37)*	<0·001	0·25 *(0·14-0·46)*	<0·001
**Previous surgery**				
No (reference group)	-	-	-	-
Yes	1·02 *(0·85-1·22)*	0·83	0·82 *(0·66-1·01)*	0·06
**Previous radiotherapy**				
No (reference group)	-	-	-	-
Yes	1·16 *(0·95-1·42)*	0·16	1·10 *(0·88-1·39)*	0·41
**Previous chemotherapy**				
No (reference group)	-	-	-	-
Yes	1·57 *(1·28-1·92)*	<0·001	1·32 *(1·03-1·69)*	0·03

CI, confidence interval; *DFS,* disease-free survival; *HR,* hazard ratio; *IM,* intermediate metaboliser; *NM,* normal metaboliser; *PM,* poor metaboliser; *UGT1A1,* Uridine Diphosphate Glucuronosyltransferase 1A1; *WHO,* World Health Organization.

[a] A stratified Cox model was used for all Cox regression analyses (stratified by tumour type (colorectal/pancreatic) and treatment setting (curative/palliative treatment indication).

[b] Irinotecan monotherapy low dose: 180 mg/m^2 or 450 mg flat dose either q2w or q3w

[c] Irinotecan monotherapy high dose: 350 mg/m^2 or 600 mg flat dose either q2w or q3w

[d] Other treatment regimens was not included as a covariate because it was found to be a constant or linearly dependent covariate.

Table S12. HRs of dose-reduced UGT1A1 PM patients vs fully-dosed IM/NM patients – subgroup analyses per tumour type and treatment setting.

**Groups**	***N*[a]**	**Univariable analysis DFS**		***N***	**Univariable analysis OS**	
		**HR *(95%CI)***	***P* value**		**HR *(95%CI)***	***P* value**
Colorectal – curative						
IM/NM	74	-	-	74	-	-
PM	5	1·38 *(0·42-4·52)*	0·60	5	0·80 *(0·11-6·08)*	0·83
Pancreatic – curative						
IM/NM	148	-	-	148	-	-
PM	16	0·94 *(0·47-1·87)*	0·86	16	0·80 *(0·35-1·84)*	0·59
**Groups**	***N*[a]**	**Univariable analysis PFS**		***N***	**Univariable analysis OS**	
		**HR *(95%CI)***	***P* value**		**HR *(95%CI)***	***P* value**
Colorectal – palliative						
IM/NM	274	-	-	274	-	-
PM	30	1·09 *(0·74-1·62)*	0·65	30	1·24 *(0·78-1·94)*	0·36
Pancreatic – palliative						
IM/NM	206	-	-	207	-	-
PM	25	0·95 *(0·62-1·45)*	0·80	25	1·16 *(0·75-1·80)*	0·50

CI, confidence interval; *DFS,* disease-free survival, *HR,* hazard ratio; *IM,* intermediate metaboliser; *NA,* not applicable; *NM,* normal metaboliser; *N,* number of patients; *OS,* overall survival; *PM,* poor metaboliser; *PFS,* progression-free survival; *UGT1A1,* Uridine Diphosphate Glucuronosyltransferase 1A1.

[a] One palliative IM/NM patient with pancreatic cancer was excluded from PFS analysis because of an unknown disease progression date.

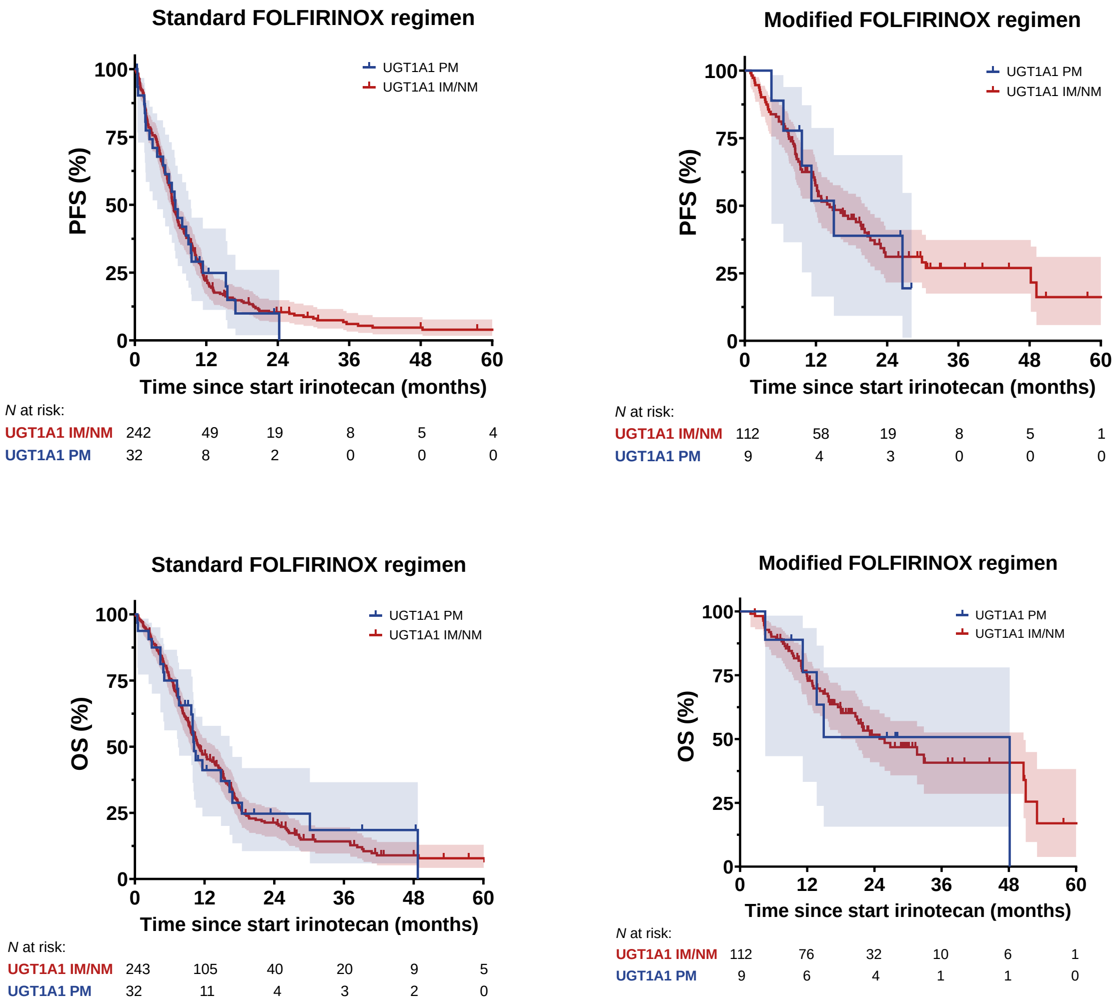

Figure S5. Kaplan-Meier plots for PFS and OS of dose-reduced UGT1A1 PM patients and fully-dosed IM/NM patients – subgroup analysis in patients with pancreatic cancer with standard or modified FOLFIRINOX

Censoring is indicated by tick marks. The shaded area represents the 95% CI. A log-rank tests for comparing Kaplan-Meier curves between PM and IM/NM with standard FOLFIRINOX showed a *P* value of *P* = 0·54 for PFS and *P* = 0·82 for OS. A log-rank tests for comparing Kaplan-Meier curves between PM and IM/NM with standard FOLFIRINOX showed a *P* value of *P* = 0·97 for PFS and *P* = 0·79 for OS. *IM*, intermediate metaboliser; *NM*, normal metaboliser; *N*, number of patients; *OS*, overall survival; *PFS*, progression-free survival; *PM*, poor metaboliser; *UGT1A1*, Uridine Diphosphate Glucuronosyltransferase 1A1.

Table S13. PFS and OS in dose-reduced UGT1A1 PM patients vs fully-dosed IM/NM patients – subgroup analysis in patients with pancreatic cancer with standard or modified FOLFIRINOX

**Groups**	***N*[a]**	**PFS events, *N* (%)**	**Median PFS, months**	**95% CI, months**	***N***	**Deceased, *N* (%)**	**Median OS, months**	**95% CI, months**
Standard FOLFIRINOX								
IM/NM	242	222 (91·7)	6·4	5·8-7·0	243	199 (81·9)	11·0	9·0-13·0
PM	32	27 (84·4)	7·2	3·9-10·5	32	24 (75·0)	10·2	9·3-11·1
Modified FOLFIRINOX								
IM/NM	112	72 (64·3)	14·3	8·9-19·7	112	55 (49·1)	25·8	16·7-34·9
PM	9	6 (66·7)	15·0	8·0-22·1	9	5 (55·6)	48·2	NA

CI, confidence interval; *HR,* hazard ratio; *IM,* intermediate metaboliser; *NA,* not applicable; *NM,* normal metaboliser; *N,* number of patients; *OS,* overall survival; *PM,* poor metaboliser; *PFS*, progression-free survival; *UGT1A1,* Uridine Diphosphate Glucuronosyltransferase 1A1.

[a] One IM/NM patient with standard FOLFIRINOX was excluded from PFS analysis because of an unknown disease progression date.

Table S14. HRs of dose-reduced UGT1A1 PM patients vs fully-dosed IM/NM patients – subgroup analysis in patients with pancreatic cancer with standard or modified FOLFIRINOX

	***N*[a]**	**PFS events, *N* (%)**	**Univariable analysis PFS**		***N***	**Deceased, *N* (%)**	**Univariable analysis OS**	
			**HR (*95%CI*)**	***P* value**			**HR (*95%CI*)**	***P* value**
Standard FOLFIRINOX								
IM/NM	242	222 (91·7)	-	-	243	199 (81·9)	-	-
PM	32	27 (84·4)	0·88 *(0·59-1·32)*	0·55	32	24 (75·0)	0·95 *(0·62-1·46)*	0·82
Modified FOLFIRINOX								
IM/NM	112	72 (64·3)	-	-	112	55 (49·1)	-	-
PM	9	6 (66·7)	1·01 *(0·44-2·34)*	0·97	9	5 (55·6)	1·13 *(0·45-2·84)*	0·79

CI, confidence interval; *HR,* hazard ratio; *IM,* intermediate metaboliser; *NM,* normal metaboliser; *PM,* poor metaboliser; *UGT1A1,* Uridine Diphosphate Glucuronosyltransferase 1A1; *WHO,* World Health Organization.

[a] One IM/NM patient with standard FOLFIRINOX was excluded from PFS analysis because of an unknown disease progression date.

Table S15. Toxicity outcomes – subgroup analysis in patients with pancreatic cancer with standard or modified FOLFIRINOX

**Standard FOLFIRINOX**	**UGT1A1 PM *N* = 32**	**UGT1A1 IM/NM *N* = 243**	***P* value (Chi-square Test)**
Overall grade ≥ 3 toxicity in the first three cycles, *N* (%)	8 (25·0)	104 (42·8)	0·05
**Modified FOLFIRINOX**	**UGT1A1 PM *N* = 9**	**UGT1A1 IM/NM *N* = 112**	***P* value (Fisher's Exact Test)**
Overall grade ≥ 3 toxicity in the first three cycles, *N* (%)	3 (33·3)	41 (36·6)	1·00

IM, intermediate metaboliser; *NM,* normal metaboliser; *PM,* poor metaboliser.

Supplementary methods

UGT1A1 genotyping and classification of genotypes

Prior to start of treatment with irinotecan, all patients from all centres were genotyped for genetic variant *UGT1A1**28 ((TA)7TAA). Additional genotyping for variants *UGT1A1**93 (-3156G>A), *UGT1A1**6 (c.211G>A), *UGT1A1**36 ((TA)5TAA), *UGT1A1**37 ((TA)8TAA), and *UGT1A1**80 (-364C>T) varied across centres and time periods.

DNA for genotyping was isolated from 200µl of whole EDTA blood that was obtained prior to start of therapy. Genotyping was conducted using validated real-time PCR reactions and included appropriate wild type, heterozygous and homozygous controls in every run. Genotyping was performed twice weekly to minimise delay in start of treatment.

UGT1A1 genotyping was conducted in the local clinical laboratories of three of the participating centres: Erasmus Medical Centre performed genotyping for patients from the Erasmus Medical Centre, Maasstad Hospital, and Reinier de Graaf Gasthuis Hospital; Catharina Hospital performed genotyping for patients from the Catharina Hospital and Jeroen Bosch Hospital; Leiden University Medical Centre performed genotyping for patients from the Leiden University Medical Centre. Testing panels by the Catharina Hospital generally included *28, *93 and *37 between 2017 and 2023, and included *28, *93, *6, and *80 from the end of 2023 onwards. Testing panels by the Erasmus Medical Centre generally included *28 and *93 between 2017 and 2022, included *28, *36, *37 between halfway 2022 and 2023, and included *28, *36, *37, and *6 from 2024 onwards. Testing panels by the Leiden University Medical Centre included *28 and *93 between 2017 and 2021, and included only *28 from 2022 onwards.

As described in the main article, genotypes were converted to phenotypes following the DPWG guideline and ClinPGx.org nomenclature, as described in the table below. *UGT1A1**36 is thought to cause increased functionality of the UGT1A1 enzyme. However, due to insufficient evidence, carriers of this variant are categorised as 'normal metaboliser' when no other relevant *UGT1A1* variant is present.

***UGT1A1* genotype**	**Predicted UGT1A1 phenotype**
*1/*1	Normal metaboliser
*1/*6	Intermediate metaboliser
*1/*28	Intermediate metaboliser
*1/*36	Normal metaboliser
*1/*37	Intermediate metaboliser
*1/*80+*28	Intermediate metaboliser
*1/*80+*37	Intermediate metaboliser
*1/*93+*28	Intermediate metaboliser
*1/*93+*37	Intermediate metaboliser
*6/*6	Poor metaboliser
*6/*28	Poor metaboliser
*6/*36	Poor metaboliser
*6/*37	Poor metaboliser
*6/*80+*28	Poor metaboliser
*6/*80+*37	Poor metaboliser
*6/*93+*28	Poor metaboliser
*6/*93+*37	Poor metaboliser
*28/*28	Poor metaboliser
*28/*36	Poor metaboliser
*28/*37	Poor metaboliser
*28/*80+*28	Poor metaboliser
*28/*80+*37	Poor metaboliser
*28/*93+*28	Poor metaboliser
*28/*93+*37	Poor metaboliser
*36/*36	Normal metaboliser
*36/*37	Intermediate metaboliser
*36/*80+*28	Intermediate metaboliser
*36/*80+*37	Intermediate metaboliser

*36/*93+*28	Intermediate metaboliser
*36/*93+*37	Intermediate metaboliser
*37/*37	Poor metaboliser
*37/*80+*28	Poor metaboliser
*37/*80+*37	Poor metaboliser
*37/*93+*28	Poor metaboliser
*37/*93+*37	Poor metaboliser
*80+*28/*80+*28	Poor metaboliser
*80+*28/*80+*37	Poor metaboliser
*80+*37/*80+*37	Poor metaboliser
*93+*28/*93+*28	Poor metaboliser
*93+*28/*93+*37	Poor metaboliser
*93+*37/*93+*37	Poor metaboliser

Secondary analysis in all *UGT1A1* genotyped patients treated with systemic irinotecan

A secondary analysis was conducted in all genotyped patients treated with systemic irinotecan, regardless of their irinotecan dosage in cycle 1, consisting of 889 IM/NMs and 100 PMs. This analysis also included PMs who started at a >80% dose intensity, PMs who started at a <60% dose intensity, and IM/NMs who started at a dose intensity <90%. An additional 9 PMs who unintentionally started at a >80% irinotecan dose intensity, 15 PMs who started at a <60% irinotecan dose intensity, and 186 IM/NMs who started at a dose intensity <90% were included in this analysis, all of whom were excluded from the primary analysis.

Survival analyses were performed in the same manner as for the primary analysis. Endpoints PFS and OS were compared between all PMs and all IM/NMs. PFS was a composite endpoint, and was defined as the time between initiation of irinotecan treatment and the first signs of disease progression by either clinical signs or radiological imaging (in accordance with RECIST 1.1), or death from any cause, whichever came first. Disease recurrence was considered a progression event in patients who received (neo)adjuvant irinotecan. OS was defined as the time between initiation of irinotecan treatment and death from any cause. Patients who did not experience disease progression or death before the end of follow-up were censored at the last date known to be alive. PFS and OS survival curves were generated using the Kaplan-Meier method. Median PFS and OS were compared between UGT1A1 PMs and IM/NMs using Kaplan-Meier estimates and a stratified log-rank test for equivalence of survival curves (stratified by tumour type). Univariable Cox regression analyses were performed to assess the association between UGT1A1 groups (PM *vs* IM/NM) and endpoints PFS and OS. In addition, the association between relevant covariates and PFS and OS was tested in univariable Cox regressions. Multivariable Cox regression analyses were performed for PM *vs* IM/NM groups, adjusted for covariates with $P < 0{\cdot}10$ in univariable Cox regression analysis. Hazard ratios (HRs) and their corresponding 95% confidence intervals (CIs) were calculated. All Cox regression analyses were conducted as stratified Cox-regressions by tumour type.

Patient and treatment characteristics were compared between UGT1A1 groups (PM *vs* IM/NM) using descriptive statistics. For the toxicity analysis, Chi-square Test or Fisher's Exact Test was used to compare frequencies of overall severe (CTCAE grade ≥ 3) toxicity between UGT1A1 groups (PM *vs* IM/NM).

Secondary analysis with stratification by tumour type and treatment setting

Another secondary analysis was conducted in the patient cohort of the primary analysis, that is, 76 PMs and 703 IM/NMs, all of whom received an initial irinotecan dose according to their *UGT1A1* genotype. Additional stratification by treatment setting (curative/palliative) was included besides tumour type (colorectal/pancreatic) in this secondary survival analysis. Patients were considered to be curative if they received adjuvant or neo-adjuvant irinotecan treatment as well as curative surgery. All other patients were considered to be palliative. As a result, four strata were created: curative colorectal cancer, curative pancreatic cancer, palliative colorectal cancer, and palliative pancreatic cancer.

Endpoints DFS, PFS and OS were compared between 30% dose-reduced PMs and fully-dosed IM/NMs. DFS and PFS were composite endpoints. PFS was defined as the time between initiation of irinotecan treatment and the first

signs of disease progression by either clinical signs or radiological imaging (in accordance with RECIST 1.1), or death from any cause, whichever came first. DFS was defined as the time between initiation of irinotecan treatment and the first signs of disease recurrence by either clinical signs or radiological imaging, or death from any cause, whichever came first. OS was defined as the time between initiation of irinotecan treatment and death from any cause. Patients who did not experience disease recurrence, progression or death before the end of follow-up were censored at the last date known to be alive.

DFS curves and median DFS estimates were generated for the curative colorectal cancer and curative pancreatic cancer groups using the Kaplan-Meier method. PFS curves and median PFS estimates were generated for the palliative colorectal cancer and palliative pancreatic cancer groups using the Kaplan-Meier method. OS curves and median OS estimates were generated for all groups using the Kaplan-Meier method. A stratified log-rank test for equivalence of survival curves (stratified by tumour type and treatment setting) was used to compare DFS, PFS and OS between dose-reduced PMs and fully-dosed IM/NMs. For the stratified log-rank test, DFS and PFS were combined into one composite endpoint 'DFS/PFS' (the time between initiation of irinotecan treatment and the first signs of disease recurrence or progression, by either clinical signs or radiological imaging, or death from any cause, whichever came first).

All Cox regression analyses in this secondary analysis were conducted as stratified Cox-regressions by tumour type and treatment setting. The stratified Cox regression takes into account different baseline hazards for each stratum, while assuming a common HR. In other words, each subgroup (curative colorectal cancer, curative pancreatic cancer, palliative colorectal cancer, and palliative pancreatic cancer) was assigned its own baseline hazard function to adjust for differences in the inherent risk of the event (i.e. disease progression or death) of each subgroup, while the overall effect of PM *vs* IM/NM on survival was summarised across all subgroups. Univariable Cox regression analyses were performed to assess the association between UGT1A1 groups (PM *vs* IM/NM) and endpoints DFS/PFS and OS. In addition, the association between relevant covariates (other than tumour type and treatment setting) and DFS/PFS and OS was tested in univariable Cox regressions. Multivariable Cox regression analyses were performed for PM *vs* IM/NM groups, additionally adjusted for covariates (other than tumour type and treatment setting) with $P < 0{\cdot}10$ in univariable Cox regression analysis. HRs and their corresponding 95% CIs were calculated.

<u>Cox regression analyses for subgroups per tumour type and treatment setting</u>

In addition to the secondary analyses using stratification by tumor type and treatment setting, we conducted separate univariable Cox regressions for each of the four subgroups defined by tumor type and treatment setting: curative colorectal cancer, curative pancreatic cancer, palliative colorectal cancer, and palliative pancreatic cancer. These subgroup analyses were performed to assess the effect of *UGT1A1* group (PM *vs* IM/NM) on DFS, PFS, and OS. While the stratified Cox regression analysis assumes a common hazard ratio across strata, the univariable Cox regressions for each subgroup estimate the effect of PM *vs* IM/NM across different strata. which can help assess effect modification. This approach allows for the assessment of effect modification, to directly evaluate whether the observed PM *vs* IM/NM effect is consistent across different tumor types and treatment settings. Given the exploratory nature of these subgroup analyses and the relatively small sample sizes within each subgroup, no multivariable Cox regressions were performed. HRs and their corresponding 95% CIs were calculated for each subgroup analysis.

<u>Subgroup analysis for standard FOLFIRINOX and modified FOLFIRINOX</u>

The DPWG guideline on *UGT1A1* genotype-guided irinotecan dosing concludes that the risk of severe irinotecan-related toxicity is elevated at both high dose regimens (≥180 mg/m^2 irinotecan) and low dose regimens (<150 mg/m^2 irinotecan) based on two meta-analyses, and therefore recommends *UGT1A1* genotype-guided dosing for all irinotecan-containing regimens. To gain insight in the generalisability and impact of *UGT1A1*-guided dose reductions across high and low dose irinotecan regimens, we conducted exploratory subgroup analyses in a subset of the primary analysis patient cohort. These subgroup analyses were conducted in 275 patients with pancreatic cancer and standard FOLFIRINOX treatment (containing 180 mg/m^2 irinotecan), and 121 patients with pancreatic cancer and modified FOLFIRINOX treatment (containing 150 mg/m^2 irinotecan), all of whom received an initial irinotecan dose according to their *UGT1A1* genotype.

Survival analyses were performed in the same manner as for the primary analysis for both subgroups (standard FOLFIRINOX subgroup and modified FOLFIRINOX subgroup). Endpoints PFS and OS were compared between all PMs and all IM/NMs. PFS was a composite endpoint, and was defined as the time between initiation of irinotecan treatment and the first signs of disease progression by either clinical signs or radiological imaging (in accordance with RECIST 1.1), or death from any cause, whichever came first. Disease recurrence was considered a progression event in patients who received (neo)adjuvant irinotecan. OS was defined as the time between initiation of irinotecan treatment and death from any cause. Patients who did not experience disease progression or death before the end of follow-up were censored at the last date known to be alive. PFS and OS survival curves were generated using the Kaplan-Meier method. Median PFS and OS were compared between UGT1A1 PMs and IM/NMs using Kaplan-Meier estimates and a log-rank test for equivalence of survival curves. Univariable Cox regression analyses were performed to assess the association between UGT1A1 groups (PM *vs* IM/NM) and endpoints PFS and OS. Due to the exploratory nature of this analysis and low sample size, no multivariable Cox regression analyses were performed this subgroup analysis. HRs and their corresponding 95% CIs were calculated. For the toxicity analysis, Chi-square Test or Fisher's Exact Test was used to compare frequencies of overall severe (CTCAE grade ≥ 3) toxicity between UGT1A1 groups (PM *vs* IM/NM).

Study protocol non-WMO research

<table>
<tr><th colspan="4">STUDY DETAILS</th></tr>
<tr><td>Title study</td><td colspan="3">Survival Analysis of Cancer Patients with UGT1A1 Variant Alleles and Dose-Individualized Irinotecan Therapy and Other Irinotecan-related Sub-analyses</td></tr>
<tr><td>Acronym</td><td colspan="3">IRI-DATABASE</td></tr>
<tr><td>Date / version</td><td colspan="3">29-04-2024, version 2.1</td></tr>
<tr><td>Type of study</td><td colspan="3">Retrospective study</td></tr>
<tr><td>Sponsor</td><td colspan="3">Department of Clinical Pharmacy
Catharina Hospital Eindhoven
Michelangolaan 2, 5623 EJ,
Eindhoven, the Netherlands</td></tr>
<tr><td rowspan="7">Study team</td><td colspan="2">Name + affiliation</td><td>Role</td></tr>
<tr><td>1</td><td>dr. M. J. Deenen, PharmD, PhD
Department of Clinical Pharmacy
Catharina Hospital Eindhoven
Michelangolaan 2, 5623 EJ,
Eindhoven, the Netherlands</td><td>Principal investigator</td></tr>
<tr><td>2</td><td>S.L.J. Peeters, PharmD, PhD candidate
Department of Clinical Pharmacy
Catharina Hospital Eindhoven
Michelangolaan 2, 5623 EJ,
Eindhoven, the Netherlands

Department of Clinical Pharmacy and Toxicology
Leiden University Medical Centre
Albinusdreef 2, 2333 ZG
Leiden, the Netherlands</td><td>Coordinating investigator</td></tr>
<tr><td>3</td><td>dr. A.M.J. Thijs, MD, PhD
Department of Medical Oncology
Catharina Hospital Eindhoven
Michelangolaan 2, 5623 EJ,
Eindhoven, the Netherlands</td><td>Local co-investigator</td></tr>
<tr><td>4</td><td>T.M.M. Böhm, PharmD, PhD candidate
Department of Clinical Pharmacy
Catharina Hospital Eindhoven
Michelangolaan 2, 5623 EJ,
Eindhoven, the Netherlands</td><td>Local co-investigator</td></tr>
<tr><td>5</td><td>Prof. dr. R.H.J Mathijssen, MD, PhD
Department of Medical Oncology
Erasmus University Medical Centre
Groene Hilledijk 301, 3075 EA Rotterdam, the Netherlands</td><td>Local principal investigator</td></tr>
<tr><td>6</td><td>N. Heersche, MD, PhD candidate
Department of Medical Oncology and Clinical Chemistry
Erasmus University Medical Centre</td><td>Coordinating investigator</td></tr>
</table>

		Groene Hilledijk 301, 3075 EA Rotterdam, the Netherlands	
	7	**Prof. dr. A.J. Gelderblom, MD, PhD**<br>Department of Medical Oncology<br>Leiden University Medical Centre<br>Albinusdreef 2, 2333 ZG<br>Leiden, the Netherlands	Local principal investigator
	8	**Prof. dr. H.J. Guchelaar, PharmD, PhD**<br>Department of Clinical Pharmacy and Toxicology<br>Leiden University Medical Centre<br>Albinusdreef 2, 2333 ZG<br>Leiden, the Netherlands	Local co-investigator
	9	**Dr. S. Böhringer, MD, PhD**<br>Department of Biomedical Data Sciences<br>Leiden University Medical Centre<br>Albinusdreef 2, 2333 ZG<br>Leiden, the Netherlands	Statistician
	10	**Dr. M.L. Wumkes, MD, PhD**<br>Department of Medical Oncology<br>Jeroen Bosch Hospital<br>Henri Dunanstraat 1, 5223 GZ<br>‘s Hertogenbosch, the Netherlands	Local principal investigator
	11	**Dr. B.C.M. Haberkorn, MD, PhD**<br>Department of Medical Oncology<br>Maasstad Hospital<br>Maasstadweg 21, 3079 DZ<br>Rotterdam, the Netherlands	Local principal investigator
	12	**Dr. A.J. Verschoor**<br>Department of Medical Oncology<br>Reinier de Graaf Gasthuis Hospital<br>Reinier de Graafweg 5, 2625 AD<br>Delft, the Netherlands	Local principal investigator

**Submitter**	**Sofía Peeters, PharmD, PhD Candidate**<br>Department of Clinical Pharmacy<br>Catharina Hospital Eindhoven<br>Michelangolaan 2, 5623 EJ,<br>Eindhoven, the Netherlands<br>sofia.peeters@catharinaziekenhuis.nl	**Signature + date**<br>29-04-2024<br>Sofia
**Principal Investigator**	**dr. Maarten Deenen, PharmD, PhD**<br>Department of Clinical Pharmacy<br>Catharina Hospital Eindhoven<br>Michelangolaan 2, 5623 EJ,<br>Eindhoven, the Netherlands<br>Maarten.deenen@catharinaziekenhuis.nl	**Signature + date**<br>17-06-2024
**Sponsor**	**dr. R. ten Broeke, PharmD, PhD**<br>Department of Clinical Pharmacy<br>Catharina Hospital Eindhoven<br>Michelangolaan 2, 5623 EJ,<br>Eindhoven, the Netherlands	**Signature + date**<br>17-06-2024

CONTENTS

SYNOPSIS 4
1 INTRODUCTION 5
2 RESEARCH QUESTION / RESEARCH AIM 9
3 METHODS 10
3.1 Mono- or multicentre study 10
3.2 Study design 10
3.3 Intervention 10
3.4 Study duration 10
3.5 Recruitment and selection of study population 10
3.6 Data collection: variables and methods 13
3.7 Data-analysis 19
4 ETHICAL CONSIDERATIONS 23
4.1 Niet WMO verklaring 23
4.2 Compensation for study subjects 23
4.3 Informed consent study subjects 23
5 DATAMANAGEMENT & PRIVACY 25
5.1 5.1 Data storage, security and access during research 25
5.2 Data processing 26
5.3 Data delen 27
5.4 How long is data stored? 28
6 VALORISATION AND PUBLICATION 29
6.1 Valorisation 29
6.2 Publication 29
7 REFERENCES 30

SYNOPSIS

Rationale: Irinotecan treatment is commonly used in colorectal and pancreatic cancer, but is often complicated by its severe toxicity. Polymorphisms in the *UGT1A1* gene are known to play a role in the metabolism of irinotecan and its active metabolite, SN-38. Homozygous *UGT1A1* variant allele carriers, also known as poor metabolizers (PMs), have a reduced UGT1A1 enzyme activity that leads to elevated SN-38 blood plasma concentrations. Consequently, UGT1A1 PMs are at a higher risk for severe toxicity when treated with standard irinotecan doses. Previous research has demonstrated that a *UGT1A1*-genotyped guided dosing reduces the incidence of severe irinotecan-related toxicity, and the Dutch Pharmacogenetics Working Group (DPWG) guideline now recommends pretreatment *UGT1A1* genotyping and initial dose irinotecan reductions of 30% in all patient who are PM for UGT1A1. However, *UGT1A1* genotype-guided dosing of Irinotecan has yet to be implemented widely in clinical practice, and is currently hindered by implementation barriers such as the concern for reduced efficacy upon irinotecan dose reduction. Therefore, studies on long-term clinical outcomes of *UGT1A1* genotype-guided dosing of Irinotecan are needed. Furthermore, a significant proportion of intermediate and normal metabolizers (IM and EM) of UGT1A1 still experience severe irinotecan-related toxicity. Thus, exploration of new prognostic and/or predictive factors that can further individualize irinotecan treatment to improve its safety and efficacy are needed.

Objectives: The primary objective of this study is to determine the effect of an initial 70% irinotecan dose intensity in UGT1A1 poor metabolizers (PM) compared to a standard irinotecan dose (100% dose intensity) in UGT1A1 intermediate metabolizers (IM) and normal metabolizers (NM) on efficacy outcomes (progression free survival (PFS), overall survival (OS)), with a non-inferiority based approach. Secondary objectives include: to investigate the correlation of patient- and treatment characteristics (e.g. sex, age, tumor type, stadium, treatment schedule, dose modifications, G-CSF use, PPI, corticosteroids, and antibiotics use, body composition parameters…) on treatment outcomes (PFS, OS and irinotecan-induced toxicity); to identify novel germline DNA variant alleles associated with irinotecan toxicity and/or efficacy; to determine and describe the incidence of irinotecan-induced grade 1-5 toxicity, toxicity-related hospital admissions, treatment delay and early treatment withdrawal.

Study design: This study is a retrospective multicenter observational cohort study. Data will be collected through electronic patient records of participating centers (CZE, EMC, LUMC, JBZ, RdGG Delft, Maasstad).

Study population: The study population consists of the IRI28-study cohort (NL59765.100.17 / CZE-2017.43) and a new expansion cohort. New patients since closing of the IRI28 study that have been treated with systemic irinotecan will be included in the expansion cohort will be included. Patients with unknown *UGT1A1* status are excluded. No informed consent will be asked. Patients that have previously stated their data may not be used for (future) research (through opt-out or those from the IRI28-study that stated so on the ICF) will be excluded in this study. Patients that have received irinotecan-treatment in the period of August 2017 to April 2024 will be included and followed-up. All patients have been treated according to standard-of-care national/local oncology guidelines.

Main study parameters/endpoints: The primary endpoint for this study is PFS. Progression is defined as death from any cause, radiological progression confirmed by RECIST 1.1 criteria, progression that is treated by treating physician (radiotherapy for oligometastases or switch to other line of treatment). Secondary endpoints include OS, severe (grade ≥ 3) irinotecan-related toxicity, and patient-and treatment characteristics.

1 INTRODUCTION

Background

Irinotecan is a chemotherapeutic agent used in the treatment of various types of cancer. For the last three decades, it has played a major role in the treatment of mainly solid gastrointestinal cancers, specifically in regimes like FOLFIRI, FOLFIRINOX and FOLFOXIRI for the treatment of advanced and metastatic colorectal and pancreatic cancer [1-6]. Treatment with irinotecan is often complicated by adverse drug reactions (ADRs) such as severe febrile neutropenia and severe diarrhea, with up to 40-50% of patients experiencing severe grade ≥ 3 toxicity. This may result in treatment delay and/or discontinuation, reduced quality of life and ultimately increased morbidity and even mortality [7]. Several studies have demonstrated variations in the *UGT1A1* gene (encoding the UDP-glucuronosyltransferase 1A1 (UGT1A1) enzyme) are associated with the risk of severe irinotecan-induced toxicity [8-11]. This highlights the importance of pharmacogenetics in irinotecan treatment.

Irinotecan is a prodrug that is activated in the liver and blood via carboxylesterases to its active metabolite SN-38 (Figure 1, Figure 2), which is 100–1000 times more potent that its parent drug. Inactivation of SN-38 is regulated by UGT1A1, an enzyme found in the liver and intestines (Figure 1, Figure 2) [7,12]. Certain variations in the *UGT1A1* gene can affect the drug's metabolism by reducing UGT1A1 enzyme activity, and consequently increase the risk of severe adverse effects. Clinically relevant polymorphisms for *UGT1A1* include *UGT1A1**28 and *UGT1A1**6. The prevalence of the *UGT1A1**28 allele in Europe ranges from 22 to 39% while the *UGT1A1**6 allele is more commonly seen in Asian populations with a prevalence of 13 to 23% [13,14]. Patients who are homozygous variant carriers for these alleles categorized as poor metabolizers (PM) (comprising 10-17% of patients [13]), which translates to having a reduced UGT1A1 activity and leads to elevated SN-38 blood plasma concentrations [7,12]. Consequently, UGT1A1 PMs are at a higher risk for severe toxicity when treated with standard irinotecan doses. [15].

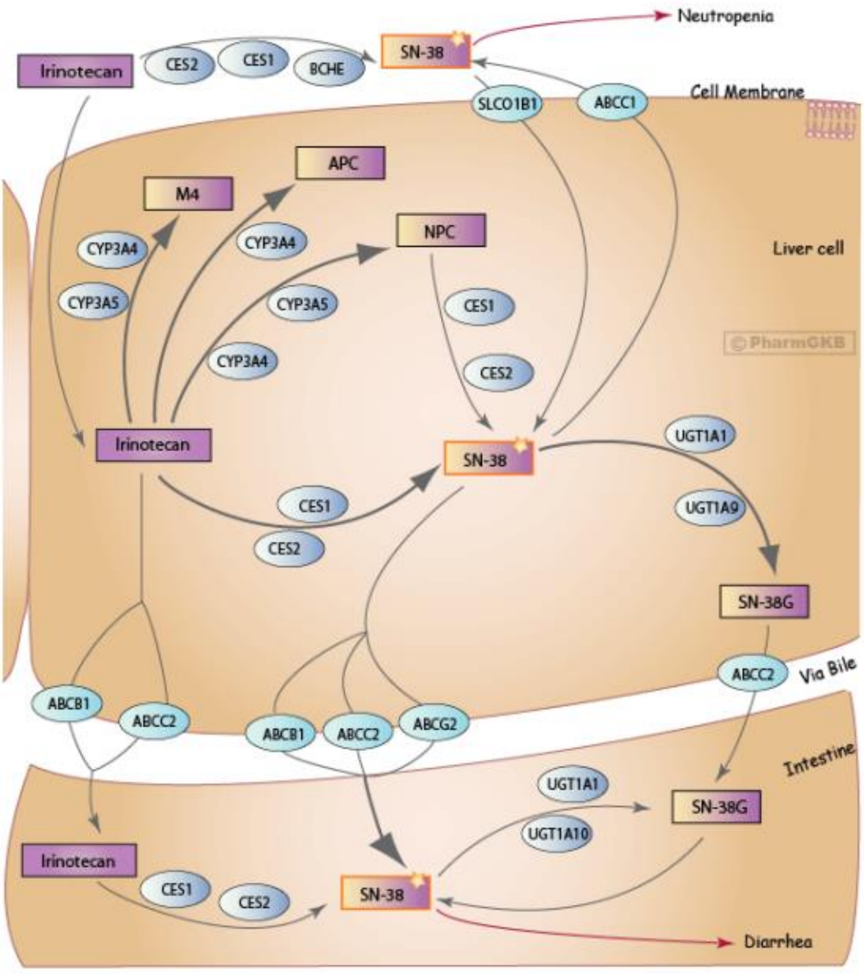

Figure 1: Pathway of irinotecan metabolism and transport in the hepatocyte. *https://www.pharmgkb.org/pathway/PA2001*.

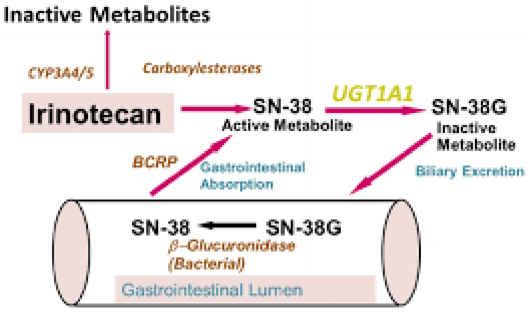

Figure 2: Simplified metabolism of irinotecan [16].

We conducted a prospective safety and feasibility trial (Hulshof et al. (2022)) on *UGT1A1* genotype-guided dosing of irinotecan in UGT1A1 PMs [17]. Patients were genotyped for relevant *UGT1A1* variants pre-treatment to guide irinotecan dosage based on their UGT1A1 phenotype. PMs received an initial dose intensity of 70% irinotecan while intermediate metabolizers (IM) and normal metabolizers (NM) received the standard dose intensity (100%). A 30% reduction of the standard dose resulted in significantly safer treatment in UGT1A1 PMs. We demonstrated that *UGT1A1* genotype-guided dosing significantly reduced the incidence of febrile neutropenia by four-fold, and significantly reduced the incidence grade 3-4 toxicity and chemotherapy-related hospital admission in UGT1A1 PMs. Based on these findings, the Dutch Pharmacogenetics Working Group (DPWG) reevaluated and adjusted their guidelines for irinotecan. The new DPWG guideline recommends pretreatment *UGT1A1* genotyping and initial dose irinotecan reductions of 30% in all patient who are PM for UGT1A1 [14]. This is in line with other European

guidelines such as the French National Network of Pharmacogenetics (RNPGx) guideline and the Italian Association of Medical Oncology (AIOM) guideline [18,19]. Since the publication of our study results, *UGT1A1* genotype-guided dosing has been adopted by several Dutch Hospitals.

Notwithstanding, *UGT1A1* genotype-guided dosing of irinotecan is not yet widely implemented in routine clinical practice. An important implementation barrier hindering implementation of *UGT1A1* genotype-guided dosing in routine clinical practice, clinical (oncology) guidelines and irinotecan drug labels is the concern for reduced treatment efficacy upon irinotecan dose reduction in UGT1A1 PMs [50,51]. Data from two studies revealed that systemic SN-38 exposure was similar in UGT1A1 PMs receiving 70% dose intensity compared with standard-dosed UGT1A1 normal metabolizers, demonstrating that that therapeutically effective systemic SN-38 exposure was maintained in UGT1A1 PMs [17, 20]. Likewise, two other clinical studies (n = 86 and n = 36) produced comparable tumor response rates in dose-reduced UGT1A1 PMs and standard-dosed intermediate and normal metabolizers [21,22]. So, the evidence thus far indicates that a priori dose reductions for UGT1A1 PMs do not impair irinotecan efficacy. However, new real-world-data studies are highly needed to confirm this in terms of long-term clinical outcomes like progression free survival and overall survival. To date, no studies have reported the impact on these outcomes.

Moreover, pretreatment genotyping for *UGT1A1* is a first successful step towards more personalized irinotecan treatment by identifying patients at high-risk for irinotecan toxicity. However, oncologist are still largely unable to predict irinotecan-associated toxicity for the majority of patients, as UGT1A1 PMs only account for around 10% patients [13]. In the study of Hulshof et al. (2022) we found that the incidence of severe irinotecan-induced toxicity still exceeded 30% in patients that were wildtype or heterozygous for *UGT1A1* polymorphisms [17]. Clearly, further identification and validation of prognostic and predictive factors for irinotecan treatment outcomes (toxicity and efficacy) are needed in order to further personalize irinotecan treatment.

Other genetic variations within the pharmacokinetic and the pharmacodynamic pathway of irinotecan may be associated with irinotecan-associated toxicity (or even efficacy) [23-31]. For instance, polymorphisms in genes coding for relevant membrane transporters (e.g. from the ATP-binding cassette (ABC) family or the solute carrier (SLC) family) may be associated with both irinotecan/SN-38 pharmacokinetics and irinotecan-related ADRs.

Factors other than the genotype may also contribute to irinotecan-induced severe toxicity, such as organ functions, age, sex, performance status and use of granulocyte colony-stimulating factor (G-CSF). Moreover, the interaction between patients' genetic background and sex-specific factors remains poorly understood. Females respond differently to anti-cancer chemotherapy, including irinotecan, compared to males and have higher risk (two-fold) to experience adverse drug reactions (ADRs) and hospitalization for an ADR [32,33]. Among other causes, there are differences in pharmacokinetics and pharmacodynamics, that modify drug bioavailability, and greater sensitivity to medication in females [34]. Furthermore, specific co-medications that are known for their gut microbiome modulating effects could play a role in irinotecan toxicity and efficacy. For instance, long term use of proton pomp inhibitors (PPI), antibiotics and oral corticosteroids may be associated with gut dysbiosis and increased infection risk [35]. This causes subsequent impairment of the effectiveness of chemotherapy, as was shown in non-small cell lung cancer and urothelial carcinoma patients with PPI use [36,37]. Conflicting results have been published for colorectal cancer patients, so it remains unclear whether the concurrent use of PPI negatively affects chemotherapeutic drugs such as irinotecan and 5-fluorouracil (5-FU) [38-40]. Additionally, β-glucuronidase activity derived from intestinal bacteria has been shown to hydrolyze the inactive metabolite SN-38G to the active SN-38. Therefore, gut microbiome modulating medications, such as antibiotics, could potentially affect irinotecan treatment outcomes. Lastly, cachexia-associated adverse body compositions have a strong negative impact on survival, therapy-associated toxicity, and cancer treatment outcome [41-45]. The individual body composition of patients with cancer may also affect the distribution of chemotherapeutic agents according to their hydrophilic or lipophilic properties. It has been shown that body composition parameters (skeletal muscle mass, visceral adipose tissue mass, subcutaneous adipose tissue mas, as well as the radiation attenuation of these tissue compartments) are associated with chemotherapy-induced toxicity (*paper under review*).

Thus, other non-genetic factors should therefore be comprehensively considered in predicting the risk of severe irinotecan-related toxicity and efficacy as well. Results regarding additional relevant factors/biomarkers associated with irinotecan-toxicity and/or efficacy require further exploration and validation in external cohorts.

Study rationale

This study expands on the results of Hulshof et al. (2022), which focused on the safety and feasibility of *UGT1A1* genotype-guided dosing of irinotecan. The impact of *UGT1A1* genotype-guided dosing on long term treatment outcomes in UGT1A1 PM is largely unknown. The main aim of the current study is to assess the impact of *UGT1A1* genotype-guided dosing of irinotecan on progression-free survival and overall survival. A traditional approach using a randomized clinical trial comparing survival in *UGT1A1* variant carriers treated with a full dose would be unethical and unfeasible because of the known increased risk of severe irinotecan-related toxicity. Therefore, we will compare survival outcomes of UGT1A1 PMs with an initial 30% dose reductions to UGT1A1 intermediate and normal metabolizers (IM and EM) with standard irinotecan dosages. A similar approach has recently been undertaken successfully for a survival study on *DPYD* genotype-guided dosing of fluoropyrimidines [46,47].

Secondly, the design and dataset of the current study will create a foundation and opportunity for other sub-studies/analyses to identify and validate prognostic and predictive factors for irinotecan treatment outcomes (toxicity and efficacy) other than *UGT1A1,* which could contribute to a more personalized irinotecan treatment. For instance, new prognostic/predictive factors may allow for improved pharmacogenetic dose individualization of irinotecan to improve safety and efficacy, and ultimately the quality of life of patients. Therefore, (new) relevant factors/biomarkers will be explored in two general sub-studies:

- Genome wide association sub-study**:** We hypothesize that genetic variations within the pharmacokinetic and the pharmacodynamic pathway of irinotecan, aside from the known relevant variations in *UGT1A1,* are associated with irinotecan-associated toxicity and altered response to treatment. Knowledge of these genetic variants will allow personalized therapy (recommendations) to help mitigate severe toxicity and even potentially increase efficacy of irinotecan-based treatment.
- Patient- and treatment characteristics association sub-study: Factors other than the genotype may contribute to irinotecan-induced severe toxicity, such as other chemotherapeutic drugs administered in the combination regimen, organ functions, age, sex, WHO performance status, use of G-CSF, PPI, antibiotics and corticosteroid use, body composition features and smoking status.

2 RESEARCH QUESTION / RESEARCH AIM

Primary objective
To determine the effect of an initial 70% irinotecan dose intensity in UGT1A1 poor metabolizers (PM) compared to a standard irinotecan dose (100% dose intensity) in UGT1A1 intermediate metabolizers (IM) and normal metabolizers (NM) on progression free survival (PFS), with a non-inferiority-based approach.

Secondary objectives

- To determine the effect of an initial 70% irinotecan dose intensity in UGT1A1 poor metabolizers (PM) compared to a standard irinotecan dose (100% dose intensity) in UGT1A1 intermediate metabolizers (IM) and normal metabolizers (NM) on overall survival (OS), with a non-inferiority-based approach.
- To determine overall treatment efficacy (ORR, PFS, OS).
- To compare systemic exposure of SN-38 and irinotecan in UGT1A1 PMs, IM and NMs that received *UGT1A1* genotype-guided dosing in relation to toxicity outcomes, PFS and OS.
- To determine and describe the incidence of irinotecan-induced grade 1-5 toxicity, toxicity-related hospital admissions, treatment delay and early treatment withdrawal.
- To get insights in whether safety of treatment would allow full *UGT1A1* genotype-guided dosing in patients with the normal UGT1A1 phenotype, that is, applying irinotecan dose escalations above 100% irinotecan dose intensity in case of good clinical and laboratory tolerance.
- To establish the population range of body composition features (skeletal muscle mass, visceral adipose tissue mass, subcutaneous adipose tissue mass, radiation attenuation of these tissue compartments) using routinely performed contrast-enhanced CT scans in the (portal-) venous phase.
- To investigate the correlation of patient- and treatment characteristics (i.e., sex, age, tumor type, stadium (local, locally advanced, metastatic), treatment schedule, number of cycles, dose modifications (escalation/reduction), WHO performance status, G-CSF use, PPI, corticosteroids, and antibiotics use, body composition parameters and smoking status) on treatment outcomes (PFS, OS and irinotecan-induced toxicity).
- To develop a risk nomogram to predict early-onset irinotecan-induced toxicity in UGT1A1 PM, IM and NM patients, based on relevant patient- and treatment characteristics.
- To identify novel germline DNA variant alleles associated with irinotecan toxicity and/or efficacy.

3 METHODS

3.1 Mono- or multicentre study

☐ Monocentre study

☒ Multicentre study

3.2 Study design

The present study is a retrospective multicenter cohort study. The cohort will consist of the IRI28-cohort [17] and an expansion cohort that will be newly included in the current study. Data is collected through electronic patient records of patients that are undergoing or have undergone standard-of-care treatment with systemic irinotecan in the period of August 2017-April 2024. Patients have been treated according to standard-of-care oncology guidelines in the Netherlands/local hospitals guidelines. Patients have been genotyped for *UGT1A1* pretreatment and dosed according to the DPWG guideline [14] (UGT1A1 PMs receive an initial 70% dose intensity of irinotecan, while IMs and NMs receive standard dosing of irinotecan (100% dose intensity)). As this is a retrospective study there will be no introduction of a new intervention.

3.3 Intervention

Not applicable

3.4 Study duration

For **retrospective** cohort study:

1. Inclusion period: patients that were treated with irinotecan in the period of Augustus 2017 until April 2024 in one of the participating clinical study sites.

2. Follow-up period: Included patients are followed-up via electronic patient records until death or until end-of-follow-up, whichever comes first. Follow-up ends in April 2025.

3. Start- and end date of the study (start data collection to completed primary analysis is anticipated to be May 2024 to May 2025.

3.5 Recruitment and selection of study population

3.5.1 Screening/selection

This study is a retrospective study in which data will be obtained from electronic patient records in HiX/EPIC.

- For the IRI28 cohort: Patients that were included in the IRI28-study will be screened to determine if they consented to their data being used for further research analyses. If not, these patients will be excluded from the list for the current study.

- For the expansion cohort: At each participating site, an extraction/list from HiX/EPIC of patients that received systemic irinotecan treatment between December 2020 up to April 2024 will be obtained through the Health Care Intelligence department. Patients will be screened for inclusion criteria (confirmation of treatment with systemic irinotecan) and patients that meet exclusion criteria will be excluded.

3.5.2 Study population

The study population consists of two groups:

1. IRI28 cohort: patients already included in the IRI28 study who will now be followed up for (long-term) treatment efficacy.
2. Expansion cohort: new to be included patients from Catharina Hospital Eindhoven (CZE), Erasmus Medical centre (Erasmus MC), Leiden University Medical Centre (LUMC), Jeroen Bosch Hospital (JBZ), Maasstad Hospital and RdGG Hospital Delft, who received treatment with irinotecan from December 2020 up to April 2024.

3.5.3 Inclusioncriteria

For the IRI28 cohort the inclusion criteria were as followed:

- Age ≥ 18
- A pathologically confirmed malignancy intended to be treated with irinotecan at a dose of ≥ 180 mg/m2 or 450-600 mg flat dose
- Written informed consent
- A WHO performance status of 0, 1 or 2
- Acceptable safety laboratory values:
  - Absolute neutrophil count (ANC) ≥ 1.5 x 109/L
  - Platelet count ≥ 100 x 109/L
  - Serum bilirubin ≤ 1.5 x upper limit of normal (ULN)
  - ALT and AST ≤ 2.5 x ULN; in case of liver metastases ALT and AST ≤ 5 x ULN
  - Renal function (eGFR) ≥ 50 ml/min or creatinine ≤ 1.5 x ULN

In order to be eligible for the expansion cohort, a subject must meet all of the following criteria:

- Having received treatment with systemic irinotecan (excluding liposomal irinotecan) regardless of dosage, WHO performance status or laboratory values at baseline

3.5.4 Exclusioncriteria

For the IRI28 cohort the exclusion criteria were as followed:

- Prior treatment with irinotecan
- Known substance abuse
- Psychotic disorders or other diseases expected to interfere with the study or the patient's safety
- Asian origin
- The use of (over the counter) medication or (herbal) supplements that were known to interact with irinotecan (e.g. by induction of inhibition of CYP3A4)

Additionally, patients from the IRI28 cohort that did not consent to their data being used for future research will be excluded.

A potential subject who meets any of the following criteria will be excluded from participation in the expansion cohort:

- Missing *UGT1A1* genotyping data
- Opt-out registration present for participation in scientific research

3.5.5 Sample size calculation

Our hypothesis is that *UGT1A1* genotype-guided dosing of irinotecan in UGT1A1 PM patients does not have a negative impact on PFS, the primary outcome in this study. We aim to demonstrate non-inferiority in PFS between UGT1A1 PMs that received an initial 70% irinotecan dose intensity and UGT1A1 IMs/NMs that received an initial 100% irinotecan dose intensity. Therefore, the sample size for this study was calculated using the software 'PASS' with a non-inferiority approach.

We calculated a sample size range using different scenarios based on the anticipated proportion of subjects that have reached progression at time of data collection, and an enriched vs. non-enriched analysis with UGT1A1 PMs. Around 10% of patients is expected to be UGT1A1 poor metabolizer [13]. Based on different scenarios, we chose a hazard ratio (HR) of 1,4 as accepted non-inferiority margin, to assess non-inferiority with a power of 80% and an alpha of 5%.

PASS output:

Non-Inferiority Tests for Two Survival Curves Using Cox's Proportional Hazards Model

Numeric Results with Ha: HR < HRni

Power	Alfa	Total Sample Size N	PM Sample Size N1	IM/NM Sample Size N2	Prop'n PM N1/N P1	Hazard Ratio h2/h1 HR	Non Inf Hazard Ratio HRni	PM Prob Event Pev1	IM/NM Prob Event Pev2	PM Events E1	IM/NM Events E2
0.8030	0.050	1020	102	918	0.100	1.000	1.400	0.600	0.600	61.2	550.8
0.8013	0.050	870	87	783	0.100	1.000	1.400	0.700	0.700	60.9	548.1
0.8007	0.050	760	76	684	0.100	1.000	1.400	0.800	0.800	60.8	547.2
0.8002	0.050	434	130	304	0.300	1.000	1.400	0.600	0.600	78.0	182.4
0.8020	0.050	374	112	262	0.300	1.000	1.400	0.700	0.700	78.4	183.4
0.8019	0.050	327	98	229	0.300	1.000	1.400	0.800	0.800	78.4	183.2

References

Chow, S.C., Shao, J., Wang, H. 2008. Sample Size Calculations in Clinical Research, 2nd Edition. Chapman & Hall/CRC.

Schoenfeld, David A. 1983. 'Sample Size Formula for the Proportional-Hazards Regression Model', Biometrics, Volume 39, Pages 499-503.

Report Definitions

Power is the probability of rejecting a false null hypothesis. Power should be close to one.
N is the total sample size.
N1 and N2 are the sample sizes of the PM and IM/NM groups.
P1 is the proportion of the total sample that is in the PM group, group 1.
HR is the hazard ratio: h2/h1.
HRni is the non-inferiority hazard ratio.
Pev1 and Pev2 are the probabilities of an event in the PM and the IM/NM groups.
E1 and E2 are the number of events required in the PM and the IM/NM groups.
Alpha is the probability of a type one error: rejecting a true null hypothesis.

Based on the PASS output above, in 'worst case scenario', a non-inferiority test of whether the hazard ratio with an overall sample size of 1020 subjects (of which 102 are in the UGT1A1 PM group and 918 are in the UGT1A1 IM/NM group) achieves 80% power at a 0,050 significance level when the hazard ratio is actually 1,000. The non-inferiority ratio is 1,400. The number of events (progression) required to achieve this power is 612. It is anticipated that the proportion of subjects observed with the event during the study is 0,600 for the PM group and 0,600 for the IM/NM group. These results assume that the hazard ratio is constant throughout the study and that Cox proportional hazards regression or the non-inferiority log-rank test is used to analyze the data.

Advanced/metastatic colorectal cancer (CRC) and advanced/metastatic pancreatic cancer (PC) are anticipated to be the largest cancer type groups in the current study (based on numbers in Hulshof et al. 2022). For metastatic CRC patients treated with an irinotecan-based regimen the median PFS is around 8-12 months and for metastatic PC treated with an irinotecan-based regimen the median PFS is around 6 months. Therefore, based on a study population included between August 2017 to April 2024, it is expected that the majority of subjects will have met the primary endpoint (progression) and secondary endpoint (death).

A hazard ratio accepted as 'non inferior' margin was defined as an hazard ratio of 1.4, based on the golden standard PASKWIL-criteria (hazard ratio cut-off set at 0.6). However, a hazard ratio lower than 1.4 is desired if possible, for which a larger sample size would be needed. Moreover, the analysis may be stratified for CRC and PC patients. Lastly, loss-to-follow-up an missing data needs to be taken into account. Therefore, we will maximize our sample size to the total number of eligible patients in our participating centres. Enriching the study population with UGT1A1 PMs will also increase power.

The total number of potentially eligible patients was assessed at our participating study sites based on the number of UGT1A1 poor metabolizers from January 2021-March 2024:

- CZE: 29
- EMC: 28
- LUMC: 14
- JBZ: 16
- Maasstad: 14
- RdGG Delft: 14

Total: 115 UGT1A1 PMs + additional 31 UGT1A1 PMs from IRI28 cohort = 146 UGT1A1 poor metabolizers. Assuming that accounts for 10% of all irinotecan-treated patients, there will be 1460 UGT1A1 intermediate/normal metabolizer patients available for enrolment.

Thus, maximizing the sample size to all eligible *UGT1A1* genotyped patients with irinotecan treatment in the period of August 2017-April 2024 in these six centres is feasible and will enable a sample size required for a well-powered non-inferiority survival analysis. The incidence of loss to follow-up is expected to be low as treatment response is closely monitored and registered in the EHC and death is registered in a national register. A total sample size around 1000-1500 is anticipated.

3.6 Data collection: variables and methods

3.6.1 Primary endpoint

The primary endpoint for this study is the progression free survival of PM treated with 70% dose irinotecan compared to IM and NM treated with standard dosing of irinotecan (100%). Patient are treated with irinotecan conform the standard care in the included hospitals. *UGT1A1* genotyping is performed pre-treatment for all patients.

Progression is defined as: Death from any cause, radiological progression confirmed by RECIST 1.1 criteria, clinical progression that is treated as such by the treating physician (radiotherapy for oligometastases or switch to other line of treatment), whichever came first.

3.6.2 Overview of variables and methods

Variabele	Source	Outcome value	Moment of measurement /collection
**Progression free survival (primary endpoint)**	EPD<br><br>Radiology reports cf RECIST 1.1 for progression (radiology reports)	Categorical: Single answer (progression/ no progression)<br><br>Categorical (date progression)<br><br>Categorical: Progression conform: single answer (RECIST 1.1 criteria/ death/ other)	Conform standard care

**Overall survival**	EPD/BSN registry	Categorical: Single answer (living/ deceased)<br><br>Categorical (date death)<br><br>Categorical (date last known to be alive)	Conform standard care
**Tumor response**	EPD<br><br>RECIST 1.1 (radiology reports)	Categorical: Single answer (complete response/ partial response / stable disease / progressive disease)	Conform standard care<br><br>Collec
**Sex**	EPD	Categorical: Single answer (man/woman)	Baseline
**Age**	EPD	Numeric	Baseline
**Ethnic origin**	EPD	Categorical: Single answer (African/European/Asian/Hispanic/ other(specify)/ unknown)	Baseline
**Body surface area (Dubois) (m^2)**	EPD	Numeric	Baseline and at each cycle
**Primary tumor type**	EPD	Categorical: Single answer (Colorectal / Pancreas/gastric/esophagus/biliary tract/Other)<br>* For other specify which type	Baseline
**Disease stadium**	EPD	Categorical: Single answer (Local/ locally advanced/ metastatic)	Baseline
**WHO performance status**	EPD	Categorical: Single answer (0/1/2/3/4/unknown)	Baseline
**Smoking status**	EPD	Catergorical: Single answer (Smoker/nonsmoker/unknown)	Baseline
**ASAT**	EPD	Numeric	Baseline
**ALAT**	EPD	Numeric	Baseline
**Alkalische fosfatase**	EPD	Numeric	Baseline
**LDH**	EPD	Numeric	Baseline
**Total bilirubin**	EPD	Numeric	Baseline
**Conjugated bilirubin**	EPD	Numeric	Baseline
**eGFR**	EPD	Numeric	Baseline
**Co-medication: PPI use et baseline (≥14 days)**	EPD	Categorical: Single answer: Yes/No<br><br>If yes: specify PPI (free text), dosage and duration	Baseline
**Co-medication: Antibiotics use (within 30 days before start irinotecan treatment schedule)**	EPD	Categorical: Single answer: Yes/No<br><br>If yes: specify antibiotic (free text), dosage and duration	Baseline
**Co-medication: Corticosteroid use (≥10 mg prednisone equivalent for**		Categorical: Single answer: Yes/No	Baseline

**≥24 hours within 30 days before start irinotecan treatment schedule**		If yes: specify corticosteroid (free text), dosage and duration	
UGT1A1*28	EPD	Categorical: Single answer (wildtype/ heterozygous/ homozygous/ unknown)	Baseline
UGT1A1*6	EPD	Categorical: Single answer (wildtype/ heterozygous/ homozygous/ unknown)	Baseline
UGT1A1*36	EPD	Categorical: Single answer (wildtype/ heterozygous/ homozygous/ unknown)	Baseline
UGT1A1*37	EPD	Categorical: Single answer (wildtype/ heterozygous/ homozygous/ unknown)	Baseline
UGT1A1*93	EPD	Categorical: Single answer (wildtype/ heterozygous/ homozygous/ unknown)	Baseline
**UGT1A1 (converted) phenotype**	EPD	Categorical: Single answer (PM/IM/NM/unknown)	Baseline
***DPYD* genotype (if co-administration of 5-FU)**	EPD	If *DPYD* gene activity score < 2: Define genotype: (heterozygous or homozygous for relevant mutations (c.1236G>A, *2A, *13, c.2846A>T, *7))	Baseline
***DPYD* gene activity score (if co-administration of 5-FU)**	EPD	Categorical: Single answer (0/0.5/1/1.5/2.0)	Baseline
**Previous cancer treatment -How many lines of treatment?**	EPD	Categorical: Single answer (0/1/ 2/ 3/ 4/ 5/ unknown)	Baseline
**Previous cancer treatment – surgery?**	EPD	Categorical: Single answer (yes/no)<br><br>If yes: define surgery	Baseline
**Previous cancer treatment – radiotherapy?**	EPD	Categorical: Single answer (yes/no)	Baseline
**Treatment indication**	EPD	Categorical: single answer (neoadjuvant/adjuvant/palliative)	Baseline
**Treatment schedule**	EPD	Categorical: Single answer<br>- irinotecan monotherapy q2w<br>- irinotecan monotherapy q3w<br>- irinotecan + panitumumab q2w	Baseline

		- FOLFIRI q2w<br>- FOLFIRI + targeted therapy q2w<br>- FOLFIRINOX q2w<br>- mFOLFIRINOX q2w<br>- FOLFOXIRI q2w<br>- Other, specify: (free text)	
**Standard daily irinotecan dose for WT patients with same indication/schedule (mg)**	EPD	Numeric	Baseline
**Standard daily irinotecan dose for WT patients with same indication/schedule (mg/m²)**	EPD	Numeric	Baseline
**Irinotecan dosage received (mg)**	EPD	Numeric	At each cycle
**Irinotecan dosage received (mg/m²)**	EPD	Numeric	At each cycle
**Dose intensity irinotecan received (%)**	EPD	Numeric	At each cycle
**Start date treatment cycle**	EPD	Categorical: date	At each cycle
**Dose modification irinotecan (≥ 10%)**	EPD	Categorical: Single answer (yes/no)<br>If yes: Categorical: Single answer (reduction/ escalation)<br>If yes: Reason dose modification: Single answer	At each cycle
**Dose modification concomitant anticancer drugs (≥ 10%)**	EPD	Categorical: Single answer (yes/no)<br>If yes: Categorical: Single answer (reduction/ escalation)	At each cycle
**Febrile neutropenia**	EPD<br>CTCAE 5.0	Categorical: Single answer (no febrile neutropenia, grade 1, grade 2, grade 3, grade 4, grade 5)	Cycles 1-3
**Neutropenia**	EPD<br>CTCAE 5.0	Categorical: Single answer (no neutropenia, grade 1, grade 2, grade 3, grade 4, grade 5)	Cycles 1-3
**Diarrhea**	EPD<br>CTCAE 5.0	Categorical: Single answer (no diarrhea, grade 1, grade 2, grade 3, grade 4, grade 5)	Cycles 1-3
**Toxicity-related hospitalization**	EPD	Categorical: Single answer (yes/no)	Cycles 1-3

		If yes: specify toxicity: singly answer	
**Adverse drug reaction relation to irinotecan**	EPD	Categorical: Single answer (no relation/unlikely/possibly/ probably/definitely)	Cycles 1-3
**Treatment delay (defined as delay of > 2 days)**	EPD	Categorical: Single answer (yes/no)<br><br>If yes; reason delay: single answer (logistic/toxicity/other) and duration of delay	Cycles 1-3
**Co-medication: G-CSF use**	EPD	Categorical: Single answer: Yes/No	Cycles 1-3
**Co-medication: atropine use**	EPD	Categorical: Single answer: Yes/No	Cycles 1-3
**Systemic exposure SN-38 and irinotecan**	EPD (if available)	Numeric	Cycle 1
**Last irinotecan cycle number**	EPD	Numeric	End of follow-up
**Number of treatment (irinotecan) cycles received**	EPD	Numeric	End of follow-up
**Irinotecan treatment ongoing?**	EPD	Categorical: Single answer (yes/no)	End of follow-up
**Reason end of irinotecan treatment**	EPD	Categorical: Single answer (toxicity/ end of treatment per protocol/ disease progression/ patient refusal/ death/ other)	End of follow-up
**Treatment lines after irinotecan treatment schedule**	EPD	Categorical: Single answer (1/ 2/ 3/ unknown)	End of follow-up
**Body composition***	Analysis of L3 CT scan (available in EPD)	Numeric for parameters:<br><br>Skeletal muscle (SM) index in cm/m^2, subcutaneous adipose tissue (SAT) index in cm/m^2, visceral adipose tissue (VAT) index in cm/m^2, radiation attenuation (RA) of SM/SAT/VAT in Hounsfield Units/sarcopenia	Retrospectively/ end of study<br><br>Conform standard-of-care performed CT scans (baseline and follow-up during treatment)
**GWAS****	Leftover blood plasma/ germline DNA sample	NA: GWAS pipeline	Retrospectively/ end of study

***** For studying body composition, available routine diagnostic abdominal L3 CT-scans will be analyzed with Mosamatic (by Maastricht University). In this way, body composition features may be identified as covariates for irinotecan toxicity.

****** An EDTA blood plasma/DNA sample that has been obtained for the routinely performed *UGT1A1* genotyping test is in retrospect used for additional pharmacogenetic analyses. A GWAS pipeline set up at the LUMC KFT department will be used for these analyses.

3.6.3 Standardization

RECIST 1.1

For the radiological assessment of disease progression, the Response evaluation criteria in solid tumors (RECIST) v1.1 is used as standard of care in the clinical setting. This is a set of criteria used to assess a tumor's response to treatment using radiological (CT) scans. Response is divided into the following categories: complete response (CR), partial response (PR), stable disease (SD) and progressive disease (PD). The RECIST 1.1 criteria are used to assess the primary outcome progression free survival (PFS), as this is used as standard of care in radiology. 'Response/progression' recorded by the radiologist in standard radiology reports of CT scans will be used to assess the disease progression.

CTCAE v5.0

To assess the severity of side effects in oncology, the Common Terminology Criteria for Adverse Events (CTCAE) are used. This is the gold standard to assess and classify the severity of side effects in a standardized way. Side effects are given a grade, 1 to 5, respectively mild, moderate, severe, life-threatening and death. The CTCAE version 5.0 will be used to assess the severity of (febrile) neutropenia and diarrhea, outcome measures for toxicity.

3.7 Data-analysis

3.7.1 Data-inspection

Quality checks (validations) will be implemented in Research Manager EDC (eCRF) to improve data entry quality. A standard-operating-procedure/instruction will be made for data entry into the eCRF. Researchers will check/inspect a part of the imputed data into the eCRF at different sites to check for data entry errors. Separate eCRFs are used for the IRI28-cohort and the expansion cohort.

Missing data will be tracked in Research Manager EDC. In case of missing data, these are marked as missing completely at random (MCAR) or no missing at random (NMAR). A complete case analysis will be performed for the primary aim. A complete case is based on data variables that are considered relevant for assessment of the primary aim (survival analysis), that is; sex, age, primary tumor type, stage of cancer, treatment regimen, irinotecan dosage, UGT1A1 phenotype/genotype, number of previous treatment lines, last irinotecan treatment cycle, PFS, OS. Patients with missing data regarding disease progression are censored in the primary analysis.

Data will be checked for outliers in the case of normally distributed data. Outliers will be identified visually through histograms/boxplots, residuals and/or the concordance c-index, depending on data type. Where applicable, the median and quartile range will be used for data description as they are less sensitive to outliers. Outliers may be excluded in the analysis.

Data cleaning may be performed to track the original data and modify abnormal values in case of data entry errors. Extractions from the IRI28-cohort eCRF en the expansion cohort eCRF will be combined into one extraction file in Excel and/or into one eCRF. Data cleaning steps will be performed by the researcher. All data cleaning steps will be logged and documented in a separate .docx file.

A data dictionary (code book) will be available for the clinical data, exported from the structure in Research Manager. All syntaxes used in data cleaning and analysis (including annotation describing the goal of processing steps) will be stored to facilitate replication.

3.7.2 Analyses

A detailed statistical analysis plan is attached to this protocol as Supplementary File. In brief:

Primary survival analysis

The primary survival analysis investigates the effect of the reduced irinotecan dose on treatment efficacy in UGT1A1 poor metabolizers from the IRI28 study, enriched with all UGT1A1 poor metabolizers who were treated according to a similar protocol with the same dose reductions as part of routine clinical care between December 2020 and April 2024 in CZE, EMC and LUMC.

Exposure group and control group

The exposure group is defined as UGT1A1 poor metabolizers treated with UGT1A1 genotype-guided dosing of irinotecan, that is, an initial 70% dose intensity of irinotecan. The control group is defined as UGT1A1 intermediate or normal metabolizers treated with an initial 100% dose intensity of irinotecan. In practice it can occur that a PM receives a higher dose intensity than 70% and that a IM/NM receives lower dose intensity than 100%. These cases will be censored and will be excluded in the primary analyses (per-protocol analysis). These patients will however be included in a secondary analysis, in which all patients treated with a UGT1A1 genotype-guided dosing of irinotecan will be included (intention-to-treat analysis).

Timing of outcome assessments

Patient outcomes registered in the EPD will be used for the current study. If survival status is unknown in the EPD of the clinical study site, this status will be retrieved through the treating physician's dossier or the *'BSN registry*. The follow-up schedule is summarized under 3.6.2.

Level of significance and multiple testing

All applicable statistical tests will be 2-sided and will be performed using a 5% significance level. All confidence intervals presented will be 95% and 2-sided. If applicable, correction for multiple testing, such as the Bonferroni correction, will be applied

Recruitment

A patient flow diagram will be reported and will display all stages of the study, including screening, exclusions, enrollment, follow-up.

Baseline characteristics

Demographic information (e.g., age, sex, ethnicity, among others), clinical characteristics at baseline (e.g., tumor type, tumor stage, among others) will be presented for each group. Mean (±standard deviation) or median and interquartile range (IQR) will be used for describing continuous variables, and frequency (percentage) will be used for describing categorical variables. Standardized differences will be used to examine the balance in baseline covariates between UGT1A1 poor metabolizers and intermediate/normal metabolizers.

Primary outcome PFS and secondary outcome OS

PFS is defined as the time between initiation of treatment and first signs of disease progression by either clinical signs (including switch to other treatment line and radiotherapy for oligometastases), or radiological imaging or death from any cause, whichever comes first.
OS is defined as the time between initiation of treatment and death from any cause. Patients not experiencing disease progression or death before the end of follow-up are and loss-to-follow-up patients are censored at the last date known to be alive. PFS and OS will be compared between UGT1A1 PMs (exposure group) and UGT1A1 IMs/NMs (control group). As a secondary analysis, PFS and OS will be compared between UGT1A1 IM and NM patients.
PFS and OS curves are generated using the Kaplan-Meier method. A (stratified) log-rank test is used to compare survival between UGT1A1 poor metabolizers and intermediate/normal metabolizers. Univariable Cox regression analysis will be performed to test the association between *UGT1A1* status and PFS and OS. Hazard ratios (HRs) and their corresponding 95% CI will be obtained. Median follow-up is calculated using the reverse Kaplan-Meier method.

In addition, univariable and multivariable Cox regression analyses are performed for all available UGT1A1 poor metabolizers and intermediate/normal metabolizers, adjusted for relevant covariates. Covariates that are anticipated to be relevant for outcomes PFS and OS are: sex, age, primary tumor type, stage of cancer, treatment regimen, irinotecan dosage, irinotecan dose escalations/reductions, *UGT1A1* phenotype/genotype, number of previous treatment lines, number of irinotecan treatment cycles.
The primary analysis/cox regression analyses are performed stratified for individual tumor types and stages (colorectal and pancreatic cancer, advanced and metastatic). Other tumor types and tumors in the local setting are described separately. The stratified Cox regression is an approach that assumes a common HR, but different baseline hazards. Schoenfeld residuals are used to verify the proportional hazards assumption. The primary analysis is also performed for all advanced and metastatic CRC and PC patients pooled only if relevant covariates are distributed equally between exposure and control group.

Sensitivity analyses

Sensitivity analyses are performed to explore the robustness of the results from primary analysis.
If more than 10% of the patients is lost to follow-up a sensitivity analysis will be performed on the baseline characteristics to check whether there is selective loss to follow-up.

Interim analysis
In June 2024 an interim analysis will be done for the IRI28 cohort (primary survival analysis).

Timing of final analysis
Data collection for the primary analysis/primary endpoint is anticipated to be completed by Q1 of 2025. The primary data analysis for the primary endpoints is anticipated to take place in April/May 2025

Secondary analyses
For the secondary aims/outcomes, separate SAPs will be composed before start of analyses. In brief, the following analyses will be performed:

Toxicity
Toxicity endpoints (severe (grade ≥ 3) neutropenia and/or diarrhea, toxicity-associated hospitalization, treatment delay and early treatment withdrawal, among others), which will be presented as n (%). Primarily, these outcomes will be compared between patients from the expansion cohort that received *UGT1A1* genotype-guided dosing and historical controls without *UGT1A1* genotype-guided dosing by using Chi-square test or Fisher's exact test, where appropriate. Historical controls are selected from published studies identified from a systematic literature as described in the IRI28 study (Hulshof et al. 2022). In addition to comparison with historical controls, toxicity of UGT1A1 PMs will also be compared to standard-dosed UGT1A1 IM/NMs from the patients in the expansion cohort of this study, under the hypothesis that dose adaptation in UGT1A1 PMs results in comparable toxicity incidences as UGT1A1 IM/NM patients treated at the standard dose. A subgroup analysis will be undertaken to assess if toxicity endpoints are influenced by differences in treatment schedules; monotherapy irinotecan versus combination therapy including irinotecan. It is tested whether the starting dose intensity of 70% is a safe dose, or whether it can be increased or needs to be decreased. This will be calculated by the average of the given doses that did not result in severe toxicity and/or treatment delay. Results will also be analyzed separately for the expansion cohort, as a real-world setting that can be compared to the results from the original IRI28 trial.
Results from these analyses will further strengthen results from Hulshof et al. 2022 in a larger cohort.
Secondly, toxicity endpoints will be used for other sub-analyses (see below).
Pharmacokinetics
If available, pharmacokinetic parameter AUC of irinotecan and SN-38 will be collected and will be presented as median ± IQR. The PK data will be compared between groups and tested for association with treatment efficacy (PFS and OS) and toxicity.

Genome wide association study in relation to irinotecan toxicity and/or efficacy
We will use leftover blood samples from included patients to perform DNA isolation and DNA genotyping with a Global Screening Array. Genetic markers will be excluded based on a minor allele frequency threshold. For each genetic marker, the association with toxicity outcomes, PFS and OS will be assessed. The additional retrospective genotyping analysis at study end will be analyzed in a separate report and is explorative in nature. It will test multiple comparisons/genotypes with treatment outcome; therefore, the Bonferroni correction will be applied for correction for multiple testing.

Body composition parameters
L3 CT scans will be analyzed for parameters skeletal muscle mass, visceral adipose tissue mass, subcutaneous adipose tissue mas, as well as the radiation attenuation of these tissue compartments. Mosamatic (www.mosamatic.com), an artificial intelligence-based approach for automated analysis of CT-scans [https://www.medrxiv.org/content/10.1101/2023.04.23.23288981v1], will be used.

Covariates (patient- and treatment characteristics) association with irinotecan toxicity and/or efficacy
Characteristics for continuous variables will be summarized as mean (±standard deviation) or median (interquartile range), depending on their distribution. For categorical variables, frequency and percentage are presented.

Categorical variables will be compared using Pearson’s chi-square test (Fisher’s exact test in case of sparse data) and the Mann– Whitney U test will be used to test differences in continuous variables. Univariable and multivariable logistic regression models may be used in the development of the irinotecan-induced toxicity prediction model for a nomogram. Correlations between variables will be assessed using Pearson’s and Spearman’s correlation coefficients. Results of regression analyses are reported as odds ratios with 95% confidence intervals.

Variables in general

All binary and categorical variables are expressed as n (%), and continuous variables as mean (standard deviation), or median [range], depending on distribution. Patient demographics will be analyzed using descriptive statistics. Data will be tested for normal distribution. Associations with dichotomous outcomes will be analyzed using chi square tests or Fisher’s exact test, where appropriate. Continuous variables, such as pharmacokinetic parameters will be analyzed using student’s t-test. Because we assume that the serum concentrations of irinotecan have a log-normal distribution the analysis will be performed on the log-transformed data. All statistical tests are performed 2-sided. All binary and categorical variables are expressed as n (%), and continuous variables as mean (standard deviation), or median [range], depending on distribution. All co-variables will be tested using univariate and multivariate regression. Results of regression analyses are reported as odds ratios with 95% confidence intervals. All applicable statistical tests will be 2-sided and will be performed using a 5% significance level. If applicable, correction for multiple testing, such as the Bonferroni correction, will be applied.

3.7.3 Softwares

For data collection, the EDC module of Research Manager will be used. For data analysis, SPSS and/or R will be used. Graphpad Prism will be used for the visualization of data in plots/graphs.

4 ETHICAL CONSIDERATIONS

4.1 Niet WMO verklaring

This study is a follow-up and extension are of the IRI28 study [17]. Results will be obtained from electronic patient records. Residual blood/DNA samples will be used for the additional GWAS analysis. Patient will not be subjected to interventions or (burdensome) questionnaires for this study. All treatment and examinations have already been performed in accordance with standard treatment guidelines. For this reason, this study does not fall under the Medical Research Involving Human Subjects Act (WMO). The study will be submitted to the Medical Ethics Committees United (MEC-U) for application of an non-WMO statement and exception for informed consent procedure.

4.2 Compensation for study subjects

Not applicable

4.3 Informed consent study subjects

In prospective non-WMO research, consent must always be sought from the subject. In the case of retrospective non-WMO research, this depends on the method of data collection and its processing. If in doubt, you can use the decision tree in Appendix A.

**Is informed consent asked?**	☐ Yes (Fill in option A below)<br>☒ No (Fill in option B below)
**Option A: Informed Consent procedure**	
**Recruiting subjects**	Not applicable
**Informing subjects**	Not applicable
**Reflection time**	Not applicable
**Signing informed consent**	Not applicable
**Optie B: Informed consent is not asked**	

Which exception rule(s) applies? Explain your answer at the bottom of this question.
Exception rules:

☐ Requesting consent is not reasonably possible because asking consent would place such a burden on the patient that psychological harm must be feared.

☒ Requesting consent is not reasonably possible because the individual is deceased or the address cannot be ascertained or if the individual does not respond after being written to on at least two occasions.

☐ Requesting consent is not reasonably possible because it involves estimating the right sample and asking for consent would require asking many more individuals than are necessary to answer the research questions.

☐ Requesting consent is not reasonably possible because the consent question cannot be meaningfully asked because the study is still in an initial preparation phase.

☒ Consent cannot be desired because asking for consent would require disproportionate time and effort, such as in the case of large numbers of patients or patients who were treated a long time ago.

☒ Consent cannot be desired because asking for consent leads to selective response and therefore a biased picture of the survey results can be expected.

☐ Consent cannot be desired because <click here to enter text>

☒ Requesting consent is not necessary because it involves research with data from one's own patients.

Explanation

The exception for the request for informed consent applies to the part of the study population that will be newly included (expansion cohort), and partly to the IRI28 cohort. For the follow-up of the IRI28 study cohort, informed consent has already been obtained for obtaining (medical) patient data for survival analysis and follow-up research. This includes collection of patient and treatment characteristics, progression and death data from HiX, and use of remaining DNA samples for additional pharmacogenetics analyses (GWAS). IRI28 study subjects who did not consent to this in informed consent are excluded from the current study.

The following exemption rules apply to all data collection of the expansion cohort. The following also applies to the collection and analysis of existing CT scans and the retrieval of death data from the BSN registry from of the IRI28 cohort (these two items were not specifically mentioned in the original IRI28 study protocol):

- **Requesting informed consent is not reasonably possible for this study.** The population size calculated to achieve a power of 80% is around 1000-1500 patients (see section 3.5.5). This would take very much time and effort to have to approach all these patients (multiple times) for consent, which would make the study infeasible. Also, all treatments relevant to this study have already been performed in the past. A greater obstacle to seeking consent is the fact that the majority of these patients will already be deceased, making it impossible to seek consent. The study population will consist mainly of patients with colorectal or pancreatic carcinoma in the advanced or metastatic setting during the period from August 2017 to April 2024. The 5-year survival rate for patients with colorectal carcinoma is 60% and for pancreatic carcinoma it is only 5% (pooled disease stages) [48,49]. For patients with advanced or metastatic disease treated with an irinotecan regimen, median overall survival is ~15-25 months in CRC patients and ~10 months in PC patients [2-6].
- **Requesting consent from only living patients cannot be desired because this would lead to a selective response** of only still living patients, which may bias study results in the survival analysis (with PFS and OS as important outcome measures) of this study. Asking consent of family members from deceased patients is also considered undesirable in this situation. This would be (unnecessarily) too burdensome for family members, also considering that no new data are generated.

Because of the abovementioned reasons, the present study qualifies for the exception rules. The results of this study will be of great value to all patients treated with irinotecan in daily practice (see Section 6.1). Without the data from this study population, the study cannot be realized.

All data will be used coded so that it cannot be traced back to individual patients. The data will only be used for the research purposes described in this study protocol. If the EPD of an expansion cohort patient states that this patient has objected to the use of their (medical) data for scientific research, this patient will be excluded from the current study. This will be verified by checking opt-out in the EPD of each patient.

5 DATAMANAGEMENT & PRIVACY

5.1 5.1 Data storage, security and access during research

Paper research data

5.1.1 Where are paper research data (e.g., paper questionnaires and informed consent forms) stored?

☒ Not applicable. *Go to question 5.1.4*

5.1.2 Who have access to this storage? This should be at least two people i.e. access to the data in case of absence, illness, leaving the institution, etc. when only one person has access.

5.1.3 How is it ensured that no one other than the authorized people listed in 5.1.2 has access to the paper research data?

Digital research data

5.1.4 In what system will the research data be collected and managed?

☒ Castor EDC / **Research Manager** / *Data will be collected and stored as an electronic Case Report Form (eCRF) in eClinical datamanagement platform ResearchManager. Access to ResearchManager can be granted by the following people: PI of the study, Head of department, Data Accessor , Departmental Science Committee.*

☐ Other GCP-proof data collection system, namely <Click here if you want to enter text>.

☐ In an (Excel or SPSS) file, stored in the study folder on SharePoint Science, password protected and accessible only to the study team.

☒ Other: After the eCRF is complete, an extraction of the data is made which is used for further analysis in SPSS and/or R. This extraction is protected with a password which is only available to the study team. Extractions from the IRI28 cohort eCRF and expansion cohort eCRF are merged into one eCRF/extraction output. *Access to file can be granted by the following people: PI of the study, Head of department, Data Access or Departmental Science Committee.*

5.1.5 Is digital research data (also) stored on a hospital network drive

☒ Yes, exported data file will be stored on the network drive of participating hospitals and always encrypted/coded data.

Go to question *5.1.6*

☐ No, the digital research data are stored at <location>. Go to question 5.2.1

5.1.6 Who have access to this location on the network drive? This should be at least two people i.e. access to the data in case of absence, illness, leaving the institution, etc. when only one person has access.

People with access to the location/data on the network drive are the principal investigator and co-investigators of the participating centers and the department head.

5.1.7 How is it ensured that no one other than the study team has access to this location on the network drive (multiple answers possible)?

☒ Only the study team has access authority.

☐ Study folder is secured with a password.

☒ Study documents secured with a password: password is only available to the research team, department head and data accessor.

5.2 Data processing

5.2.1 Will research data be anonymized or coded?

☐ Anonymized *Go to question 5.2.4*

☒ Coded *Go to question 5.2.2*

5.2.2 How will data be coded?

All data in this study will be coded, so that patient data cannot be directly traced back to individual patients. Data that can be traced back to patients, such as initials or dates of birth, will not be used. Coding will be done as followed: CZE001, CZE002, CZE003, EMC001, EMC002,… The first three letters stands for the participating center and last three numbers are for each patient individually. Only this study-ID will be used in study documentation, rapports and publications. The key to the code is safeguarded by a local site investigator from the study team at each participating study site, or the person this task has been delegated to. People/authorities that are allowed access to the code key are the members of the accredited METC, the Health Inspection, and members from the research team with approval of the principal investigator or head of department. Permission to see the code key for the members of the accredited METC and the Health Inspection is necessary in case of an inspection of the accuracy and quality of the study.

5.2.3 Where is the subject identification code list (key between coded and patient traceable data) stored?

☒ In the study folder on the departmental disk, password-protected and accessible only to the study team. The key does not leave the relevant participating local site. (Only allowed if saving to SharePoint Science is not possible): *Each data code list (one list per participating clinical study site) will be stored within each participating clinical study site where the participants have been included. Data code-lists files are stored electronically on a departmental server safeguarded by a password that is only accessible to the study team of the local site and data accessor. Access to data code lists is only granted by the principal investigator, head of department or data accessor of the corresponding clinical study site to individual members of the study team.*

☐ External. For external storage of the key including access authorisations, permission is requested from the patient in the patient information form (PIF).

5.2.4 By whom is the required data extracted from the EHR and anonymised or coded?

☐ Poople who are under the authority of the responsible physician/principal investigator/head of department, namely: Data collectors of the study team working at the local study site may extract and code the required data from the EHR of the patients of that associated study site. The principal investigator must give permission for this.

5.3 Data delen

5.3.1 Will research data be shared with third parties?

☐ Yes, annonymized, *go to question 5.3.2*

☒ Yes, coded, *go to question 5.3.2*

☐ No, *go to question 5.4*

5.3.2 To whom is the data provided / who uses the data for scientific research?

Institutions/researchers within the Netherlands, namely: Maastricht University: Only L3 CT scans at baseline and follow-up during treatment with irinotecan will be provided for analysis of the CT scans (see 3.6.2.). Data parameters of analysed CT scans will be sent back coded to the relevant site from which the CT scans originated. A Data Transfer Agreement will be drawn up for this purpose.

The extraction of the complete eCRF can be used for analysis by members of the study team at any of the named participating local sites. This extraction will be password protected. *Access to file can be granted by the following people: PI of the study site, Head of department, Data Accessor or Departmental Science Committee. Hiervoor zal een Data Transfer Agreement worden opgesteld*

5.4 How long is data stored?

The data will be kept for 15 years at the Catharina Hospital Eindhoven, corresponding to applicable guidelines.

6 VALORISATION AND PUBLICATION

6.1 Valorisation

In the emerging era of precision oncology, pre-therapeutic genotyping for somatic mutations is routinely used to determine tumour response to anticancer drugs. To complement this, pre-therapeutic genotyping for germline genetic variants to guide anticancer drug dosing, known as pharmacogenetics (PGx), is increasingly recognised as essential for achieving safe and effective drug treatment. The current study is an important step towards precision dosing of classical anticancer drugs, specifically irinotecan.

Demonstration of non-inferiority of UGT1A1 genotype-controlled dosing of irinotecan in this study may lead to promotion of (inter)national implementation of UGT1A1 genotype-controlled dosing as standard care for all patients treated with irinotecan. The DPWG and some other European pharmacogenetics guidelines already recommend genotyping for UGT1A1 prior to treatment and treating UGT1A1 PM with an initial a dose intensity irinotecan of 70% to avoid severe toxicity. As yet, not all Dutch hospitals follow this advice. Outside the Netherlands, standard UGT1A1 genotyping is even more limited. There is still some reluctance among treatment providers due to lack of scientific evidence regarding the efficacy on hard endpoints (PFS and OS) of this reduced dosing in UGT1A1 PM patients. Prior to the current study, there has been no research on the survival of UGT1A1 PMs with 70% dosing compared to IM and NM with standard dosing. The results from this study are therefore of great significance. Favourable results could promote the inclusion of UGT1A1 genotype-driven dosing recommendations in oncology guidelines and the Irinotecan package insert. On the other hand, the current study could provide insights around further optimisation of UGT1A1 genotype-driven dosing of irinotecan.

Further, regarding the substudies/subanalyses of this study: To date, the identification and validation of biomarkers in addition to UGT1A1 that are predictive of treatment with irinotecan has remained largely unexplored. Moreover, other important patient and treatment characteristics may determine the individual profile of toxicity and efficacy, but these are still rarely routinely considered in irinotecan treatment. Identifying and validating new relevant pharmacogenetic biomarkers and patient characteristics (or combinations thereof) fills a clinical need in oncology, as these factors could enable further personalisation and improvement of current treatment with irinotecan through, for example, precision dosing.

6.2 Publication

The results from this study will be submitted as scientific articles to peer-reviewed journals for publication and as abstracts at scientific congresses/symposia in the field of oncology/pharmacogenetics/pharmacy/chemotherapy, in order to contribute to new scientific insights. Which specific journals and congresses these will be will be determined at a later date.

7 REFERENCES

1. Bailly C. Irinotecan: 25 years of cancer treatment. Pharmacol Res. 2019;148:104398.
2. UpToDate. Initial systemic therapy for metastatic exocrine pancreatic cancer. [Internet]. Available from: https://www.uptodate.com/contents/initial-systemic-therapy-for-metastatic-exocrine-pancreatic-cancer?search=metastatic%20pancreatic%20cancer&source=search_result&selectedTitle=1%7E69&usage_type=default&display_rank=1 [Accessed 27th March 2024].

3. UpToDate. Initial systemic therapy for metastatic colorectal cancer. [Internet]. Available from: https://www.uptodate.com/contents/systemic-therapy-for-metastatic-colorectal-cancer-general-principles?search=metastatic%20colorectal&source=search_result&selectedTitle=2%7E150&usage_type=default&display_rank=2 [Accessed 27th March 2024].

4. Van Cutsem E, Nordlinger B, Cervantes A. Advanced colorectal cancer: ESMO Clinical Practice Guidelines for treatment. Ann Oncol. 2010 May;21 Suppl 5:v93-7.

5. Cervantes A, Adam R, Roselló S, Arnold D, Normanno N, Taïeb J, et al. Metastatic colorectal cancer: ESMO Clinical Practice Guideline for diagnosis, treatment and follow-up. Ann Oncol. 2023 Jan;34(1):10-32.

6. Conroy T, Pfeiffer P, Vilgrain V, Lamarca A, Seufferlein T, O'Reilly EM, et al. Pancreatic cancer: ESMO Clinical Practice Guideline for diagnosis, treatment and follow-up. Ann Oncol. 2023 Nov;34(11):987-1002.

7. de Man FM, Goey AKL, van Schaik RHN, Mathijssen RHJ, Bins S. Individualization of Irinotecan Treatment: A Review of Pharmacokinetics, Pharmacodynamics, and Pharmacogenetics. Clin Pharmacokinet. 2018;57(10):1229-54.
8. Yang Y, Zhou M, Hu M, Cui Y, Zhong Q, Liang L, Huang F. UGT1A1*6 and UGT1A1*28 polymorphisms are correlated with irinotecan-induced toxicity: A meta-analysis. Asia Pac J Clin Oncol. 2018;14(5):e479-e89.
9. Zhang X, Yin JF, Zhang J, Kong SJ, Zhang HY, Chen XM. UGT1A1*6 polymorphisms are correlated with irinotecan-induced neutropenia: a systematic review and meta-analysis. Cancer Chemother Pharmacol. 2017;80(1):135-49.
10. Iyer L, Das S, Janisch L, Wen M, Ramirez J, Karrison T, et al. UGT1A1*28 polymorphism as a determinant of irinotecan disposition and toxicity. Pharmacogenomics J. 2002;2(1):43-7.
11. Marcuello E, Altes A, Menoyo A, Del Rio E, Gomez-Pardo M, Baiget M. UGT1A1 gene variations and irinotecan treatment in patients with metastatic colorectal cancer. Br J Cancer. 2004;91(4):678-82.
12. Dean L. Irinotecan Therapy and UGT1A1 Genotype. In: Pratt VM, Scott SA, Pirmohamed M, Esquivel B, Kattman BL, Malheiro AJ, editors. Medical Genetics Summaries. Bethesda (MD)2012.
13. Reizine NM, Danahey K, Truong TM, George D, House LK, Karrison TG et al. Clinically actionable genotypes for anticancer prescribing among >1500 patients with pharmacogenomic testing. Cancer 128(8), 1649–1657 (2022).

14. Hulshof EC, Deenen MJ, Nijenhuis M, Soree B, de Boer-Veger NJ, Buunk AM, et al. Dutch pharmacogenetics working group (DPWG) guideline for the gene-drug interaction between UGT1A1 and irinotecan. Eur J Hum Genet. 2023;31(9):982-7.
15. Karas S, Innocenti F. All you need to know about UGT1A1 genetic testing for patients treated with irinotecan: a practitioner-friendly guide. JCO Oncol. Pract. 18(4), 270–277 (2022).
16. ResearchGate. Irinotecan Metabolism. [Internet]. Available from: Irinotecan Metabolism | Download Scientific Diagram (researchgate.net) [Accessed 13th march 2024]
17. Hulshof EC, de With M, de Man FM, Creemers GJ, Deiman B, Swen JJ, et al. UGT1A1 genotype-guided dosing of irinotecan: A prospective safety and cost analysis in poor metaboliser patients. Eur J Cancer. 2022;162:148-57.
18. Abdullah-Koolmees H, van Keulen AM, Nijenhuis M, Deneer VHM. Pharmacogenetics Guidelines: Overview and Comparison of the DPWG, CPIC, CPNDS, and RNPGx Guidelines. Front Pharmacol. 2020;11:595219.
19. AIOM. Raccomandazioni 2019 per analisi farmacogenetiche. [Internet]. Available from: https://www.aiom.it/wp-content/uploads/2019/10/2019_Racc-analisi-farmacogenetiche_v26.3.2020.pdf [Accessed 13th march 2024].

20. Innocenti F, Schilsky RL, Ram´ırez J, Janisch L, Undevia S, House LK et al. Dose-finding and pharmacokinetic study to optimize the dosing of irinotecan according to the UGT1A1 genotype of patients with cancer. J. Clin. Oncol. 32(22), 2328–2334 (2014).

21. Fujii H, Yamada Y, Watanabe D, Matsuhashi N, Takahashi T, Yoshida K et al. Dose adjustment of irinotecan based on UGT1A1 polymorphisms in patients with colorectal cancer. Cancer Chemother. Pharmacol. 83(1), 123–129 (2019).

22. Catenacci DVT, Chase L, Lomnicki S, Karrison T, de Wilton Marsh R, Rampurwala MM et al. Evaluation of the association of perioperative UGT1A1 genotype-dosed gFOLFIRINOX with margin-negative resection rates and pathologic response grades among patients with locally advanced gastroesophageal adenocarcinoma: a phase 2 clinical trial. JAMA Netw. Open 3(2), e1921290 (2020).

23. Barnett-Griness O, Rennert G, Lejbkowicz F, Pinchev M, Saliba W, Gronich N. Association Between ABCG2, ABCB1, ABCC2 Efflux Transporter Single-Nucleotide Variants and Irinotecan Adverse Effects in Patients With Colorectal Cancer: A Real-Life Study. Clin Pharmacol Ther. 2023 Mar;113(3):704-711. doi: 10.1002/cpt.2833. Epub 2023 Jan 6. PMID: 36537755.

24. Karas S, Mathijssen RHJ, van Schaik RHN, Forrest A, Wiltshire T, Bies RR, Innocenti F. Model-Based Prediction of Irinotecan-Induced Grade 4 Neutropenia in Cancer Patients: Influence of Incorporating Germline Genetic Factors in the Model. Clin Pharmacol Ther. 2024 Feb 12. doi: 10.1002/cpt.3190. Epub ahead of print. PMID: 38344867.

25. De Mattia E, Polesel J, Roncato R, Labriet A, Bignucolo A, Gagno S, Buonadonna A, D'Andrea M, Lévesque E, Jonker D, Couture F, Guillemette C, **Cecchin E#**, Toffoli G. IL15RA and SMAD3 Genetic Variants Predict Overall Survival in Metastatic Colorectal Cancer Patients Treated with FOLFIRI Therapy: A New Paradigm. **Cancers**. 2021 Apr 3;13(7):1705. doi: 10.3390/cancers13071705.

26. De Mattia E, Toffoli G, Polesel J, D'Andrea M, Corona G, Zagonel V, Buonadonna A, Dreussi E, and **Cecchin E**. "Pharmacogenetics of ABC and SLC transporters in metastatic colorectal cancer patients receiving first-line FOLFIRI treatment" **Pharmacogenetics and Genomics**, 23(10):549-57 **2013**.

27. Chen S, Villeneuve L, Jonker D, Couture F, Laverdière I, **Cecchin E**, Innocenti F, Toffoli G, Lévesque E, Guillemette C: "ABCC5 and ABCG1 polymorphisms predict irinotecan-induced severe toxicity in metastatic colorectal cancer patients". **Pharmacogenet Genomics.** 25(12):573-83. 2015

28. Riera P, Páez D. Elucidating the role of pharmacogenetics in irinotecan efficacy and adverse events in metastatic colorectal cancer patients. Expert Opin Drug Metab Toxicol. 2021;17(10):1157–63.

29. Innocenti F, Kroetz DL, Schuetz E, Dolan ME, Ramírez J, Relling M, et al. Comprehensive pharmacogenetic analysis of irinotecan neutropenia and pharmacokinetics. J Clin Oncol. 2009;27(16):2604–14.

30. De Mattia E#, **Cecchin E#**, Montico M, Labriet A, Guillemette C, Dreussi E, Roncato R, Bignucolo A, Buonadonna A, D'Andrea M, Coppola L, Lonardi S, Lévesque E, Jonker D, Couture F, and Toffoli G. Association of STAT-3 rs1053004 and VDRrs11574077 With FOLFIRI-Related Gastrointestinal Toxicity in Metastatic Colorectal Cancer Patients. **2018 Frontiers in Pharmacology** 9:367

31. De Mattia E, Polesel J, Roncato R, Labriet A, Bignucolo A, Dreussi E, Romanato L, Guardascione M, Buonadonna A, D'Andrea M, Lévesque E, Jonker D, Couture F, Guillemette C, **Cecchin E#**, Toffoli G. Germline Polymorphisms in the Nuclear Receptors PXR and VDR as Novel Prognostic Markers in Metastatic Colorectal Cancer Patients Treated With FOLFIRI. **Front Oncol.** 2019 Nov 26;9:1312. doi: 10.3389/fonc.2019.01312. PMID: 31850208; PMCID: PMC6901926.

32. Ichikawa W, Uehara K, Minamimura K, Tanaka C, Takii Y, Miyauchi H, et al. An internally and externally validated nomogram for predicting the risk of irinotecan-induced severe neutropenia in advanced colorectal cancer patients. Br J Cancer. 2015 May 12;112(10):1709-16.

33. Cristina V, Mahachie J, Mauer M, Buclin T, Van Cutsem E, Roth A, et al. Association of Patient Sex With Chemotherapy-Related Toxic Effects: A Retrospective Analysis of the PETACC-3 Trial Conducted by the EORTC Gastrointestinal Group. JAMA Oncol. 2018 Jul 1;4(7):1003-6.

34. Whitley H, Lindsey W. Sex-based differences in drug activity. Am Fam Physician. 2009 Dec 1;80(11):1254-8

35. Routy, B., Le Chatelier, E., Derosa, L., Duong, C. P. M., Alou, M. T., Daillere, R., et al. (2018). Gut microbiome influences efficacy of PD-1-based immunotherapy against epithelial tumors. Science 359 (6371), 91–97.

36. Hopkins, A. M., Kichenadasse, G., McKinnon, R. A., Abuhelwa, A. Y., Logan, J. M., Badaoui, S., et al. (2022). Efficacy of first-line atezolizumab combination therapy in patients with non-small cell lung cancer receiving proton pump inhibitors: Post hoc analysis of IMpower150. Br. J. Cancer 126 (1), 42–47. doi:10.1038/s41416-021-01606-4/

37. Chalabi, M., Cardona, A., Nagarkar, D. R., DhAwAhir ScAlA, A., Rittmeyer, A., et al. (2020). Efficacy of chemotherapy and atezolizumab in patients with nonsmall-cell lung cancer receiving antibiotics and proton pump inhibitors: Pooled post hoc analyses of the OAK and POPLAR trials. Ann. Oncol. 31 (4), 525–531. doi:10.1016/j.annonc.2020.01.006

38. Kichenadasse, G., Miners, J. O., Mangoni, A. A., Karapetis, C. S., Hopkins, A. M., and Sorich, M. J. (2021). Proton pump inhibitors and survival in patients with colorectal cancer receiving fluoropyrimidine-based chemotherapy. J. Natl. Compr. Canc. Netw. 19 (9), 1037–1044. doi:10. 6004/jnccn.2020.7670

39. Saltz, L. B., Clarke, S., Díaz-Rubio, E., Scheithauer, W., Figer, A., Wong, R., et al. (2008). Bevacizumab in combination with oxaliplatin-based chemotherapy as firstline therapy in metastatic colorectal cancer: A randomized phase III study. J. Clin. Oncol. 26 (12), 2013–2019. doi:10.1200/JCO.2007.14.9930

40. Wang, X., Liu, C., Wang, J., Fan, Y., Wang, Z., and Wang, Y. (2017). Proton pump inhibitors increase the chemosensitivity of patients with advanced colorectal cancer. Oncotarget 8 (35), 58801–58808. doi:10.18632/oncotarget.18522

41. van Dijk DP, Bakens MJ, Coolsen MMM, et al. Low skeletal muscle radiation attenuation and visceral adiposity are associated with overall survival and surgical site infections in patients with pancreatic cancer. *Journal of cachexia, sarcopenia and muscle* 2017; 8(2): 317-26.

42. Black D, Mackay C, Ramsay G, et al. Prognostic Value of Computed Tomography: Measured Parameters of Body Composition in Primary Operable Gastrointestinal Cancers. *Ann Surg Oncol* 2017; 24(8): 2241-51.

43. Grossberg AJ, Chamchod S, Fuller CD, et al. Association of Body Composition With Survival and Locoregional Control of Radiotherapy-Treated Head and Neck Squamous Cell Carcinoma. *JAMA oncology* 2016; 2(6): 782-9.

44. Bundred J, Kamarajah SK, Roberts KJ. Body composition assessment and sarcopenia in patients with pancreatic cancer: a systematic review and meta-analysis. *HPB (Oxford)* 2019; 21(12): 1603-12.

45. Van Dijk DP, Krill M, Farshidfar F, et al. Host phenotype is associated with reduced survival independent of tumor biology in patients with colorectal liver metastases. *Journal of Cachexia, Sarcopenia and Muscle* 2018.

46. Henricks LM, Lunenburg C, de Man FM, Meulendijks D, Frederix GWJ, Kienhuis E et al. DPYD genotype-guided dose individualisation of fluoropyrimidine therapy in patients with cancer: a prospective safety analysis. Lancet Oncol. 19(11), 1459–1467 (2018)

47. Knikman JE, Wilting TA, Lopez-Yurda M, Henricks LM, Lunenburg C, de Man FM, et al. Survival of Patients With Cancer With DPYD Variant Alleles and Dose-Individualized Fluoropyrimidine Therapy-A Matched-Pair Analysis. J Clin Oncol. 2023;41(35):5411-21.

48. Cancer ResearchUK. Survival for bowel cancer. [Internet]. Available from: https://www.cancerresearchuk.org/about-cancer/bowel-cancer/survival [Accessed 15th march 2024].

49. Cancer ResearchUK. Survival for pancreatic cancer. [Internet]. Available from: https://www.cancerresearchuk.org/about-cancer/pancreatic-cancer/survival [Accessed 15th march 2024].

50. Mhandire DZ, Goey AKL. The value of pharmacogenetics to reduce drug-related toxicity in cancer patients. Mol. Diagn. Ther. 26(2), 137–151 (2022).

51. Lau-Min KS, Varughese LA, Nelson MN, Cambareri C, Reddy NJ, Oyer RA et al. Preemptive pharmacogenetic testing to guide chemotherapy dosing in patients with gastrointestinal malignancies: a qualitative study of barriers to implementation. BMC Cancer 22(1), 47 (2022).

APPENDIX A (dutch):

Wordt er (deels) prospectief extra patiëntdata verzameld (naast de standaard zorg)?

JA → **Altijd patiënt om toestemming vragen. Zie verder: 1. Conclusie**

NEE ↓

1. Wordt de patient om toestemming gevraagd?

JA → **1. Conclusie:** Studie mag worden uitgevoerd. **Voorwaarden:**

- Toestemmingsprocedure RvB doorlopen verplicht
- EPD toegang toegestaan voor partijen zoals genoemd in de PIF (beperkt en alleen-lezen)
- Studieovereenkomst nodig (contract of data sharing agreement)
- Meld studie deelname in dossier patiënt
- Geen direct herleidbare persoonsgegevens opnemen in analyse file (hooguit gecodeerd)
- Sleutelfile opslaan op ziekenhuisserver op afgesproken plaats (mag niet naar extern)
- *Indien extern persoon toegang behoeft:* toegangsovereenkomst nodig (PNIL / geheimhoudingsverklaring) + toevoeging van deze persoon aan studieteam in studiedossier

NB. toestemming voor een specifieke studie overschrijft 'algemeen bezwaar' t.b.v. inzage in het EPD

NEE ↓

2. Is het mogelijk anonieme gegevens te gebruiken? (NB: gecodeerd is NIET hetzelfde als anoniem)

JA → **2. Conclusie:** Gebruik van anonieme gegevens is toegestaan. **Voorwaarden:**

- Toestemmingsprocedure RvB doorlopen verplicht
- Data extractie direct door medewerker met behandelrelatie (overzetten in anonieme dataset voor onderzoeker) ofwel via geautomatiseerd systeem (zie inzet links onderaan pagina)
- Geen toegang tot EPD voor niet-behandelaar / externe onderzoeker
- Geen datasharing agreement nodig, wel afspraken over 'eigendom' resultaten
- Houd registratie bij van welke patiënten voor welke studie data is gebruikt (verwerkingsregister)

NB: Gegevens uit het EPD van patiënten die een algemeen bezwaar hebben gemaakt tegen gebruik mogen NIET worden gebruikt voor wetenschappelijk onderzoek. Ook niet anoniem!

NEE ↓

3. Is het redelijkerwijs mogelijk om de patienten alsnog om toestemming te vragen?

JA → **3. Conclusie:** Studie mag worden uitgevoerd. **Voorwaarden:**

- Vraag toestemming van de patiënt voor gebruik van diens data. Daarna gelden voorwaarden zoals genoemd bij **1. Conclusie**

(→ 1. Conclusie)

NEE ↓

4. Is er een valide grondslag voor NEE?

JA → **Ja, namelijk:** (1 kiezen)

1. Onevenredige inspanning (vb. Grote aantallen / behandeling in ver verleden)
2. In redelijkheid niet mogelijk (vb. veel patiënten overleden, psychische belasting/schade voor patiënt, geen reactie na tenminste 2 reminders)
3. Steekproeftrekken nodig (screenen)
4. toestemmingsvraag kan niet zinvol worden gesteld omdat onderzoek zich in eerste voorbereidende fase bevind (pilot/biobank), **voorwaarden**:

↓

4. Conclusie: **Studie mag worden uitgevoerd onder voorwaarden:**

- Toestemmingsprocedure RvB doorlopen verplicht
- Data extraheren bij voorkeur via geautomatiseerd systeem (zie inzet links)
- *Indien geautomatiseerde extractie niet mogelijk:* zo beperkt mogelijke inzage, alleen binnen muren ziekenhuis, bij voorkeur door medewerker met behandelrelatie, altijd onder verantwoordelijkheid van lokale PI
- *Indien EPD-toegang nodig voor externe onderzoeker:* externe tekent toegangsovereenkomst (PNIL of geheimhoudingsverklaring) en krijgt zo beperkt mogelijke inzage, altijd onder verantwoordelijkheid van lokale PI
- Geen direct herleidbare persoonsgegevens opnemen in analyse file (hooguit gecodeerd)
- Sleutelfile opslaan op ziekenhuisserver op afgesproken plaats (mag niet naar extern)
- *Indien de data buiten de ziekenhuismuren wordt gedeeld:* datasharing agreement nodig
- *Indien onderbouwing 3:* **1)** niet méér gegevens inzien dan voor steekproeftrekking noodzakelijk, **2)** bij patiënten wiens data uiteindelijk wél wordt gebruikt alsnog toestemming vragen (zie verder 1. Conclusie)
- *Indien onderbouwing 4:* **1)** doel en tijdstip inzage schriftelijk vastleggen (voor matching EPD-logging) **2)** in protocol vastleggen: **A.** waarom zonder eerst inzage beperkt aantal persoonsgegevens van beperkt aantal personen, **B.** patiënten kunnen niet zinvol worden aangeschreven omdat nog onvoldoende kan worden omschreven voor welk onderzoek toestemming wordt gevraagd

NB: Gegevens uit het EPD van patiënten die een algemeen bezwaar hebben gemaakt tegen gebruik mogen NIET worden gebruikt voor wetenschappelijk onderzoek. Ook niet anoniem! Enige uitzondering hierop is wanneer de patiënt voor een specifieke studie wél informed consent afgeeft, dan mag diens data wél gebruikt worden voor die studie.

NEE ↓

5. Conclusie: **Studie mag NIET worden uitgevoerd**

Geautomatiseerde data extractie in 3 stappen:
1) beschrijf in protocol/data managementplan welke data waarvoor verzameld moet worden
2) bepaal samen met Functioneel Applicatiebeheerder welke velden in EPD dit zijn
3) vraag Business Intelligence (BI) een query met anoniem dataextract te leveren ná RvB toestemming

STATISTICAL ANALYSIS PLAN version 3.0 dd. 25-02-2025

The structure of this document was based on: Gamble C, Krishan A, Stocken D, et al. Guidelines for the Content of Statistical Analysis Plans in Clinical Trials. JAMA. 2017;318(23):2337–2343. doi:10.1001/jama.2017.18556

This statistical analysis plan relates to:

Study protocol title: Survival Analysis of Cancer Patients with *UGT1A1* Variant Alleles and Dose-Individualized Irinotecan Therapy and Other Irinotecan-related Sub-analyses

Short title: IRI-DATABASE

Study Protocol version: 2.1 (dd. 29-04-2024)

IRB Approval date: 07-05-2024

Authors

**Name**	**Function**	**Signature**	**Date**
S.L.J. Peeters	PhD Candidate, Coordinating investigator		25-02-2025
N. Heersche	PhD Candidate, Coordinating investigator		25-02-2025
S. Böhringer	Study Statistician		25-02-2025

Document approval

**Name**	**Function**	**Signature**	**Date**
M.J. Deenen	Principal Investigator		25-02-2025
R.H.J. Mathijssen	Local Principal Investigator		25-02-2025
H. Gelderblom	Local Principal Investigator		25-02-2025
H.J. Guchelaar	Local Principal Investigator		25-02-2025

1 INTRODUCTION

1.1 Background and rationale

This study expands on the results of Hulshof et al. (EJC 2022), which focused on the safety and feasibility of *UGT1A1* genotype-guided dosing of irinotecan. The impact of *UGT1A1* genotype-guided dosing on treatment efficacy in UGT1A1 poor metabolizers (PM) is largely unknown. Therefore, the main aim of the present study is to assess the impact of *UGT1A1* genotype-guided dosing of irinotecan on survival outcomes. A traditional approach using a randomized clinical trial comparing survival in UGT1A1 PM with a full dose would be unethical and unfeasible because of the known increased risk of severe irinotecan-related toxicity in UGT1A1 PM. Therefore, we will compare survival outcomes of UGT1A1 PM treated with an initial 30% dose reductions to survival outcomes of UGT1A1 intermediate and extensive metabolizers (IM and EM) treated with full irinotecan dosages (100% irinotecan dose intensity).

1.2 Research question

Are survival outcomes (progression-free survival (PFS) and overall survival (OS)) of UGT1A1 poor metabolizers (PM) that received *UGT1A1* genotype-guided irinotecan dosing comparable to the survival outcomes of UGT1A1 intermediate/extensive (IM/EM) metabolizers treated with a full irinotecan dose?

1.3 Primary objective

To determine the effect of an initial 30% dose reduction of irinotecan in UGT1A1 PM compared to a full irinotecan dose (100% dose intensity) in UGT1A1 IM/EM on PFS.

1.4 Secondary objectives

- To determine the effect of an initial 30% irinotecan dose reduction in UGT1A1 PM compared to a full irinotecan dose (100% dose intensity) in UGT1A1 IM/EM on OS.
- To compare the PFS and OS between intermediate metabolizers (IM) and extensive metabolizers (EM) treated with a full irinotecan dose.
- To assess the incidence of overall severe (grade ≥3) irinotecan-related toxicity in UGT1A1 PM and IM/EM patients that received *UGT1A1* genotype-guided dosing of irinotecan.

2 STUDY METHODS

2.1 Study design

Retrospective, multicentre, non-randomized, cohort study.

The Medical research Ethics Committee United (MEC-U, Nieuwegein, the Netherlands) approved the study protocol and declared the study not to be subject to the Medical Research Involving Human Subjects Act (MEC-U study registration number W24.109). Local approval was obtained from all participating study sites. Given the retrospective character of the study and the anticipated size of the patient population a waiver was provided for informed consent.

Study sites: Catharina Hospital Eindhoven, Erasmus Medical Centre Rotterdam, Leiden University Medical Centre, Jeroen Bosch Hospital Den Bosch, Maasstad Hospital Rotterdam, Reinier de Graaf Gasthuis Delft. All study sites are located in the Netherlands.

2.2 Intervention group

The intervention group is defined as UGT1A1 PM treated with *UGT1A1* genotype-guided dosing of irinotecan, that is, an initial 30% dose reduction of irinotecan.

UGT1A1 PM, IM and EM are classified following the DPWG guideline (accessed on 25-02-2025): Dutch pharmacogenetics working group (DPWG) guideline for the gene-drug interaction between UGT1A1 and irinotecan - PubMed

2.3 Control group

The control group is defined as UGT1A1 intermediate or extensive metabolizers with full irinotecan doses, that is, treated with an initial 100% dose intensity of irinotecan.

2.4 Sample size

Sample size was calculated for a non-inferiority analysis of a Cox proportional hazards model was performed to assess the required sample size for statistically assessing non-inferiority in endpoints PFS and OS. The non-inferiority margin was varied between 1.1 and 1.4 assuming a true hazard ratio of 1. Marginal event rate was varied between 0.6 and 0.8, group allocation between 0.1 and 0.3. Either power was calculated for given sample size and margin, or required sample size for given margin and power at 80% (table below). Based on these calculations, for several realistic scenarios a non-inferiority margin of 1.4 is required, which is considered to be large, clinically. Apart from a formal non-inferiority margin of 1.4, results will also be interpreted descriptively based on confidence intervals derived from the regression models, to allow for relevant clinical interpretation when a lower margin would have been possible.

Calculated 13.02.2024 with PASS 15.0.13

Non-Inferiority Tests for Two Survival Curves Using Cox's Proportional Hazards Model

Numeric Results with Ha: HR < HRni

Power	Total Sample Size N	Control Sample Size N1	Trtmnt Sample Size N2	Prop'n Control N1/N P1	Hazard Ratio h2/h1 HR	Non Inf Hazard Ratio HRni	Control Prob Event Pev1	Trtmnt Prob Event Pev2	Control Events E1	Trtmnt Events E2	Alpha
0,1183	434	391	43	0,900	1,000	1,100	0,600	0,600	234,6	26,0	0,050
0,2230	434	391	43	0,900	1,000	1,200	0,600	0,600	234,6	26,0	0,050
0,4937	434	391	43	0,900	1,000	1,400	0,600	0,600	234,6	26,0	0,050
0,1258	434	391	43	0,900	1,000	1,100	0,700	0,700	273,7	30,4	0,050
0,2446	434	391	43	0,900	1,000	1,200	0,700	0,700	273,7	30,4	0,050
0,5456	434	391	43	0,900	1,000	1,400	0,700	0,700	273,7	30,4	0,050
0,1331	434	391	43	0,900	1,000	1,100	0,800	0,800	312,8	34,7	0,050
0,2658	434	391	43	0,900	1,000	1,200	0,800	0,800	312,8	34,7	0,050
0,5933	434	391	43	0,900	1,000	1,400	0,800	0,800	312,8	34,7	0,050
0,1994	559	390	169	0,700	1,000	1,100	0,600	0,600	234,0	101,2	0,050
0,4553	559	390	169	0,700	1,000	1,200	0,600	0,600	234,0	101,2	0,050
0,8817	559	390	169	0,700	1,000	1,400	0,600	0,600	234,0	101,2	0,050
0,2179	559	390	169	0,700	1,000	1,100	0,700	0,700	273,0	118,1	0,050
0,5042	559	390	169	0,700	1,000	1,200	0,700	0,700	273,0	118,1	0,050
0,9208	559	390	169	0,700	1,000	1,400	0,700	0,700	273,0	118,1	0,050
0,2359	559	390	169	0,700	1,000	1,100	0,800	0,800	312,0	135,0	0,050
0,5497	559	390	169	0,700	1,000	1,200	0,800	0,800	312,0	135,0	0,050
0,9475	559	390	169	0,700	1,000	1,400	0,800	0,800	312,0	135,0	0,050
0,1606	870	783	87	0,900	1,000	1,100	0,600	0,600	469,8	52,1	0,050
0,3461	870	783	87	0,900	1,000	1,200	0,600	0,600	469,8	52,1	0,050
0,7455	870	783	87	0,900	1,000	1,400	0,600	0,600	469,8	52,1	0,050
0,1737	870	783	87	0,900	1,000	1,100	0,700	0,700	548,1	60,8	0,050
0,3837	870	783	87	0,900	1,000	1,200	0,700	0,700	548,1	60,8	0,050
0,8010	870	783	87	0,900	1,000	1,400	0,700	0,700	548,1	60,8	0,050
0,1865	870	783	87	0,900	1,000	1,100	0,800	0,800	626,4	69,5	0,050
0,4198	870	783	87	0,900	1,000	1,200	0,800	0,800	626,4	69,5	0,050
0,8454	870	783	87	0,900	1,000	1,400	0,800	0,800	626,4	69,5	0,050
0,3040	1119	783	336	0,700	1,000	1,100	0,600	0,600	469,8	201,5	0,050
0,6986	1119	783	336	0,700	1,000	1,200	0,600	0,600	469,8	201,5	0,050
0,9906	1119	783	336	0,700	1,000	1,400	0,600	0,600	469,8	201,5	0,050
0,3364	1119	783	336	0,700	1,000	1,100	0,700	0,700	548,1	235,1	0,050
0,7561	1119	783	336	0,700	1,000	1,200	0,700	0,700	548,1	235,1	0,050
0,9962	1119	783	336	0,700	1,000	1,400	0,700	0,700	548,1	235,1	0,050
0,3677	1119	783	336	0,700	1,000	1,100	0,800	0,800	626,4	268,7	0,050
0,8038	1119	783	336	0,700	1,000	1,200	0,800	0,800	626,4	268,7	0,050
0,9985	1119	783	336	0,700	1,000	1,400	0,800	0,800	626,4	268,7	0,050

Power	Total Sample Size N	Trtmnt Sample Size N1	Control Sample Size N2	Prop'n Trtmnt N1/N P1	Hazard Ratio h2/h1 HR	Non Inf Hazard Ratio HRni	Trtmnt Prob Event Pev1	Control Prob Event Pev2	Trtmnt Events E1	Control Events E2	Alpha
0,8030	1020	102	918	0,100	1,000	1,400	0,600	0,600	61,2	550,8	0,050
0,8013	870	87	783	0,100	1,000	1,400	0,700	0,700	60,9	548,1	0,050
0,8007	760	76	684	0,100	1,000	1,400	0,800	0,800	60,8	547,2	0,050
0,8002	434	130	304	0,300	1,000	1,400	0,600	0,600	78,0	182,4	0,050
0,8020	374	112	262	0,300	1,000	1,400	0,700	0,700	78,4	183,4	0,050
0,8019	327	98	229	0,300	1,000	1,400	0,800	0,800	78,4	183,2	0.050

References

Chow, S.C., Shao, J., Wang, H. 2008. Sample Size Calculations in Clinical Research, 2nd Edition. Chapman & Hall/CRC.

Schoenfeld, David A. 1983. 'Sample Size Formula for the Proportional-Hazards Regression Model', Biometrics, Volume 39, Pages 499-503.

Report Definitions

Power is the probability of rejecting a false null hypothesis. Power should be close to one.
N is the total sample size.
N1 and N2 are the sample sizes of the control and treatment groups.
P1 is the proportion of the total sample that is in the treatment group, group 1.
HR is the hazard ratio: h2/h1.
HRni is the non-inferiority hazard ratio.
Pev1 and Pev2 are the probabilities of an event in the control and the treatment groups.
E1 and E2 are the number of events required in the control and the treatment groups.
Alpha is the probability of a type one error: rejecting a true null hypothesis.

Summary Statements
A non-inferiority test of whether the hazard ratio with an overall sample size of 1020 subjects (of which 102 are in the treatment group and 918 are in the control group) achieves 80% power at a 0,050 significance level when the hazard ratio is actually 1,000. The non-inferiority ratio is 1,400. The number of events required to achieve this power is 612,0. It is anticipated that the proportion of subjects observed with the event during the study is 0,600 for the control group and 0,600 for the treatment group. These results assume that the hazard ratio is constant throughout the study and that Cox proportional hazards regression or the non-inferiority logrank test is used to analyze the data.

2.5 Timing of outcome assessments

Eligible patients will have started irinotecan-based treatment between August 2017-April 2024. Data is collected between September 2024-April 2025. Progression is assessed as part of standard care. Patients will be followed-up till study end. Study end is defined as the last date available in the electronic patient dossier (EPD) at the moment of data collection by the data collector. Patient status (alive/deceased) is obtained through the EPD and/or the citizen service number (BSN) registry.

2.6 Timing of final analysis

The final analysis of this study will start as soon as all subjects have reached the end of study and data collection has been completed. The final analysis is expected to take place around April-May 2025.

3 STATISTICAL PRINCIPLES

3.1 Level of significance and multiple testing

All applicable statistical tests will be 2-sided and will be performed using a 5% significance level. All confidence intervals presented will be 95% and 2-sided. No correction for multiple testing (e.g., Bonferroni correction) was performed, as a single primary is defined (PFS, see below). Non-primary analyses are considered explorative.

3.2 Adherence to the intervention

Adherence to the intervention is defined per group:

- UGT1A1 poor metabolizers: a dose intensity of 70% of irinotecan in cycle 1.
- UGT1A1 intermediate and extensive metabolizers: full dose (100% dose intensity) of irinotecan in cycle 1.

It is allowed for the above-described percentages of 100% and 70% to maximally deviate ±10% due to rounding of the irinotecan dosages during preparation of the chemotherapy infusions.

Adherence to the intervention will be shown in the Consort diagram based on the CONSORT 2010 flow diagram (http://www.consort-statement.org/)

3.3 Protocol deviations

Patients that received a starting dose intensity of irinotecan higher or lower than described under 3.2.

3.4 Definition of analysis populations

A **per-protocol population analysis** will performed as the primary analysis in order to assess the effect of an actual initial 30% dose reduction (in contrast with an intended reduction) in PM in the survival analysis. In the per-protocol analysis, patients that encountered protocol deviations (irinotecan dosage not according to *UGT1A1* genotype in cycle 1) are excluded.

An **intention-to-treat (ITT) analysis** will be performed as a sensitivity analysis. The intention-to-treat population includes eligible patients, regardless of their received irinotecan dosage in cycle 1.

4 TRIAL POPULATION

4.1 Screening data

The following summaries will be presented for all screened patients: Enrollment: the number of patients screened, the number of patients eligible/included, the number of screened patients not eligible and the reason for non-eligibility, the number of patients included in the analysis and the number of patients excluded from the analysis and the reason for exclusion.

4.2 Eligibility

<u>Inclusion criteria:</u>

Previous cohort (Hulshof et al. EJC 2022):

- Inclusion criteria:
    - Patients that participated in the study of Hulshof et al. (EJC 2022) (inclusion period took place between August 2017-December 2020)
      Inclusion criteria of Hulshof et al. (EJC 2022) were as follows:
        - Age ≥ 18 years
        - A pathologically confirmed malignancy intended to be treated with irinotecan at a dose of ≥ 180 mg/m2 or 450-600 mg flat dose
        - Written informed consent
        - A WHO performance status of 0, 1 or 2
        - Acceptable safety laboratory values:
          Absolute neutrophil count (ANC) ≥ 1.5 x 109/L o Platelet count ≥ 100 x 109/L
          Serum bilirubin ≤ 1.5 x upper limit of normal (ULN)
          ALT and AST ≤ 2.5 x ULN; in case of liver metastases ALT and AST ≤ 5 x ULN
          Renal function (eGFR) ≥ 50 ml/min or creatinine ≤ 1.5 x ULN

      Exclusion criteria of Hulshof et al. (EJC 2022) were as follows:
        - Prior treatment with irinotecan
        - Known substance abuse
        - Psychotic disorders or other diseases expected to interfere with the study or the patient's safety
        - Asian origin
        - The use of (over the counter) medication or (herbal) supplements that were known to interact with irinotecan (e.g. by induction of inhibition of CYP3A4)
- Exclusion criteria: Primary tumor type other than colorectal cancer or pancreatic cancer

Expansion cohort:

- Inclusion criteria:
    - Age ≥ 18 years
    - Having received treatment with systemic irinotecan, regardless of dosage, WHO performance status or laboratory values at baseline.
    - First irinotecan cycle received in the period of December 2020-April 2024.
- Exclusion criteria:
    - Missing *UGT1A1* genotyping data
    - Opt-out registration present for participation in scientific research
    - Treatment with nanoliposomal irinotecan
    - Primary tumor types other than colorectal cancer or pancreatic cancer

4.3 Baseline patient characteristics

Categorical data will be summarized by numbers and percentages. Continuous data will be summarized by mean, SD and range if data are normal and median, IQR and range if data are skewed. Tests of statistical significance will not be undertaken for baseline characteristics; rather the clinical importance of any imbalance will be noted.

Table baseline characteristics

	**UGT1A1 PM**<br>**n=**	**UGT1A1 IM/EM**<br>**n=**	**All patients**<br>**n=**
**Sex**<br>- Male<br>- Female	n (%)	n (%)	n (%)
**Age, years**	Median or mean (IQR or SD)	Median or mean (IQR or SD)	Median or mean (IQR or SD)
**Ethnic origin**<br>European<br>Middle-Eastern<br>North-African<br>African (sub-Saharan)<br>Asian<br>Hispanic<br>Other	n (%)	n (%)	n (%)
**Primary cancer type**<br>- Colorectal cancer<br>- Pancreatic cancer	n (%)	n (%)	n (%)
**Cancer stage**<br>I<br>II<br>III<br>IV	n (%)	n (%)	n (%)
**WHO performance**<br>0-1<br>≥2	n (%)	n (%)	n (%)
**Smoking status**<br>- Never/Former<br>- Current	n (%)	n (%)	n (%)
**BSA, m2**	Median or mean (IQR or SD)	Median or mean (IQR or SD)	Median or mean (IQR or SD)
**Previous treatment for current cancer**<br>- Chemotherapy<br>- Surgery<br>- Radiotherapy<br>- None	n (%)	n (%)	n (%)
**Previous treatment with irinotecan**<br>- No<br>- Yes	n (%)	n (%)	n (%)
**Current treatment regimen**<br>- FOLFIRI<br>- FOLFIRI + bevacizumab<br>- FOLFIRINOX<br>- mFOLFIRINOX<br>- FOLFOXIRI + bevacizumab<br>- FOLFOXIRI<br>- Irinotecan + panitumumab<br>- Irinotecan monotherapy low dose<br>- Irinotecan monotherapy high dose<br>- Other	n (%)	n (%)	n (%)
**Relative dose intensity (%) in first cycle**	Median or mean (IQR or SD)	Median or mean (IQR or SD)	Median or mean (IQR or SD)

Table genotype frequencies

**Genotype status**	**n=**	**(%)**	**HWE p-value**	**MAF**
***UGT1A1**28**<br>**- WT**<br>**- HET**<br>**- HOM**<br>**- not assessed**				
***UGT1A1**93**<br>**- WT**<br>**- HET**<br>**- HOM**<br>**- not assessed**				
***UGT1A1**6**<br>**- WT**<br>**- HET**<br>**- HOM**<br>**- not assessed**				
***UGT1A1**36**<br>**- WT**<br>**- HET**<br>**- HOM**<br>**- not assessed**				
***UGT1A1**37**<br>**- WT**<br>**- HET**<br>**- HOM**<br>**- not assessed**				
***UGT1A1**80**<br>**- WT**<br>**- HET**<br>**- HOM**<br>**- not assessed**				

HWE = Hardy Weinberg Equilibrium, MAF= minor allele frequency, WT = wild type, HET = heterozygous, HOM = homozygous

5 ANALYSIS

5.1 Outcome definitions

Primary outcome:

- Progression-free survival (PFS): time between initiation of irinotecan treatment and first signs of disease progression or recurrence by either clinical signs or radiological imaging (RECIST 1.1), or death from any cause, whichever came first.

Secondary outcome:

- Overall survival (OS): time between initiation of irinotecan treatment and death from any cause.

5.2 Analysis methods

5.2.1. Censoring

Patients that have not experienced an event (progression/death) at study end will be censored:

- PFS: Patients are censored at the last date known to be alive and progression-free.
- OS: Patients are censored at the last date known to be alive.

Censoring distribution and median follow-up

Median and range of follow-up will be reported using the method described by Schemper and Smith (Control Clin Trial 1996). A censoring distribution will be estimated by plotting reverse Kaplan-Meier graphs to determine whether censoring is independent/non-informative. In the case of informative censoring, probabilistic methods will be used to correct for informative censoring (e.g. inverse probability of censoring weighted (IPCW)).

5.2.2. Kaplan Meier curves and estimate

Kaplan-Meier curves with pointwise 95% confidence intervals will be plotted to depict the survival curves of the intervention and control group. The median PFS and OS will be reported for each group (PM and IM/EM) with a 95% confidence interval.

Global test: To test whether there is a difference between the two survival curves (PM vs. IM/EM), a log-rank test will be performed (under the proportional hazards assumption). A log-rank test stratified for tumor type (CRC and PC) will be performed.

5.2.3. Cox proportional hazards regression model

To estimate the effect size, Hazard Ratio (HR) with 95% confidence interval, of the initial 30% irinotecan dose reduction in PM and of other relevant covariates, Cox proportional hazards regression will be performed.

Cox proportional hazards (PH) assumption testing

The PH assumption will be verified for each covariate by graphically assessing log-minus-log plots. If the PH assumption cannot be adequately verified by log-minus-log plots, the PH assumption will be additionally tested using Schoenfield residuals and time-depended covariate effect will be used, when necessary.

5.2.4. Stratified analyses

The stratified Cox regression is an approach that assumes a common HR, but different baseline hazards. Two different tumor types will be included in the analysis, colorectal cancer (CRC) and pancreatic cancer (PC), for which it is unrealistic to assume an identical baseline hazard. Therefore, the multivariable Cox PH regression analysis will be performed as a stratified Cox model using tumor type as strata (CRC and PC).

5.3 Adjustments for covariates

Univariable and multivariable Cox PH regression analyses will be performed to adjust for relevant covariates. Hazard Ratios (HRs) and their corresponding 95% confidence intervals will be calculated. Covariates with univariable HRs that have $p<0.1$ will be entered into the multivariable Cox PH regression analysis which is used for the primary analysis.

Fixed covariates at baseline to be assessed by univariable Cox PH regression:

- UGT1A1 phenotype (PM vs. IM/EM)
- Sex (Male vs. female)
- Age (<65 vs. ≥65)
- Ethnicity (European vs. other)
- Smoking status (current vs. never/former)
- WHO performance status (0-1 vs. ≥2)
- Tumor stage (stage I vs. stage II vs. stage III vs. stage IV)
- Tumor type (CRC/PC)
- Treatment regimen
- Previous chemotherapy (yes/no)
- Number of previous chemotherapy lines (0/1/2/3)
- Previous irinotecan treatment
- Previous radiotherapy
- Previous surgery
- Irinotecan relative dose intensity (RDI) in cycle 1 (only in intention-to-treat analysis)

Fixed covariates at baseline to be included in multivariable Cox PH regression:

- UGT1A1 phenotype (PM vs. IM/EM)
- Covariates with $p < 0.1$ in the univariable Cox PH regression analysis

The multivariable, stratified Cox PH regression with strata for tumor type (CRC vs. PC) constitutes the primary analysis.

For the final multivariable, stratified Cox PH regression model, survival curves will be derived (supplementary).

5.4 Subgroup analysis

The following pre-specified subgroup analyses will be performed:

- PM vs. IM/EM in colorectal cancer patients only for PFS and OS;
- PM vs. IM/EM in pancreatic cancer patients only for PFS and OS;
- IM vs. EM in colorectal and pancreatic cancer patients (stratified analysis) for PFS and OS.

5.5 Competing risks and multistates

No competing risk model will be used for this survival study as time to progression, recurrence or death events are combined into one combined endpoint (PFS). Death events are collected as 'death from any cause', omitting competing causes of death. No multistate model will be used.

5.6 Sensitivity analysis

Sensitivity analyses will be performed to explore the robustness of results. Planned sensitivity analyses:

- Intention-to-treat analysis for PFS and OS;
- Subgroup analyses in PC and CRC for PFS and OS;

5.7 Missing data

The percentages of patients with missing data in any of the covariates to be adjusted for in the analysis (see 5.3) will be calculated and reported. If ≤ 5% of patients have missing data, patients with missing data will be excluded from the primary analysis and a complete case analysis will be performed. If > 5% of patients have missing data, multiple imputation will be used.

Missing outcome data (i.e., PFS, OS, toxicity) will not be imputed. For PFS and OS, censored patients will be included in the primary analysis.

5.8 Harms

The proportions of patients with overall severe (CTCAE grade 3, 4 or 5) toxicity for the first three cycles of treatment will be compared between UGT1A1 PM with an initial 30% irinotecan dose reduction (intervention) and fully dosed IM/EM (control) based on the confidence interval. Additionally, relative dose intensity and treatment modifications of irinotecan after cycle 1 will be reported and compared between groups.

5.9 Statistical software

The analyses will be carried out using SPSS version 29.0.1.0. Other packages such as R or SAS may be used if necessary. Plots will be produced in Graphpad Prism version 9.
